# Earliest evidence of herd-living and age segregation amongst dinosaurs

**DOI:** 10.1038/s41598-021-99176-1

**Published:** 2021-10-21

**Authors:** Diego Pol, Adriana C. Mancuso, Roger M. H. Smith, Claudia A. Marsicano, Jahandar Ramezani, Ignacio A. Cerda, Alejandro Otero, Vincent Fernandez

**Affiliations:** 1 CONICET, Museo Paleontológico Egidio Feruglio, Av. Fontana 140, 9100 Trelew, Argentina; 2 IANIGLA, CCT-CONICET-Mendoza, Adrián Ruiz Leal S/N - Parque Gral. San Martín 5500, C.C.330, Mendoza, Argentina; 3 Evolutionary Studies Institution, University of Witwatersrand, Johannesburg, South Africa; 4 CONICET-UBA IDEAN, Departamento de Ciencias Geológicas, Facultad de Ciencias Exactas Y Naturales, Universidad de Buenos Aires, Intendente Güiraldes 2160, Ciudad Universitaria C1428EHA, Buenos Aires, Argentina; 5 Department of Earth, Atmospheric and Planetary Sciences, Massachusetts Institute of Technology, Cambridge, MA 02139 USA; 6 CONICET, Instituto de Investigación en Paleobiología Y Geología, Universidad Nacional de Río Negro, Museo Carlos Ameghino, Belgrano 1700, Paraje Pichi Ruca (Predio Marabunta), Cipolletti, Río Negro Argentina; 7 CONICET, División Paleontología de Vertebrados, Museo de La Plata, Paseo del Bosque s/n (1900) La Plata, Buenos Aires, Argentina; 8 European Synchrotron Radiation Facility, 71 avenue des Martyrs, 38000 Grenoble, France

**Keywords:** Evolution, Palaeontology

## Abstract

Sauropodomorph dinosaurs dominated the herbivorous niches during the first 40 million years of dinosaur history (Late Triassic–Early Jurassic), yet palaeobiological factors that influenced their evolutionary success are not fully understood. For instance, knowledge on their behaviour is limited, although herding in sauropodomorphs has been well documented in derived sauropods from the Late Jurassic and Cretaceous. Here we report an exceptional fossil occurrence from Patagonia that includes over 100 eggs and skeletal specimens of 80 individuals of the early sauropodomorph *Mussaurus patagonicus*, ranging from embryos to fully-grown adults, with an Early Jurassic age as determined by high-precision U–Pb zircon geochronology. Most specimens were found in a restricted area and stratigraphic interval, with some articulated skeletons grouped in clusters of individuals of approximately the same age. Our new discoveries indicate the presence of social cohesion throughout life and age-segregation within a herd structure, in addition to colonial nesting behaviour. These findings provide the earliest evidence of complex social behaviour in Dinosauria, predating previous records by at least 40 My. The presence of sociality in different sauropodomorph lineages suggests a possible Triassic origin of this behaviour, which may have influenced their early success as large terrestrial herbivores.

## Introduction

Soon after dinosaurs originated, early sauropodomorphs (forerunners of the gigantic quadrupedal sauropods) underwent a remarkable adaptive radiation landmarked by the acquisition of herbivory^1–4^, large body sizes^5^, and high taxonomic diversity and specimen abundance^2,4,6^. By the end of the Triassic, sauropodomorphs had replaced other herbivores (therapsids and other archosaurs) and were the most abundant tetrapods in many terrestrial ecosystems^1,4,6^. Sauropodomorphs were subsequently unaffected by the Triassic–Jurassic extinction event (ca. 200 Ma), which left them as the only large herbivores in terrestrial ecosystems of the Early Jurassic^4,6^. The predominance of early sauropodomorph dinosaurs in terrestrial ecosystems extended for almost 40 million years^7^ (ca. 220–180 Ma, Norian–Pliensbachian^8^). Proposed reasons for their early success^3,9^ include their ability to opportunistically adapt to niches left empty after the extinction of other herbivores^1^ or to out-perform their competition with superior high-browsing herbivory, large body size, and rapid growth rates^4,9–13^. Behaviour has not been regarded as playing a role in the early success of these dinosaurs due to the scarcity of relevant information.

**Figure 1:**
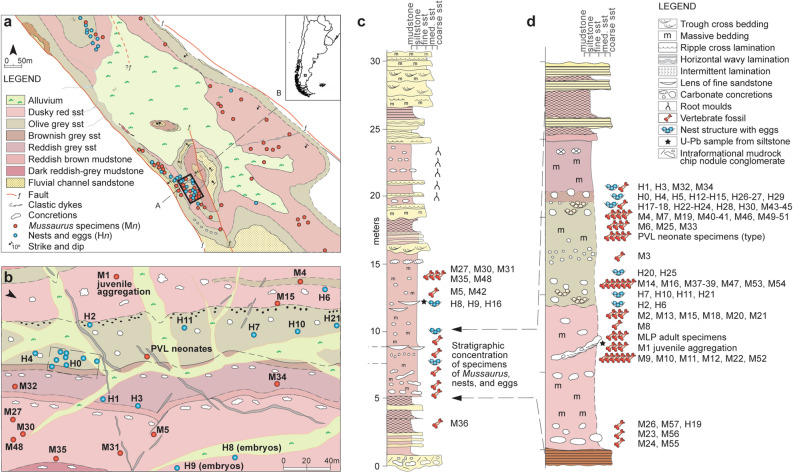
Locality map and stratigraphic section of the Laguna Colorada type locality. (**a**) general map of fossil findings at the locality (red dots represent skeletal remains of *M. patagonicus* and blue dots represents eggs or nests, cross section A–B is provided in the Supplementary Information); (**b**) detailed map of area with high fossil density (including associated juveniles, neonates, and nests), location of this area is indicated with a black rectangle in general map. Refer to Supplementary Information Fig. 2 showing the structural dip of strata approximately 15 degrees southwest. Arrow in maps indicates north; (**c**) general stratigraphic section of the type locality showing the position of skeletal remains and eggs/nests of *M. patagonicus*; (**d**) detailed stratigraphic section of the 3 m-thick interval with the highest concentration of *Mussaurus* skeletons and eggs.

**Figure 2:**
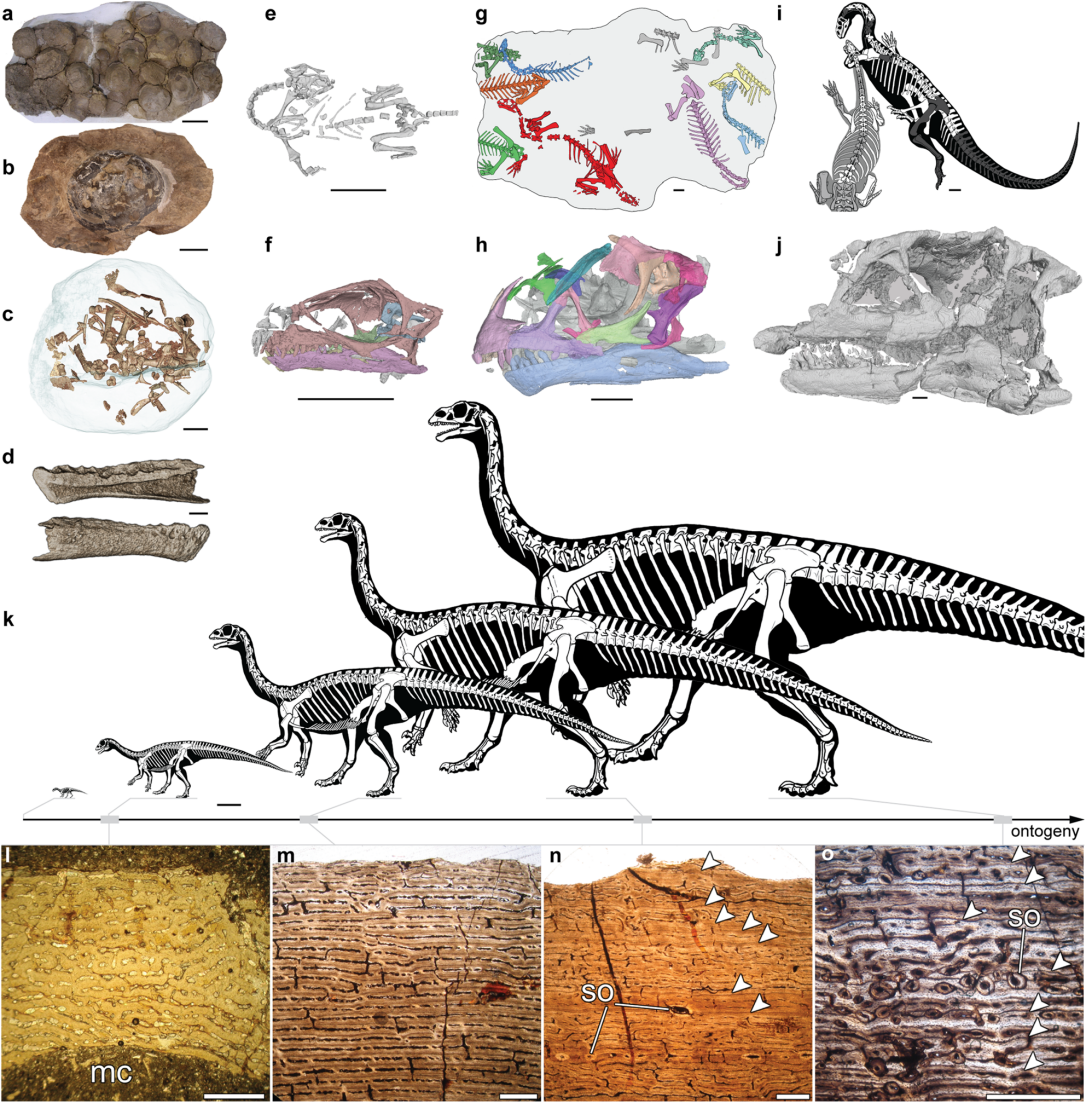
Specimens of *Mussaurus patagonicus* collected from the Laguna Colorada Formation. (**a**) nest with eggs MPM-PV 1887; (**b**), isolated egg MPM-PV 1875; (**c**) 3D reconstruction of embryo within egg MPM-PV 1879; (**d**), right dentary of embryonic remains MPM-PV 1879 showing autapomorphic traits of *M. patagonicus* (anterodorsal process of dentary); (**e**), 3D reconstruction of neonate^21^ MACN-PV 4111; (**f**) 3D reconstruction of skull anatomy of neonate MACN-PV 4111; (**g**), associated skeletons of one-year old juveniles MPM-PV 1813; (**h**), 3D reconstruction of juvenile MPM-PV 1813/4; (**i**), associated skeletons of two adult individuals MPM-PV 1868 and MPM-PV 1869; (**j**), skull of adult individual MPM-PV 1868; (**k**), Skeletal reconstruction of the different growth stages of *M. patagonicus*; (**l–o**), cortical bone histology of four different sized specimens of *M. patagonicus*. (**l**), MPM-PV 1813/10. (**m**) MPM-PV 1836. (**n**) MPM-PV 1838. **o**, MLP 60-III-20–22. Arrowheads indicate the position of lines of arrested growth. Scale bars equal 20 cm (**i**, **k**), 5 cm (**a**, **e**, **g**) , 2 cm (**b**, **f**, **h**, **j)**, 5 mm (**c**)**,** 1 mm (**d**)**,** 0.5 mm (**l**–**o**). Drawings of silhouettes (by J. Gonzalez) represent the size of the different specimens representing the various ontogenetic stages of *M. patagonicus*. Abbreviations: mc: medullary cavity; so: secondary osteons.

The Laguna Colorada Formation^14^ of southern Patagonia (Santa Cruz Province, Argentina) contains an exceptional fossil locality (Fig. 1) that provides new information about the social behaviour of early sauropodomorph dinosaurs. The sauropodomorph *Mussaurus patagonicus* was originally described^15^ from here based on several well-preserved post-hatchling specimens (Fig. 2) associated with two partially preserved eggs. Later the anatomy of juvenile skulls was described^16^, and more recently five incomplete adult specimens have been described and identified as *M*. *patagonicus*^17^.

Our recent expeditions to the type locality have yielded 69 new *M. patagonicus* specimens (in addition to the 11 individuals discovered decades ago^15,17^). The new specimens comprise skeletons of six different ontogenetic stages ranging from embryos to adult individuals. In addition, new findings at this site include over 100 eggs in various degrees of association, some of which have preserved embryonic material showing autapomorphic traits of *Mussaurus patagonicus* (Fig. 2a–c; see Supplementary Information). All sauropodomorph remains found at this locality have either preserved autapomorphic traits of *M. patagonicus* or bear anatomical traits that are indistinguishable from *M. patagonicus*, supporting the monospecificity of this unique assemblage.

Covering an area of approximately 1 km^2^, this rich fossil occurrence is in the type locality of the Laguna Colorada Formation (Fig. 1a–b), which is a 170 m-thick succession of fluvio-lacustrine sediments deposited in a post-rift thermally induced sag basin during the Early Jurassic. All the eggs and specimens of *Mussaurus patagonicus* were recovered from three distinct horizons within a restricted 3.0 m-thick interval of pedogenically modified massive reddish-brown siltstone in the middle of the Laguna Colorada Formation (Fig. 1c–d). The fossils are encrusted in brown-weathering calcareous siltstone similar to the numerous oblate to spherically-shaped calcareous nodules that occur in the same horizons. The latter are interpreted as palustrine carbonate precipitated in massive loessic silts accumulated around a floodplain pond under a seasonally warm climate (see Supplementary Information Fig. 3). The Laguna Colorada Formation has long been regarded as Late Triassic (Norian) in age^14,15^, mainly based on occurrences of the *Dicroidium* paleoflora found in the vicinity of the vertebrate-bearing fossil horizons. However, we present here U–Pb geochronology (CA-ID-TIMS method) from tuffaceous siltstones intercalated with the vertebrate-bearing interval that yielded two overlapping dates of 192.78 ± 0.14 Ma and 192.74 ± 0.14 Ma (see Supplementary Information). These dates are younger than previously thought and now assign an Early Jurassic (Sinemurian^8^) maximum age for the *Mussaurus* bearing sediments.

**Figure 3:**
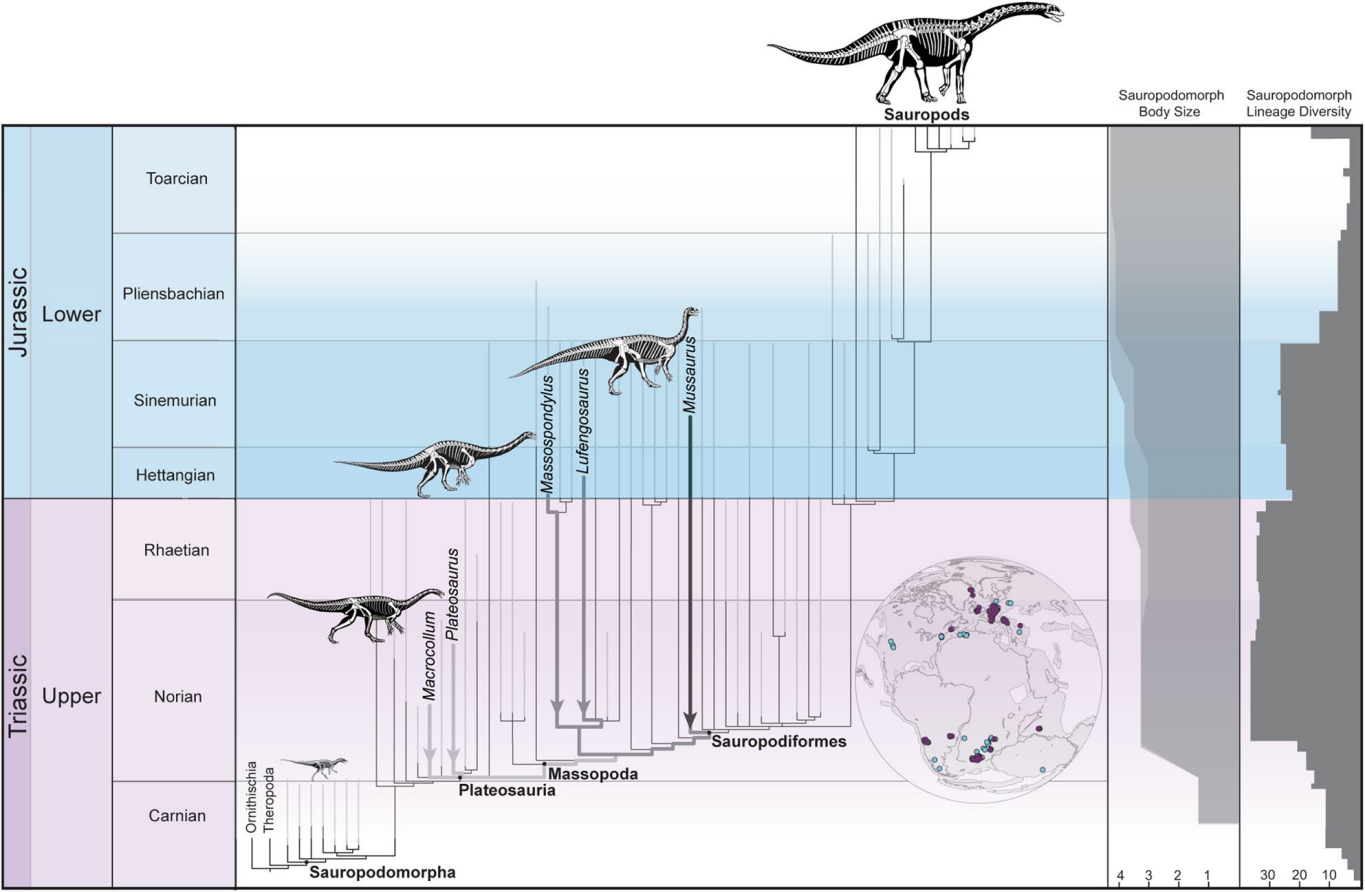
Phylogenetic tree of Sauropodomorpha calibrated against geological time. Thin grey lines on branches represent age uncertainty for terminal taxa. Thick arrows and branches on phylogenetic branches point to the origin of lineages from which behavioural data is discussed in the text. Palaeogeographic reconstruction shows the distribution of Late Triassic (purple) and Early Jurassic (blue) of skeletal remains of sauropodomorph dinosaurs (based on fossilworks.org). Body size graph represents the ancestral reconstruction of the log_10_BodyMass from the base of Sauropodomorpha to the terminal lineages of sauropods (dark shading represents the minimal values of ancestral reconstruction and light shading the maximal values of ancestral reconstruction). Linage diversity shows histogram if number of lineages inferred for each period of time. See supplementary information for details on phylogenetic analysis, optimization of body mass, and phylogenetic diversity of Sauropodomorpha. Drawings of silhouettes by J. González. Palaeographic map generated with Gplates^43^ using a Jurassic global palaeogeographic model.

Most of the eggs were found in-situ as interpreted nests containing clutches of 8 to 30 eggs (Fig. 2a). The spacing of the nests suggests a common breeding ground rather than a closely-spaced reproductive colony (Fig. 1b). Field observations and X-ray computed tomography imaging of five complete nests confirms that eggs were arranged in two or three layers (Fig. 2a) within elongate depressions or trenches with an irregular bottom profile. These depressions appear to have been purposely excavated into semi-consolidated loess and as such they qualify as nest structures.

The type specimens of *Mussaurus*^15^ originally discovered at this locality consist of eight closely-associated and notably small individuals (estimated body mass = 0.07 kg using volumetric^18^ and scaling Eqs. ^19^; see Supplementary Information). Their diminutive body size (Fig. 2d–e) suggests an aggregation of neonates as their body size largely exceeds that of the un-hatched eggs (and the new embryos here reported; Fig. 2c). The lack of size variation among them suggests they hatched at approximately the same time, and their estimated centre of mass distribution^18^ and ratio of humeral to femoral shaft circumference^20^ led to infer a quadrupedal stance for these neonates.

Amongst our recently collected specimens of *Mussaurus patagonicus* is a remarkable aggregation of at least eleven articulated juvenile skeletons (Fig. 2f–g; estimated body mass = 8.3–10.9 kg using volumetric^18^ and scaling Eqs. ^19^; see Supplementary Information), found approximately 50 m from the type hatchlings (Fig. 1b). Taphonomic assessment of this juvenile aggregation rules out the possibility of post-mortem transport and concentration or time-averaging of the carcasses and suggests synchronous death and burial of behaviourally aggregated individuals (i.e., intrinsic biogenic concentrations^21^) to be the most likely scenario. The skeletons are either fully-articulated or partially disassociated with some individuals intertwined and overlapping each other (Fig. 2g). Each of the bones of these skeletons is surrounded by a 0.5 cm-wide phosphatic halo (Supplementary Information Fig. 12e) which is possibly related to microbial decomposition of soft-tissue^22^, supporting the interpretation that the skeletons were buried relatively rapidly at the same time (see Supplementary Information). Histological thin-sections of the long bones and ribs from three different individuals of the juvenile aggregation show patterns suggestive of a fast growth rate (e.g., wide vascular spaces, cortex entirely formed by fibro-lamellar tissue consisting of woven-fibered matrix with large open channels; Fig. 2j) and absence of lines of arrested growth (LAGs) in their femora, tibia, humeri, and ribs^23^. These histological data combined with the fact that all the specimens are the same size (see Supplementary Information) suggests that these individuals were possibly members of a single brood^23^ and died together before reaching the first year of life (or, alternatively, they were young juveniles experiencing rapid uninterrupted growth during the first years of life). Both the estimated centre of mass distribution^18^ and the ratio of humeral to femoral shaft circumference^20^ of these juvenile specimens yielded ambiguous results regarding their possible quadrupedal or bipedal stance^18,20^.

Our new collecting also includes nine *Mussaurus* specimens that are intermediate in size between one-year old juveniles and adults. Histological features of the femoral mid-shafts of these intermediate-sized individuals allow us to recognize two distinct sub-adult ontogenetic stages. In the smaller sub-adult specimens (estimated body mass = 106.2 kg using scaling equations ^19^; see Supplementary Information) a single LAG is usually present, the vascularization is laminar to plexiform, with predominance of circumferentially oriented canals, there are small well-developed primary osteons, and the cortex is formed by fibro-lamellar bone (Fig. 2k). Larger sub-adult specimens (estimated body mass = 557 kg using scaling equations ^19^; see Supplementary Information) have over ten LAGs and/or annuli (see Supplementary Information for more detailed histological data, including a characterization of both the LAGs and the annuli). The change from fibro-lamellar to parallel-fibred bone in the mid cortex marks a decrease in the growth rate and suggests that sexual maturity has been attained (Fig. 2l). The largest known specimen of *Mussaurus patagonicus* (estimated body mass = 1504.8 kg using scaling equations ^19^; see Supplementary Information) has eight closely spaced LAGs toward the external part of the cortex, which indicates a substantial reduction in growth rate, as preserved on the lateral region of the cross section (Fig. 2m). Both the estimated centre of mass distribution^18^ and the ratio of humeral to femoral shaft circumference^20^ in adult skeletons of *Mussaurus* unequivocally indicate a bipedal mode of locomotion^18,20^, contrasting with the inferred quadrupedal stance of neonates (Fig. 2). Two articulated adult specimens of *M. patagonicus* found in stratigraphically equivalent beds at a nearby locality (see Supplementary Information) were closely associated with each other (Fig. 2h), demonstrating the likelihood of herding behaviour in adult individuals.

## Discussion

The multiple *Mussaurus* aggregations in the Early Jurassic breeding ground of the Laguna Colorada Formation are interpreted as the oldest skeletal evidence of structured age-segregated gregariousness amongst dinosaurs, pre-dating by over 40 million years reports from Late Jurassic and Cretaceous neosauropods^24–26^. Our new findings on *Mussaurus* adds important insights on the social behaviour of early sauropodomorphs.

Previous studies reported colonial nesting in the similarly-aged early sauropodomorphs *Lufengosaurus* from China^27^ and *Massospondylus* from South Africa^28^. In the case of *Massospondylus*, there also is evidence of site fidelity in its nesting habits^28^, as in *Mussaurus*. These three colonial nesting sites share certain palaeoenvironmental similarities that are worth mentioning. The eggs and nests of *Mussaurus* were found in a sequence of loessic silt, interpreted as windblown dust deposits on the margins of a playa-type arid zone lake. A similar loessic floodplain setting has been inferred for the deposits with nests of *Massospondylus* in South Africa^28^. In the case of the bonebed of embryonic remains of *Lufengosaurus*^27^ the depositional environment was also interpreted as a low-relief system with periodic ponding and disecation under semi-arid conditions^27^. The similarities in the depositional settings of these localities may suggest an environmental preference by early sauropodomorphs while establishing nesting grounds, although data from other early lineages are needed to test the generality of this hypothesis.

The co-occurrence of associated neonates, juveniles, and adults of *Mussaurus* in the same restricted stratigraphic interval and at the same locality suggests individuals maintained social cohesion throughout the different stages of their lifespan (notwithstanding possible seasonal variations, as in many modern gregarious species^24^). In particular, presence of juveniles and sub-adult specimens of *Mussaurus* at this site would not be expected if only sexually mature individuals congregated temporarily at their preferred nesting area (as in extant crocodiles^29^). Age segregation is a key component of gregarious behaviour and has been well-documented in large-bodied herbivorous mammals^30^. This is particularly common in extant animals with large body size difference between juveniles and adults, as the activity patterns and foraging habits vary significantly during ontogeny, and the synchronization of these behaviours is crucial for group cohesion^30^. The adoption of age-based social partitioning in *Mussaurus* is compatible with its remarkably large body size increase from hatchlings to adults (0.1 to 1500 kg), the need for several years to attain a subadult body size, and with the different postural habits or locomotion modes (quadrupedal, bipedal) inferred for neonates, juveniles, and adults^18^.

*Mussaurus* is deeply nested within the phylogeny of sauropodomorphs^17,31,32^, and its extensive ghost lineage extends back to the Late Triassic (black arrow in Fig. 3), originating at the large radiation of early sauropodomorphs. Calibration of phylogenetic trees with recent chronostratigraphic data^33,34^ place this evolutionary event in the mid-Norian, when sauropodomorphs became predominant in many terrestrial ecosystems and their phylogenetic diversity increased abruptly (Fig. 3).

The deep origin of the *Mussaurus* lineage suggests the appearance of structured gregarious behaviour may also extend back to the mid-Norian radiation of sauropodomorphs (at least to the node Sauropodiformes; Fig. 3). However, it is also possible that structured gregarious behaviour may have also arisen at some point during the approximately 18 million years that separate the mid-Norian radiation from the Sinemurian age of *Mussaurus*. Only new data on sauropodiforms from the Norian–Hettangian would be able to test these alternative evolutionary scenarios.

In contrast, the currently available data most parsimoniously support a Triassic origin for the colonial nesting habits of sauropodomorphs, as suggested by previous research on the nesting habits of *Massospondylus*^28^. The massospondylid lineage also originated in the Triassic^35^ (dark grey arrows in Fig. 3) and the presence of social reproduction in three early sauropodomorph dinosaurs (representing two non-related phylogenetic lineages) allows tracing the appearance of this behaviour back to their most recent common ancestor (close to the node Massopoda; Fig. 3) during the mid-Norian radiation. The deep origin (Late Triassic) of social reproduction is, at the moment, the most parsimonious interpretation because it requires a single common origin of this behaviour (maintained in sauropods). Other scenarios require two or more independent origins of social reproduction among sauropodomorphs (e.g., convergently acquired in massospondylids and sauropodiforms).

Finally, the recent discovery of three associated skeletons of the early sauropodomorph *Marcocollum* in the early Norian of Brazil^36^ (as well as the *Plateosaurus* bone-beds from the Norian of Europe) opens the possibility that the origin of some level of gregariousness may extend back to the very base of the Norian radiation (node Plateosauria; light grey arrow in Fig. 3). Further taphonomic information on these assemblages, however, are needed to robustly infer the behavioural implications for early plateosaurian sauropodomorphs, which can be critical for timing the origin of gregariousness in Sauropodomorpha between the early and the mid Norian.

The possible origin of gregariousness in the Norian (in Sauropodiformes, Massopoda, or even Plateosauria) coincides both temporally and phylogenetically with the appearance of evolutionary novelties that have traditionally been linked to the sauropodomorph’s early success as herbivores ^3,4,9,11,37^. These include multiple anatomical changes in the dentition, skull, and postcranium^3^ (e.g., neck elongation, reduction of skull size, ventral offset of craniomandibular articulation, overlapping leaf-shaped teeth with coarse obliquely oriented denticles). However, the most conspicuous change in the early evolution of Sauropodomorpha was the abrupt increase in adult body size^5^ of over two orders of magnitude (Fig. 3), achieved through the development of accelerated growth rates^12,13^. Such a drastic change in body size likely affected multiple aspects of sauropodomorph paleobiology and behaviour, including increased energy requirements and likely larger home ranges and daily foraging distances (both of which are correlated with body size in multiple living species^38,39^). Our findings provide important data to temporally link the origin of gregariousness in herbivorous sauropodomorphs to their evolutionary increase in body size. Social behaviour may represent a previously unrecognised factor that positively influenced the evolutionary path of this clade under a correlated progression model^3^ (in addition to other anatomical and physiological traits that characterize sauropodomorph body plan).

A key and basic aspect of gregariousness is the synchronization of behaviour and the seasonal environments in which early sauropodomorphs are recorded during the Late Triassic–Early Jurassic (such as the one reported here for *Mussaurus*) may have influenced the evolution of this features. Early sauropodomorphs are mostly recorded at mid-to-high palaeolatitudes^40^ during this time (Fig. 3). Although data on vertebrate assemblages from low paleolatitudes is still scarce, current evidence from well-sampled sequences deposited at low paleolatitudes in the Late Triassic of North America indicates dinosaurs were rare components of the vertebrate fauna and large-bodied sauropodomorphs were absent^4,40^. The currently known distribution of early sauropodomorphs show they were predominant and ecologically successful in seasonal environments at mid-to-high palaeolatitudes, as indicated by various paleoclimatic models^34,41,42^. Environmental seasonality and the high energetic requirements of large-bodied early sauropodomorphs probably implied long foraging distances (at least during certain periods). This combination of factors may have favoured the synchronization of behaviour in gregarious sauropodomorphs that required several years to attain subadult body size^12^.

We postulate the exceptional case of *Mussaurus*, in which our data show herd behaviour and age-segregation structure, indicates sociality may have influenced the early success of the first global radiation of large-bodied herbivorous dinosaurs.

## Methods

### U–Pb geochronology

In order to determine the age of the fossil-bearing strata independently, zircons were extracted from samples of tuffaceous siltstone and analysed by the chemical abrasion isotope dilution thermal ionization mass spectrometry (CA-ID-TIMS) method. Geochronology samples weighed approximately 5 kg. After manual sledging and pulverization in a Shatterbox^®^, samples were water-washed to remove their fine (< 10 µm) particles. A zircon-rich mineral concentrate was obtained using standard magnetic as well as high-density liquid separation. Final zircon selection was carried out by hand picking under a binocular microscope based on crystal morphology. Preference in zircon selection was given to prismatic zircon with glass (melt) inclusions parallel to their crystallographic “c” axis and no detectable evidence of abrasion or rounding^44^.

Zircon U–Pb analyses at the MIT Isotope Laboratory followed the same analytical procedures used previously^45^. Selected zircon grains were pre-treated by a chemical abrasion (CA-TIMS) method^46^ to mitigate the effects of radiation-induced Pb loss. The chemical abrasion schedule consisted of thermal annealing of zircon at 900 °C for 60 h, followed by partial dissolution in 27 M HF at 210 °C for 12 h. Pre-treated zircons were fluxed successively in dilute HNO_3_ and 6 M HCl and rinsed in between with Millipore® water to remove the leachates. The grains were then spiked with the EARTHTIME ET535 mixed ^205^Pb–^233^U–^235^U tracer^47,48^ before complete dissolution at 210 °C for 48 h. Dissolved Pb and U were purified using ion-exchange column chemistry, loaded together onto outgassed Re filaments, and their isotopic ratios were measured on a VG SECTOR 54 multi-collector thermal ionization mass spectrometer equipped with a Daly ion-counting system. Isotopic data reduction, date calculation and propagation of uncertainties was carried out using computer applications Triploi and ET_redux^49^ that utilize the algorithms of McLean and collaborators^50^. Complete U–Pb data appear in the Supplementary Table 1.

The sample ages are derived from the weighed mean ^206^Pb/^238^U date of the youngest population of analyses from each sample after excluding demonstrably older detrital or xenocrystic zircons, and interpreted as the maximum age of deposition for the corresponding strata. No young analysis was excluded. Uncertainties in the weighed mean ^206^Pb/^238^U dates are reported at 95% confidence level and follow the notation ± *X*/*Y*/*Z* Ma, where *X* is the internal (analytical) uncertainty in the absence of all external errors, *Y* incorporates *X* and the U–Pb tracer calibration error, and *Z* includes the latter as well as the U decay constant errors^51^. Complete uncertainties (*Z*) must be taken into account for comparison between age data from different isotopic chronometers (e.g., U–Pb versus ^40^Ar/^39^Ar), whereas for comparison between U–Pb ID-TIMS dates obtained using the same isotopic tracer the external errors can all be ignored.

### Phylogenetic study

The phylogenetic analysis was based on an expansion of a previously published data matrix focused on early sauropodomorphs^32^. Taxon sampling was expanded adding recently described species from the Late Triassic and Early Jurassic. A total of 76 taxa were included in the data matrix. Character compilation of these sources resulted in a total of 419 characters (see Supplementary Information). An equally weighted parsimony analysis was conducted in TNT 1.5^52^. A heuristic search using new technologies algorithms was applied until 100 hits to minimum length was reached. A subsequent search was conducted performing a round of TBR branch swapping on the most parsimonious trees (MPTs).

### Histological analysis

Histological samples of the femora were obtained to assess the ontogenetic stage of different individuals. The specimens were prepared for thin sectioning based on the methodology described by Chinsamy and Raath^53^ and samples for thin sectioning were obtained from the mid-shaft of the femora, below the fourth trochanter. The slices were studied and photographed using petrographic polarizing microscope (Nikon E400).

### Synchrotron X-ray tomography

The egg MPM-PV 1879, the neonate MACN-PV 4111 and two skulls of juvenile, MPM-PV 1813/2 and MPM-PV 1813/4, were scanned at the ID19 beamline of the ESRF using propagation phase contrast synchrotron X-ray micro-Computed Tomography (PPC-SRµCT). While the general configuration for PPC-SRµCT was similar for all scans, beam and acquisition parameters were adjusted for each specimen (see Supplementary Information). All scans were performed using filtered white beam from a wiggler W150B (wiggler gap and filters adjusted per specimen; see Supplementary Table 3). Images were recorded using an indirect detector consisting of a scintillator (see Supplementary Table 3), a set of optical camera lenses and a PCO.edge 5.5 sCMOS camera with camera link (PCO, Kelheim, Germany). Pixel size was measured on radiograph, measuring the shift of an object on the sample stage, moved by the most reliable translation motor. To benefit from the phase aspect of PPC-SRµCT, the sample to detector distance was adjusted considering the energy and the pixel size. As the specimens were larger than the horizontal field of view (hFOV), the centre of rotation was shifted to increase the reconstructed hFOV. Acquisitions were performed recording images while the sample was continuously rotated over 360°. To cover the vertical extent of the specimens, several acquisitions were necessary, shifting the specimen vertically in between acquisitions. The number of projections was generally of 6000 projections except for the acquisition of the full skeleton of MACN-PV 4111 specimen. For the latter, only 4000 projections were recorded per acquisition as a binning 2 × 2 was applied on the radiograph before reconstruction. Finally, to increase the signal-to-noise ratio, each projection was the result of several frames being accumulated and finally recorded as 32-bit images. Hence, the exposure time in Supplementary Table 3 is the total integrated time, considering the accumulation of several frames.

As the beam was stable (i.e., no monochromator drift), images without the sample (flatfield) and images without the beam recording the noise of the detector (darkfield) were recorded only once for each configuration used. It consisted for each set of configurations of 201 images recorded for the flatfield and 200 for the darkfield (final images calculated with a median and an average respectively). The flatfield image is then normalised based on the synchrotron current at the beginning and the end of each acquisition.

Radiograph were stitched on the vertical axis prior to reconstruction^54^. The tomographic reconstruction was done using PyHST2 and the single distance phase retrieval approach^55,56^. Processing following the tomographic reconstruction included: conversion of the 32-bit data to 16-bit, removing 0.002% for both maximum and minimum values; ring correction on slices^57^; cropping of the data.

### Body mass estimates

Body mass estimates of the different specimens of *Mussaurus patagonicus* were calculated based on the measurements of femoral circumference using the scaling equation for bipedal non-avian dinosaurs as implemented in the MASSTIMATE 1.3 package for R^18^, using the quadratic equation implemented in the cQE function with the options equation = raw, cor = 2, and quadratic = TRUE.

## Supplementary Information

Supplementary Information 1.

Supplementary Information 2.

## Data Availability

All data related to the geochronological study, specimen list, and phylogenetic analysis presented in this paper is detailed in the Supplementary Information. All materials are available from the corresponding authors upon request. Specimens and GPS coordinates are deposited at the Museo Padre Molina (MPM), following the legal requirements of the Santa Cruz Province (Argentina).
