# Supplementary material for: Earliest evidence of herd-living and age segregation amongst dinosaurs
Source: Sci Rep. 2021 Oct 21;11:20023. doi: 10.1038/s41598-021-99176-1 (PMC8531321; doi:10.1038/s41598-021-99176-1)
Supplement: Supplementary file 2 — Supplementary Information 2. [file 41598_2021_99176_MOESM2_ESM.docx]

**Supplementary Information**

**Earliest evidence of herd-living and age segregation amongst dinosaurs**

Diego Pol^1^, Adriana C. Mancuso^2^, Roger M. H. Smith^3^, Claudia A. Marsicano^4^, Jahandar Ramezani^5^, Ignacio A. Cerda^6^, Alejandro Otero^7^ & Vincent Fernandez^8^

**Summary:**

1. Age of the *Mussaurus*-bearing levels of the Laguna Colorada Formation
2. Supplementary geological figures
3. Supplementary anatomical figures
4. Table of *Mussaurus* *patagonicus* specimens
5. Taphonomic notes on selected specimens
6. Preservation of embryonic remains of *Mussaurus patagonicus*
7. *Synchrotron X-ray micro-Computed Tomography*
8. Body mass estimates of *Mussaurus* *patagonicus* specimens
9. Histological analysis of *Mussaurus patagonicus*
10. Phylogenetic analysis of *Mussaurus patagonicus*
    1. Analytical procedures
    2. Strict consensus
    3. Reduced consensus
    4. Calibration against geological time
    5. List of synapomorphies
    6. Character list
    7. Data matrix
11. References

**1- Age of the Mussaurus-bearing levels of the Laguna Colorada Formation**

*Sample El Tranquilo* – This sample is a tuffaceous siltstone which contained the assemblage of juvenile *Mussaurus* specimens MPM 1813 (field code M1; see Figs. 1, 2g–h). Three youngest U-Pb zircon analyses from this sample form a statistically coherent cluster with a weighted mean ^206^Pb/^238^U date of 192.78 ± 0.14/0.17/0.27 Ma and a mean squared weighted deviation (MSWD) of 2.2. Three older analyses range in ^206^Pb/^238^U date from 241.47 ± 0.22 Ma to 482.31 ± 0.49 Ma and represent recycled (detrital) zircon.

*Sample H8* – This tuffaceous siltstone sample was collected from the nest site catalogued under field number H8 (see Fig. 1 and Supplementary Table 1). Five analysed zircons from this sample range in ^206^Pb/^238^U dates from 192.62 ± 0.22 Ma to 194.0 ± 1.0 Ma, indicating a scatter that exceeds the analytical uncertainty. However, the youngest three analyses yield a a weighted mean ^206^Pb/^238^U date of 192.74 ± 0.14/0.17/0.27 Ma (MSWD = 1.4).

The above two weighted mean dates are practically identical within uncertainty and place direct age constraints on the associated fossil assemblages. They suggest that the (maximum) age of deposition of the fossil-bearing strata of the Laguna Colorada Formation coincided with the Sinemurian Stage of the Early Jurassic based on the present calibration of the geologic time scale (Hesselbo et al., 2020; Cohen et al., 2013, updated).


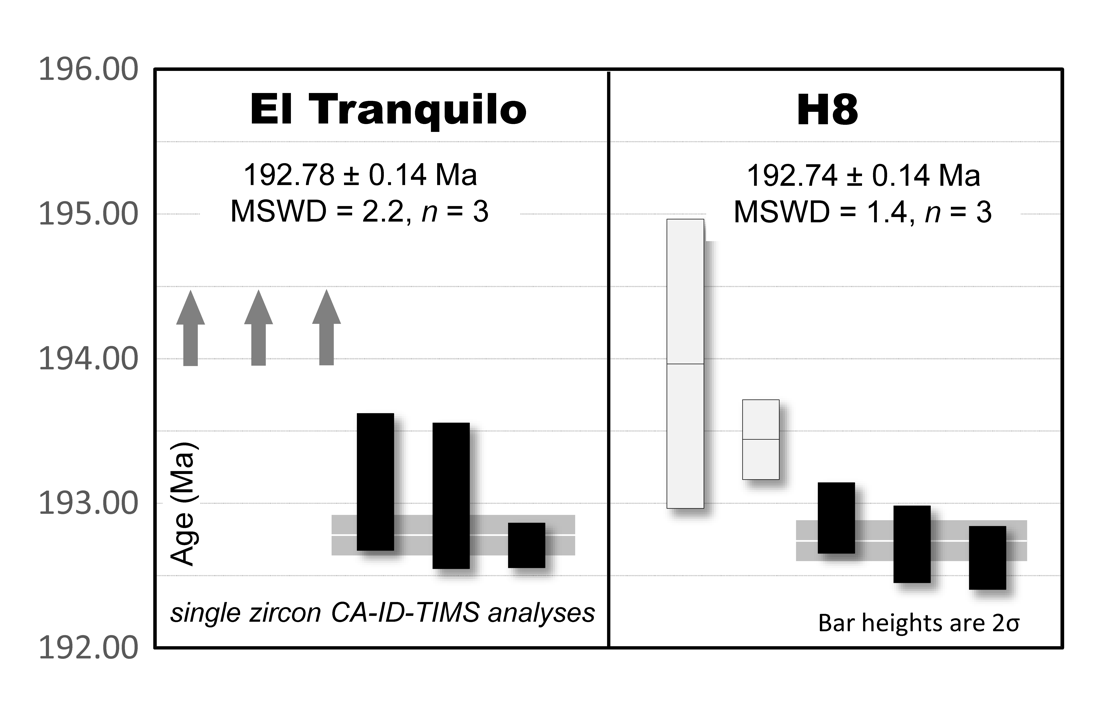


**Supplementary Figure 1.** Date distribution plot of analysed zircons from the tuffaceous rocks of the Laguna Colorada Formation. Black bars represent the 2σ analytical uncertainty of individual zircon analyses – open bars are older zircons excluded from age calculation. Horizontal line/shaded band signify the weighted mean /95% confidence interval of the ^206^Pb/^238^U date. Older detrital zircons falling outside the plot area are shown by arrows.

**Supplementary Table 1.** U-Pb isotopic data for analysed zircons from tuffaceous rocks of the Laguna Colorada Formation, Santa Cruz, Argentina.


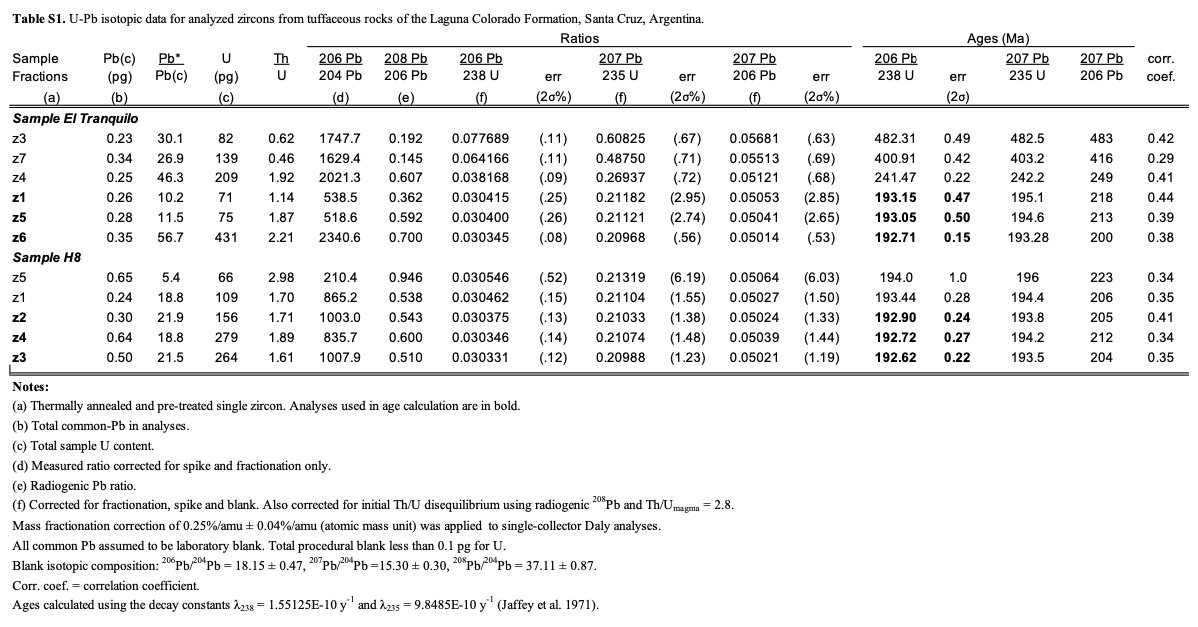


**2- Supplementary geological figures**

The Triassic–Jurassic, non-marine, sedimentary rocks of El Tranquilo Group were deposited in narrow, fault-bounded, small and isolate extensional basin and were commonly associated with volcanic and volcaniclastic deposits an elongate (Uliana and Biddle, 1987). The El Tranquilo Group consists of two depositional sequences, Cañadón Largo and Laguna Colorada Formations. The Triassic–Jurassic sequence unconformably overlies basement and the Permian Tres Cerros Group, and is, in turn, discordantly overlain by the Lower Jurassic Roca Blanca Formation and the Middle Jurassic Bahía Laura Group (Jalfin and Herbst, 1995).


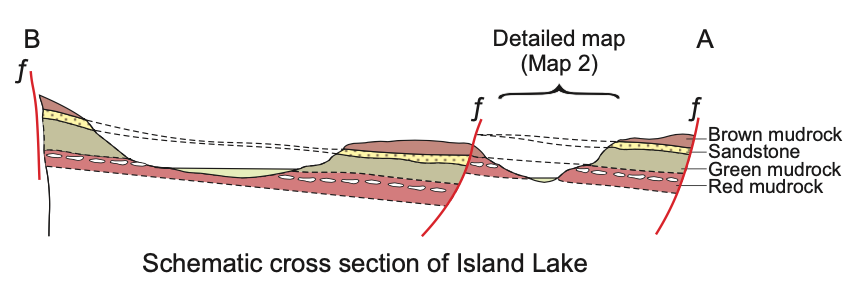


**Supplementary Figure 2.** Schematic cross-section of *Mussaurus* type locality within the type locality of the Laguna Colorada Formation. Position of the cross section and the detailed mapping area (see Fig. 1b) are indicated in Figure 1a. f=fault


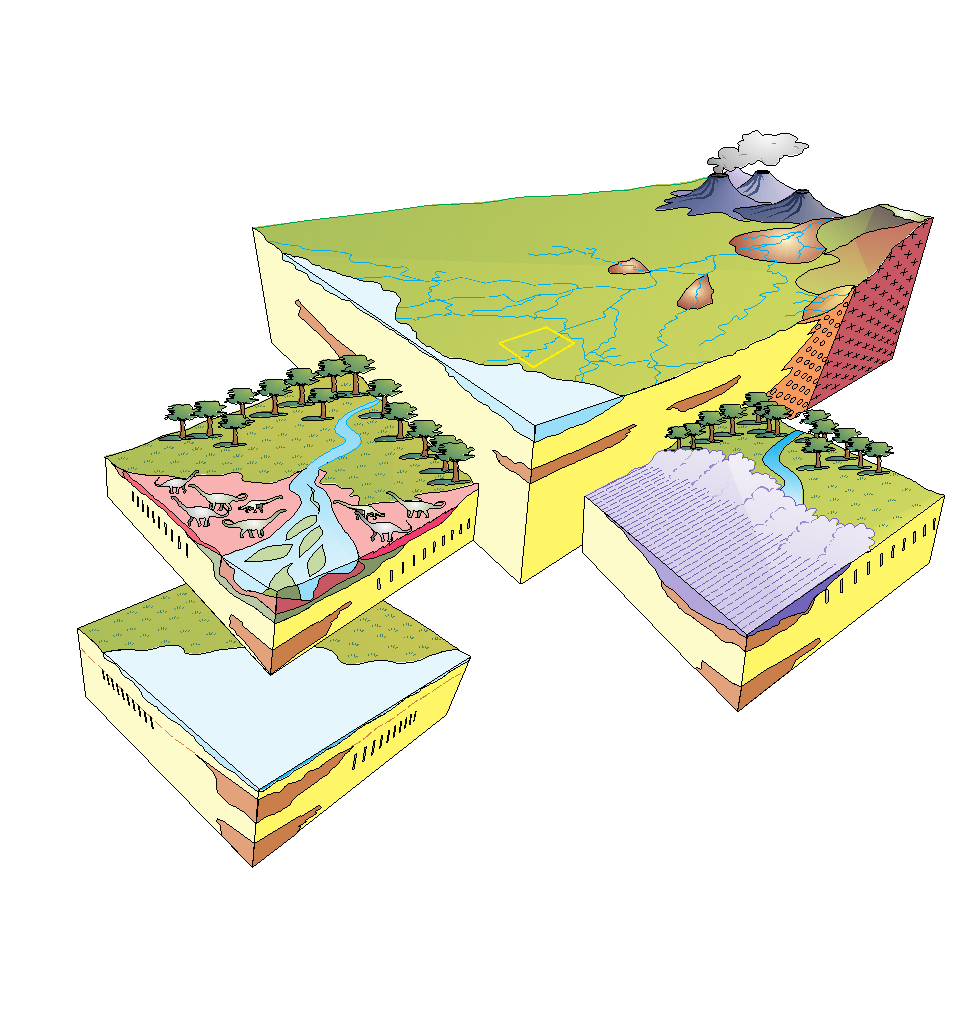


**Supplementary Figure 3.** Paleogeographic reconstruction of *Mussaurus* nesting grounds in the Early Jurassic Laguna Colorada Formation of southern Argentina. The preferred nesting areas were around the margins of floodplain lakes formed in the depocenter during the post-rift sag phase of basin development. Block diagrams 1, 2 and 3 depict the taphonomic processes that operated in the area delimited in yellow on the landscape reconstruction and led to preservation of numerous *Mussaurus* skeletons alongside their unhatched egg clusters. 1, Wind-driven dust storms deposit thick beds of silt and volcanic ash in and around a permanent floodplain lake. 2, Year after year, during the dry season, resident herds of *Mussaurus* excavate “egg chambers” into the sparsely vegetated loessic silt. 3, Some years the lake level rose too early and drowned unhatched egg clutches, other years they suffocated beneath a deep layer of ash-laden loess. The decomposition products of buried bones and eggs formed a halo in the surrounding silt which promoted seasonal precipitation of palustrine carbonate around the remains similar to that described from loessic plains of China (Li et al., 2018).

**3- Supplementary anatomical figures**

The following figures correspond to higher resolution versions of the specimen images included in text Figure 2.


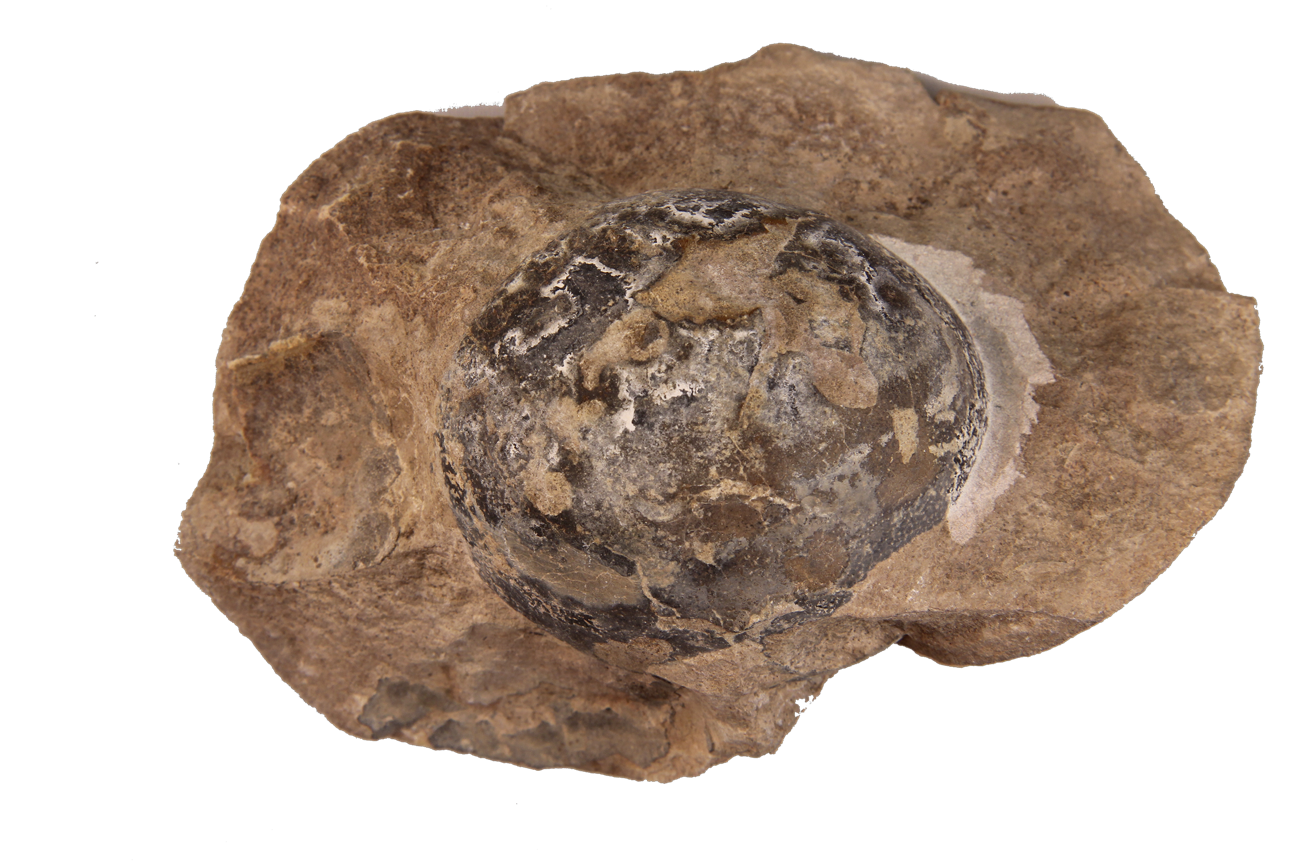


**Supplementary Figure 3.** Isolated egg MPM-PV 1875.

**
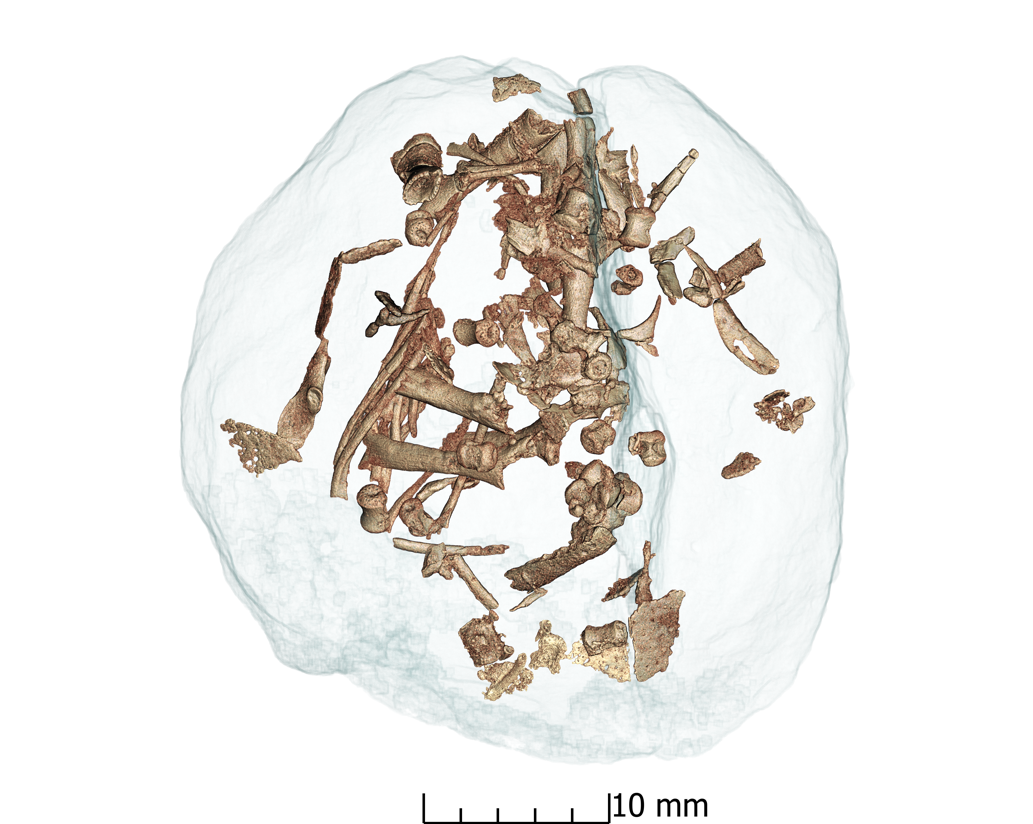
**

**Supplementary Figure 4.** 3D reconstruction of embryo within egg MPM-PV 1879.


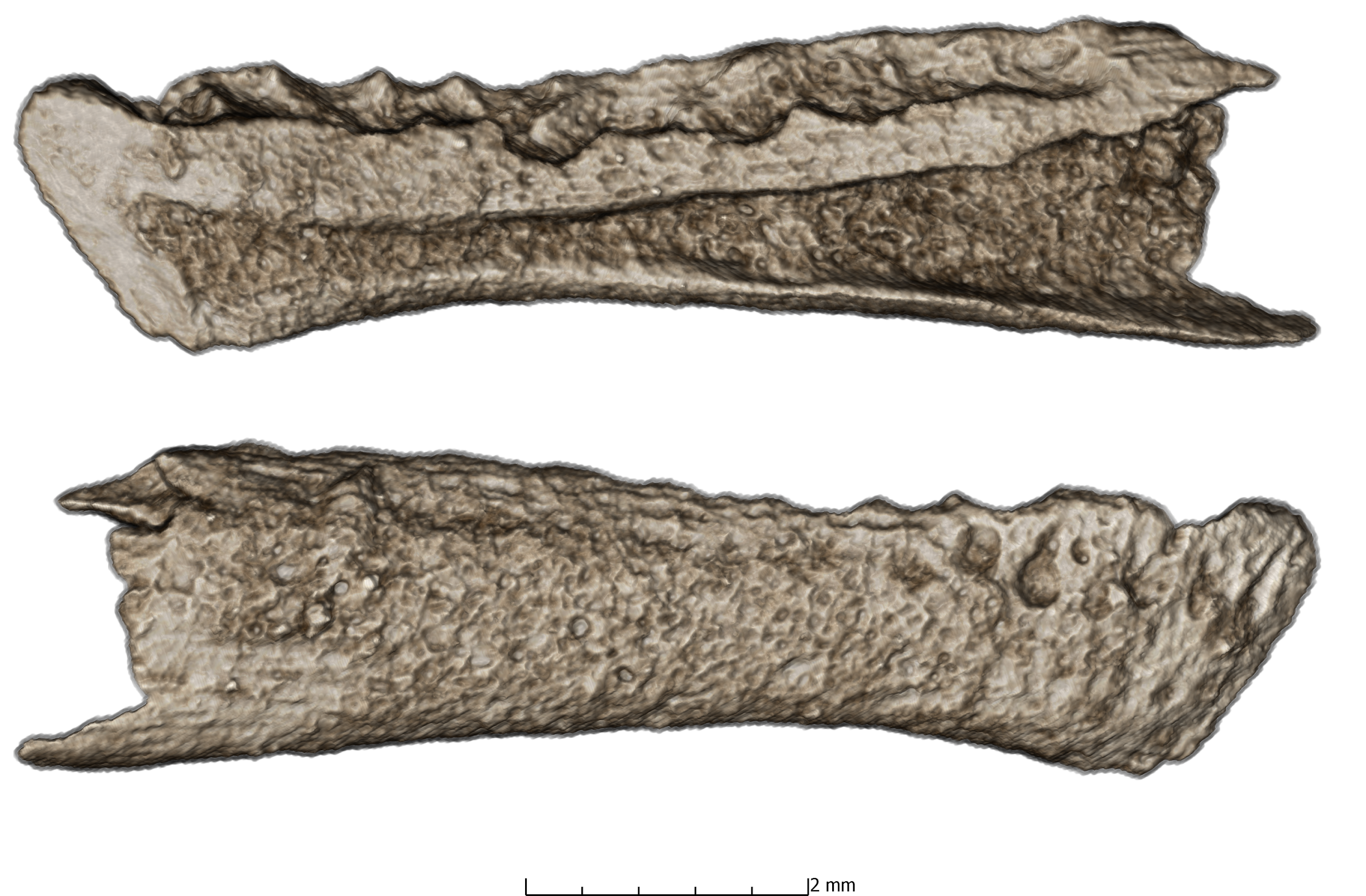


**Supplementary Figure 5.** 3D reconstruction of the right dentary from embryonic remains found in specimen MPM-PV 1879, segmented from the synchrotron scan. Medial view on top, lateral view on bottom.

**
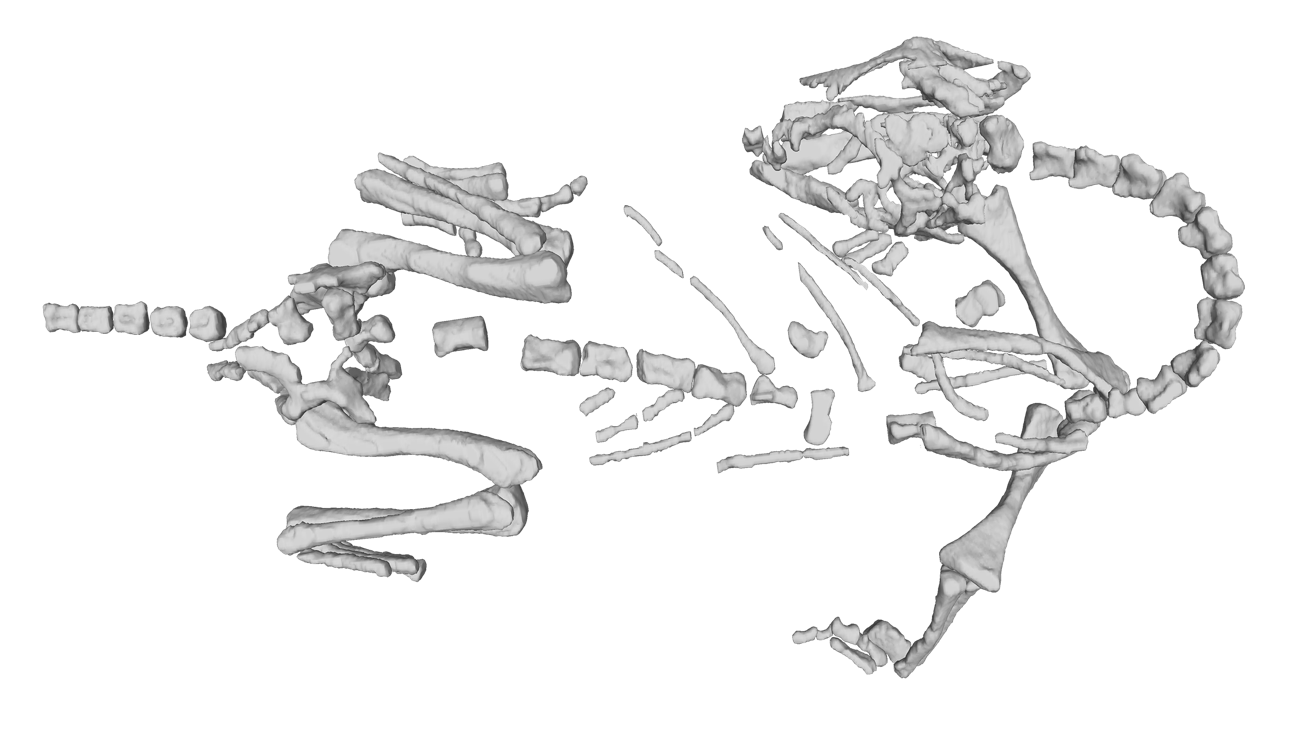
**

**Supplementary Figure 6.** 3D reconstruction of neonate MACN-PV 4111 (based on Otero et al., 2019).


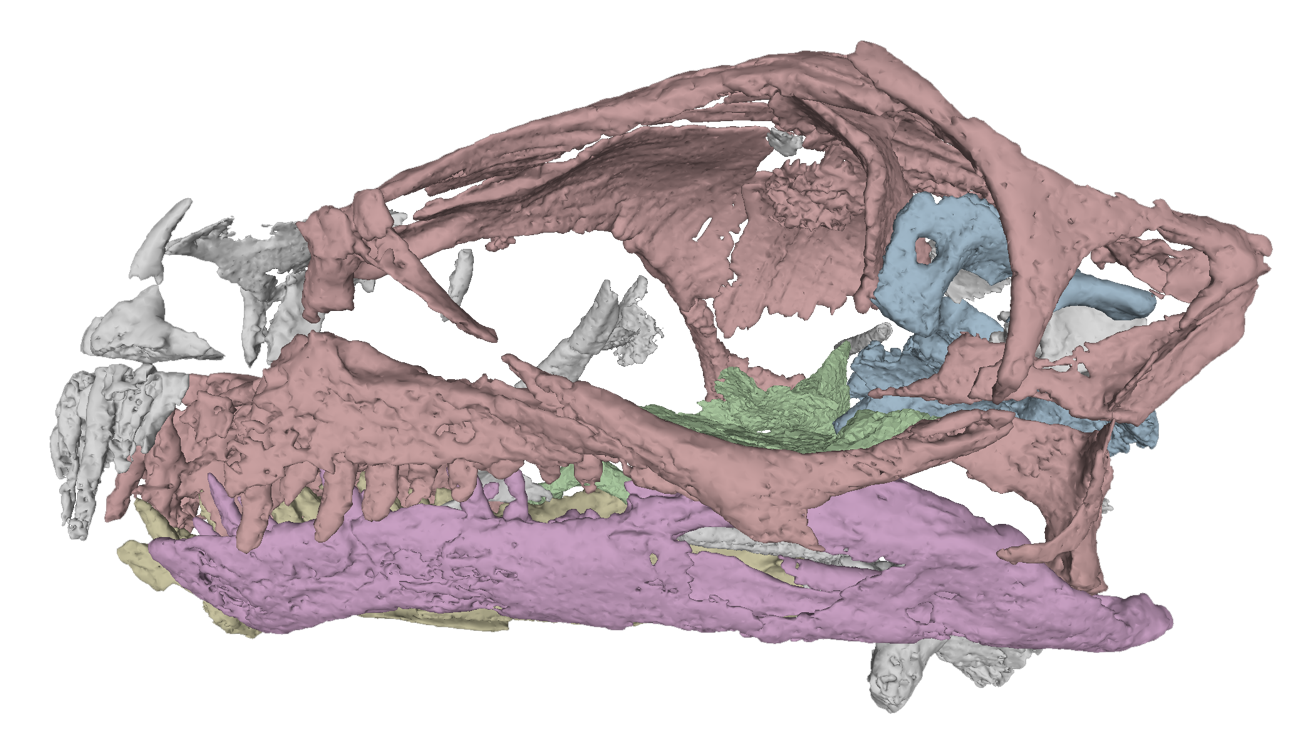


**Supplementary Figure 7.** 3D reconstruction of skull anatomy of neonate MACN-PV 4111, segmented from the synchrotron scan.


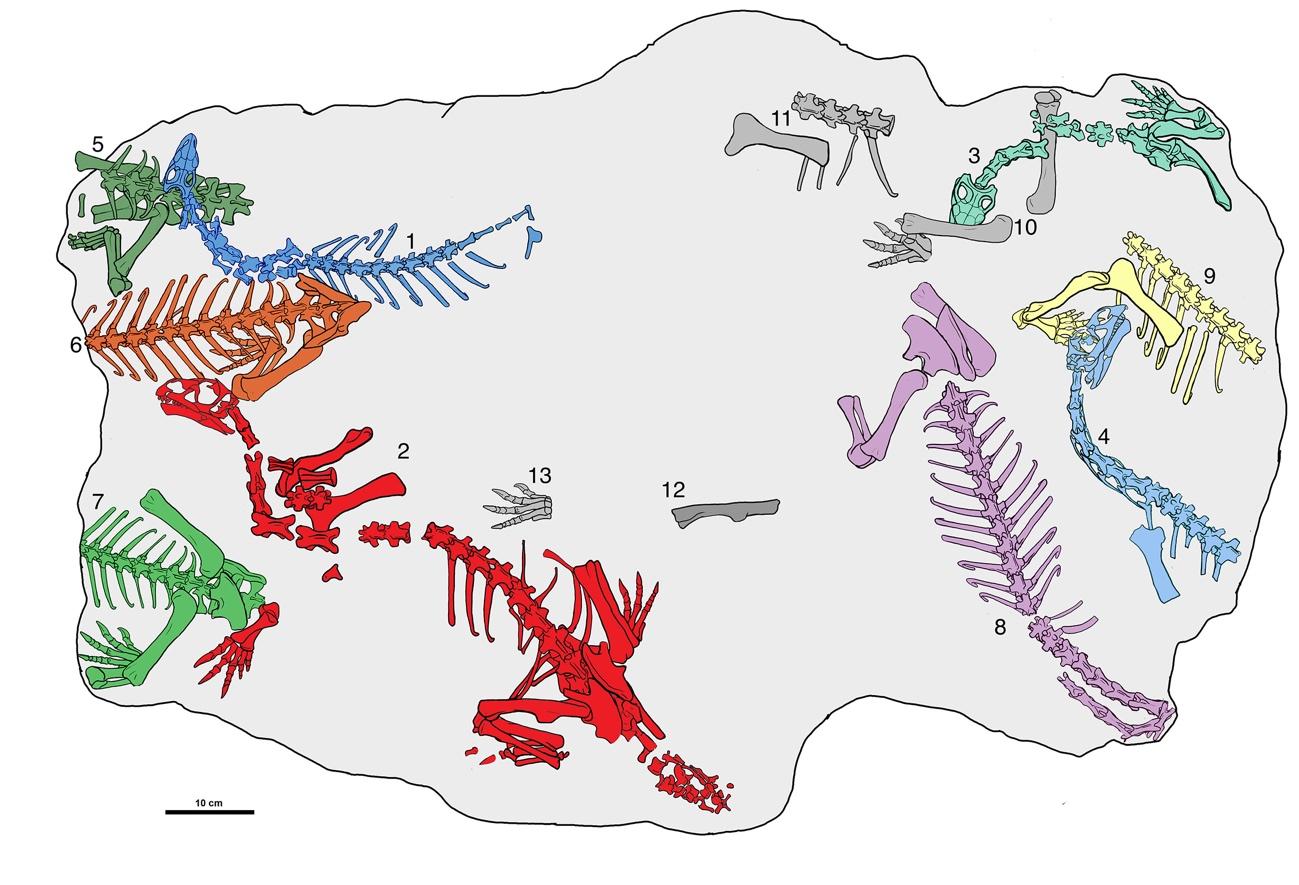


**Supplementary Figure 8.** Associated skeletons of one-year old juveniles MPM-PV 1813.


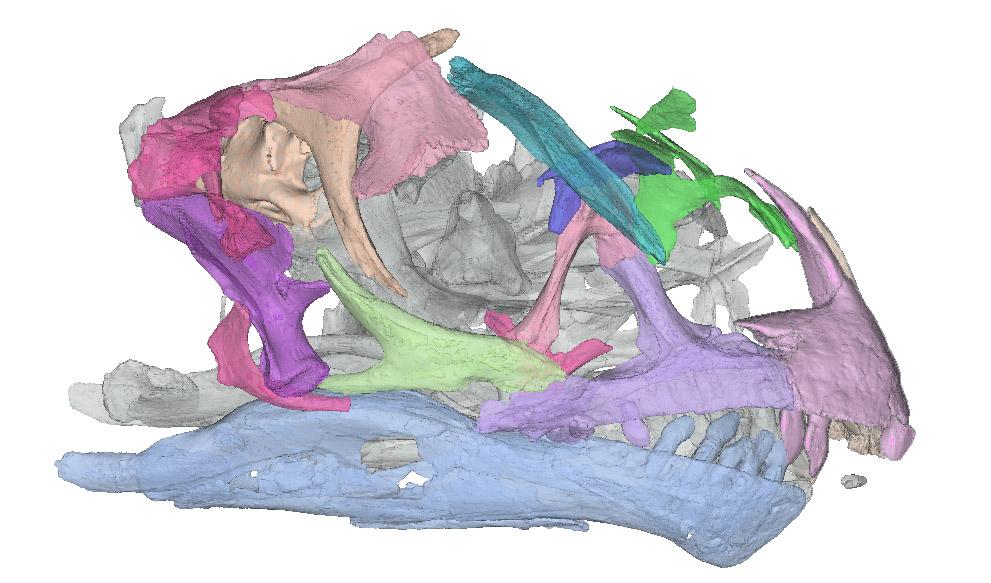


**Supplementary Figure 9.** 3D reconstruction of the skull juvenile MPM-PV 1813/4, segmented from the synchrotron scan.


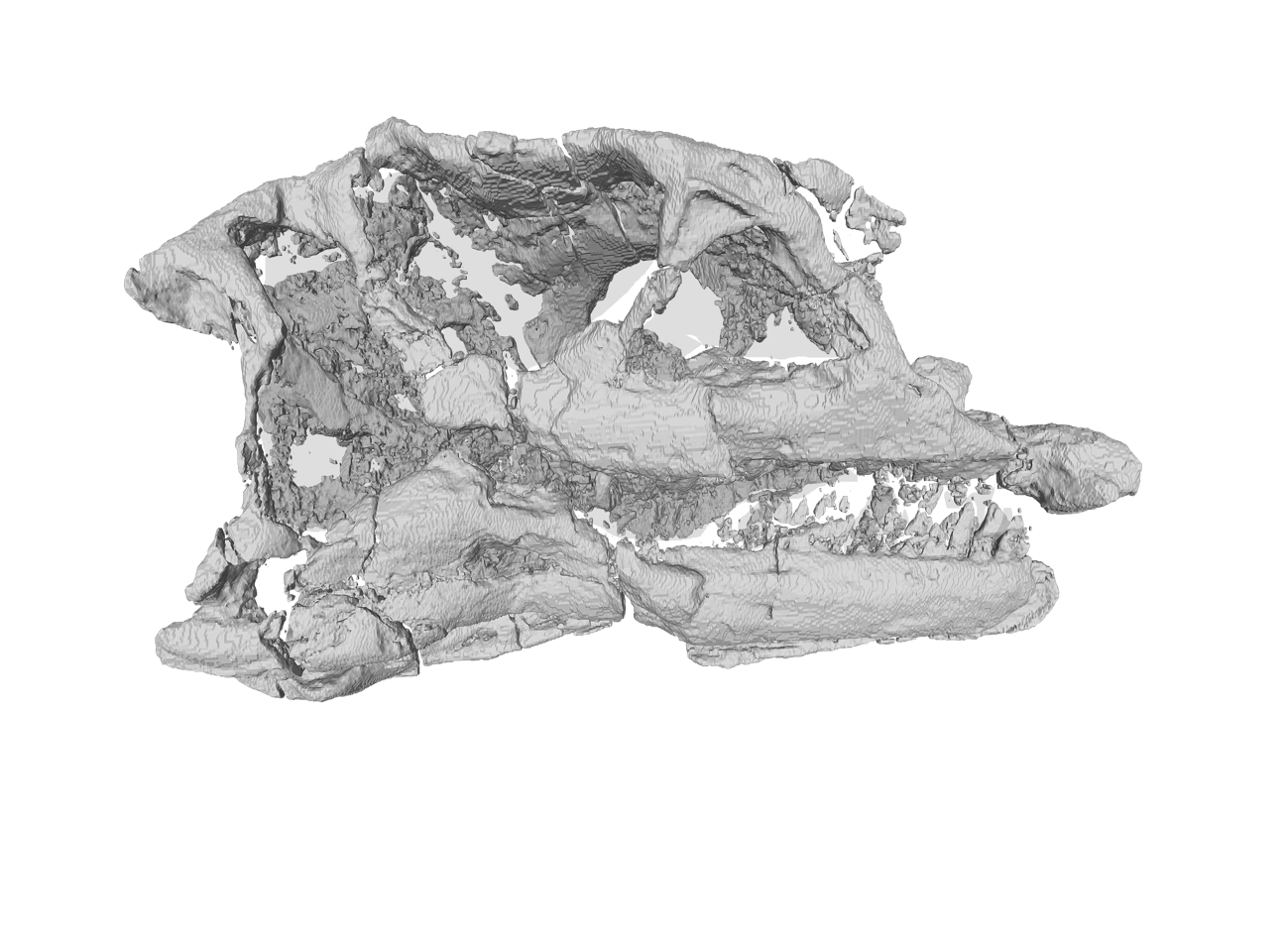


**Supplementary Figure 10.** skull of adult individual MPM-PV 1868, segmented from the medical computed tomography.

**4. Catalogue of *Mussaurus* *patagonicus* specimens**

A total of 69 sauropodomorph specimens were found in recent years at the Laguna Colorada Formation and are deposited at the Museo Padre Molina (MPM) in Santa Cruz Province, Argentina. The new specimens add to the 8 neonates (type material of *M. patagonicus*) and other 6 specimens collected by the Argentinian paleontologists José Bonaparte and Rodolfo Casamiquela in the 1960’s and 1970’s. Here we provide details on the specimens collected at the Laguna Colorada Formation, including their catalogue number, collector team, and ontogenetic stage (divided into four broad categories). A large proportion of the collected specimens are either juveniles (above 40%) or adults (above 35%).

All reported specimens (except for two articulated skeletons MPM-PV 1868, MPM-PV 1869; see taphonomy section below) were found in an area of approximately 1 km^2^ in the type locality of the Laguna Colorada Formation, as detailed in Figure 1. The two closely associated and articulated adult skeletons MPM-PV 1868 and MPM-PV 1869 were found in a secondary outcrop of the Laguna Colorada Formation located approximately 6 km NE from its type locality (referred as Di Persia lake; see Jalfin and Herbst, 1995: fig. 1).

**Supplementary Figure 11.** Percentage of the different ontogenetic stages of the specimens of *Mussaurus patagonicus* collected at the Laguna Colorada Formation.

**Supplementary Table 2.** List of specimens of *Mussaurus patagonicus* from the Laguna Colorada Formation. Cell color-coding represent the collector teams as follows: Casamiquela et al. during 1960’s (green), Bonaparte et al. during 1970’s (blue), and Pol et al. in recent years (orange). Field codes M*n* indicate skeletal elements identified as *Mussaurus patagonicus* (codes used in Figure 1). Collected specimens have an MPM catalogue number in addition to the field code, as detailed in the table. There are ten specimens identified as *Mussaurus patagonicus* that have a field code number but have not been yet collected (indicated in the table as TBC, to be collected). Field codes H*n* correspond to eggs remains (codes used in Figure 1). The H*n* codes can represent from eggshells fragments to complete nests composed by up to 30 eggs (see details in the table).

| **Collection #** | **Field #** | **Ontogenetic stage** | **Description** |
| --- | --- | --- | --- |
| MLP 61-III-20-22, 61-III-20-23, 68-II-27-1 | MLP | adults | 4 partially preserved postcranial materials collected by Casamiquela |
| PVL 4068, 4208, 4209, 4210, 4211, 4212, 4213, 5865 | PVL | neonates | 8 articulated and associated skeletons collected by Bonaparte |
| PVL 4587 | PVL | juveniles | 1 partially articulated skeleton (with fragmentary remains of a second individual) collected by Bonaparte |
| MACN-SC 3379 | MACN | adult | 1 partially preserved postcranium collected by Bonaparte |
| MPM-PV 1813 | M1 | juveniles | 11 articulated skeletons |
| TBC | M2 | subadult | articulated presacral, sacral, and caudal vertebrae, ilia |
| TBC | M3 | adult | associated postcranial remains |
| MPM-PV 1816 | M4 | juvenile | articulated presacral vertebrae |
| TBC | M5 | juvenile | semi-articulated presacral vertebrae |
| TBC | M6 | juvenile | vertebrae and distal femur, proximal tibia |
| MPM-PV 1819 | M7 | adult | associated presacral vertebrae |
| TBC | M8 | adult | associated postcranial remains |
| MPM-PV 1821 | M9 | juvenile | dorsal vertebra and caudal vertebrae, humerus, ulna, metatarsals |
| MPM-PV 1822 | M10 | adult | articulated manus |
| MPM-PV 1823 | M11 | subadult | manus and isolated elements |
| MPM-PV 1824 | M12 | juvenile | articulated vertebrae with neural spines, tibia, metatarsals |
| MPM-PV 1825 | M13 | adult | Ilium |
| MPM-PV 1826 | M14 | subadult/adult | disarticulated skull and metatarsals |
| MPM-PV 1827 | M15 | adult | Disarticulated limb elements |
| MPM-PV 1828 | M16 | neonate | <distal femur, phalanges, ulna, vertebrae, and other remains |
| MPM-PV 1829 | M17 | neonate | isolated femur |
| MPM-PV 1830 | M18 | subadult/adult | dorsal vertebra |
| TBC | M19 | adult | postcranial remains |
| MPM-PV 1832 | M20 | adult | presacral and caudal vertebrae, pelvic girdle |
| MPM-PV 1833 | M21 | adult | presacral vertebra and limb bones |
| TBC | M22 | subadult | femur and disarticulated postcranial remains |
| TBC | M23 | adult | postcranial remains |
| MPM-PV 1836 | M24 | subadult | skull elements, mandible, teeth, vertebrae, limb bones |
| MPM-PV 1837 | M25 | juvenile | metatarsals, scapula, and other postcranial remains |
| MPM-PV 1838 | M26 | subadult | ischia, femora, tibia, astragalus, manus, presacral vertebrae |
| MPM-PV 1839 | M27 | juvenile | presacral vertebra |
| MPM-PV 1840 | M30 | juvenile | presacral vertebra |
| MPM-PV 1841 | M31 | juvenile | femur and humerus (possibly of a smaller individual) |
| MPM-PV 1842 | M32 | juvenile | two anterior dorsal vertebrae |
| MPM-PV 1843 | M33 | juvenile | metatarsal V |
| MPM-PV 1844 | M34 | juvenile | articulated caudal vertebrae |
| MPM-PV 1845 | M35 | juvenile | sacral vertebrae |
| MPM-PV 1846 | M36 | subadult | skull remains and isolated postcranial elements |
| TBC | M37 | adult | postcranial remains |
| MPM-PV 1848 | M38 | juvenile | Forelimb remains with articulated manus |
| MPM-PV 1849 | M39 | adult | metatarsal IV and partial metatarsal III |
| MPM-PV 1850 | M40 | juveniles | dorsal vertebrae with articulated ribs |
| MPM-PV 1851 | M41 | juveniles | associated remains of two juvenile individuals with skull and postcranial remains |
| MPM-PV 1852 | M42 | subadult | ilium and fragmentary limb elements |
| MPM-PV 1853 | M43 | adult | three isolated teeth |
| MPM-PV 1854 | M44 | adult | femoral shaft and pedal phalanges |
| MPM-PV 1855 | M45 | juvenile | dorsal vertebrae |
| MPM-PV 1856 | M46 | juvenile | dorsal vertebrae |
| MPM-PV 1857 | M47 | adult | anterior cervical |
| TBC | M48 | adult | isolated dorsal vertebra |
| MPM-PV 1859 | M49 | adult | articulated caudal vertebrae |
| TBC | M50 | juvenile | articulated pes |
| MPM-PV 1861 | M51 | adult | articulated pubes |
| MPM-PV 1862 | M52 | adult | two caudal vertebrae |
| MPM-PV 1863 | M53 | adult | dorsal vertebra |
| MPM-PV 1864 | M54 | juvenile | partially articulated manus |
| MPM-PV 1865 | M55 | adult | maxilla (mold) |
| MPM-PV 1866 | M56 | subadult | pedal phalanges and ungual |
| MPM-PV 1867 | M57 | subadult | vertebrae and limb bones |
| MPM-PV 1868, 1869 | MdiP 1 | adults | 2 articulated skeletons associated with each other |
| MPM-PV 1870 | H0 | eggs | associated eggs (scattered on surface) |
| MPM-PV 1871 | H1 | eggs | 5 eggs on surface |
| MPM-PV 1872 | H2 | eggs | 1 egg on surface |
| MPM-PV 1873 | H3 | eggs | 3 complete eggs on surface |
| MPM-PV 1874 | H4 | eggs | 1 egg on surface |
| MPM-PV 1875 | H5 | eggs | nest with 30 eggs  3 complete eggs in concretion (in situ) |
| MPM-PV 1876 | H6 | eggs | 2 eggs |
| MPM-PV 1877 | H7 | eggs | fragments of eggshells |
| MPM-PV 1878 | H8 | eggs | nest of 21 eggs |
| MPM-PV 1879 | H9 | eggs | eggs with associated small bones |
| MPM-PV 1880 | H10 | eggs | eggs and eggshell fragments with associated small bones |
| MPM-PV 1881 | H11 | eggs | fragments of eggshells |
| MPM-PV 1882 | H12 | eggs | 2 eggs in concretion (in situ) |
| MPM-PV 1883 | H13 | eggs | fragments of two eggs and scattered eggshells associated with a caudal vertebra |
| MPM-PV 1884 | H14 | eggs | fragments of two eggs and scattered eggshells |
| MPM-PV 1885 | H15 | eggs | fragments of eggs and scattered eggshells |
| MPM-PV 1886 | H16 | eggs | 2 eggs |
| MPM-PV 1887 | H17 | eggs | nest with 30 eggs |
| MPM-PV 1888 | H18 | eggs | fragments of eggshells |
| MPM-PV 1889 | H19 | eggs | isolated egg |
| MPM-PV 1890 | H20 | eggs | isolated egg |
| MPM-PV 1891 | H21 | eggs | nest with eggs with embryonic remains |
| MPM-PV 1892 | H22 | eggs | 3 eggs in concretion |
| MPM-PV 1893 | H23 | eggs | 3 eggs on surface |
| MPM-PV 1894 | H24 | eggs | isolated egg |
| MPM-PV 1895 | H25 | eggs | fragments of eggs |
| MPM-PV 1896 | H26 | eggs | nest with at least 14 eggs |
| MPM-PV 1897 | H27 | eggs | 3 eggs in concretion |
| MPM-PV 1898 | H28 | eggs | 2 isolated eggs |
| MPM-PV 1899 | H29 | eggs | isolated egg |
| MPM-PV 1900 | H30 | eggs | nest with at least 8 eggs |

**5- Taphonomic notes on selected specimens**

***5.a. Aggregation of Mussaurus juveniles MPM-PV 1813 (field code M1)***

The associated juvenile *Mussaurus* specimens catalogued under the accession number MPM-PV 1813 were found at the margin of Laguna Colorada ephemeral lake at the same stratigraphic level as numerous other sauropodomorph dinosaurs (see M1 in Fig. 1). The host sediments are mottled grayish-red siltstones which have been interpreted as having been deposited on a floodplain associated with ephemeral ponds and paleosoils (Jalfin and Herbst, 1995). The juvenile remains are preserved in a calcareous concretion of approximately 1.5 m wide, 1 m long, and 0.15 m deep. The long axis of the concretion is aligned NE/SW and has an inclination of 15–20° crosscutting the host sediment bedding planes.

The skeletons within the concretion become deeper in a NNW trend, which is opposite to long axis trend. The orientation of the skeletons shows a general WSW trend. There are no signs of transport in the preserved skeletons.

The assemblage includes at least eleven similar-sized young individuals of *Mussaurus patagonicus* that are partially or fully articulated and most of the missing skeletal elements may be attributed to recent weathering and incomplete collecting. Some of the elements close to the top surface are disarticulated but still closely associated and show a randomized dispersal pattern that may be related to scavenging or some other pre-burial disturbance (Suppl. Fig. 12a). Some individuals are in life-like crouched position with flexed posterior limbs (Suppl. Fig. 12a) whereas others are with their anterior and posterior limbs articulated crosscutting the underlying strata (Suppl. Fig. 12b). These articulated limbs penetrate the concretion, crosscutting the strata throughout a range of 5 to 13 cm. Some specimens are intertwined with each other with the skull of one located below limbs of other individual (Suppl. Fig. 12c).

The bones show post-mortem modification as superficial cracking, fractures, corrosion and flaking of outer surface. Moreover, the bones located more superficially show evidence of more exposure than the bones located deeper in the sediments (Suppl. Fig. 12d). This is also evident in two skulls preserved on their lateral sides, in which the side facing down has the rostral and temporal elements in natural articulation and the corresponding elements of the other (top) side are slightly displaced from their natural position. The general features correspond to a 1–2 weathering state (sensu Behensmeyer, 1978) that suggest between 2 or 3 years of sub-aerial exposure. The presence of a yellow-gray halo (approximately 0.5 cm thick) around the bones (mainly in articulated elements) suggests a local reducing microenvironment created by soft tissue decomposition caused by microbial activity (Suppl. Fig. 12e). This microenvironment may exacerbate the weathering rate and corrosion (Lyman, 1994; Downing and Park, 1998). Thus, the high degree of articulation and associated reduction halo surrounding the bones is indicative of relatively rapid burial of the skeletons soon after death.

The spatial disposition of this assemblage, the relationship of skeletons and host sediment, the natural articulation and occurrence of intertwined skeletons suggest the absence of post-mortem transport. In addition, similar weathering stages (between 1 and 2) for all bones supports the absence of time-averaging for this assemblage. Thus, the sum of the above-mentioned evidence supports an intrinsic biogenic concentration for this assemblage (Rogers and Kidwell, 2007). We interpret this as a behavioural “huddling” of at least 15 juvenile *Mussaurus* (likely one year old or less; see histology section below) that were rapidly buried in their natural resting position.


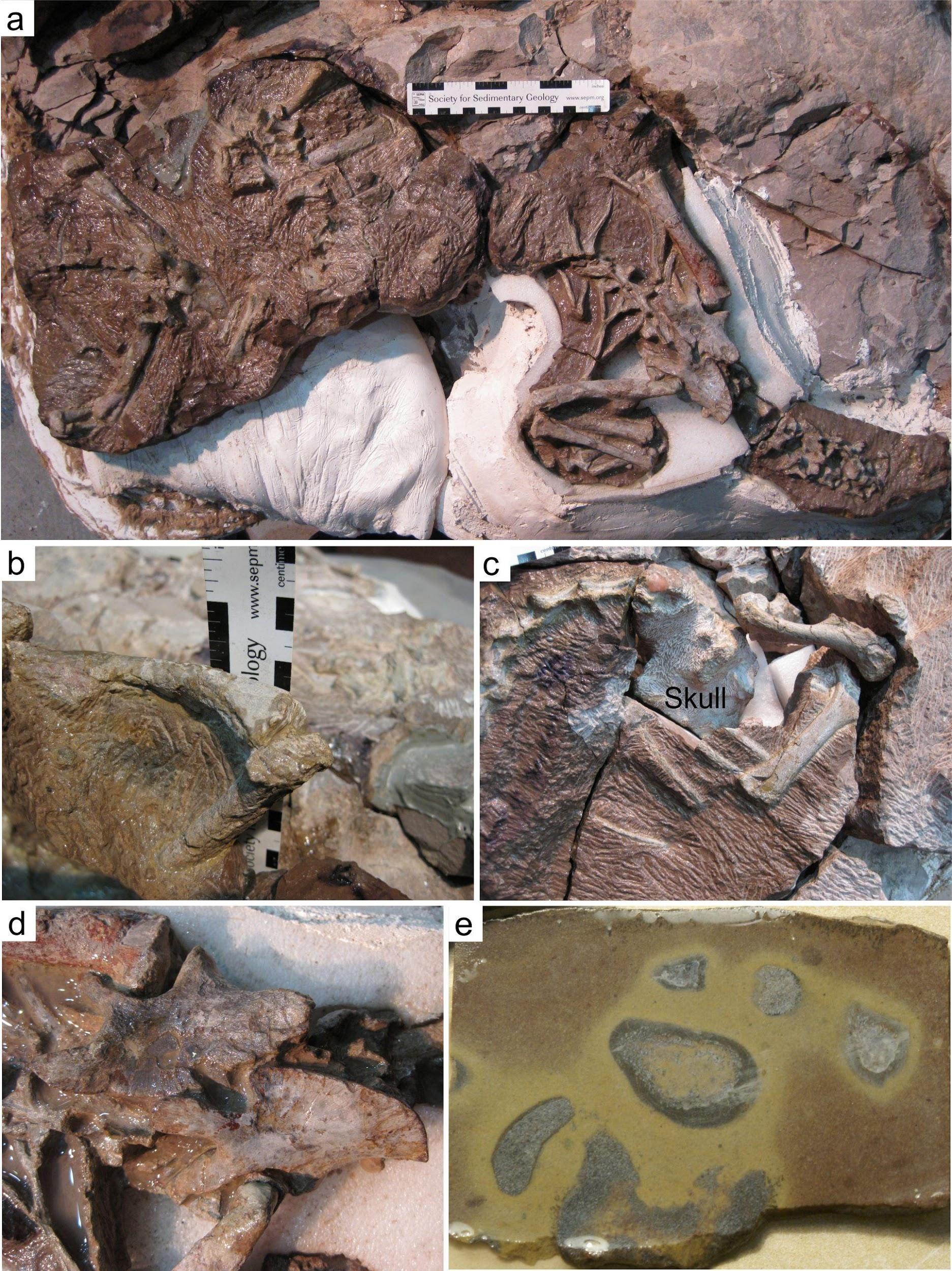


**Supplementary Figure 12.** Taphonomy of the juveniles of *M. patagonicus* MPM-PV 1813. **a**. Disarticulation of the anterior part of the body and articulation and life-like crouched position of the posterior part body. **b**. Posterior limb crosscutting the sediment. **c**. Two specimens intertwined, the skull of one individual below the anterior limb, which crosscuts the sediment, of a second individual. **d**. Left and right ilia show different weathering features depending on how close to the top surface the bone is located. **e**. Colour haloes in the sediment around the bones of a flexed hindlimb (cross section of distal femur at bottom, tibia and fibula in between, and three metatarsals on top).

**6. Preservation of embryonic remains of *Mussaurus patagonicus***

Field observations and preliminary X-ray micro-CT scanning revealed the presence of embryonic remains in some of the eggs collected at the Laguna Colorada Formation. A total of 30 eggs were analysed at the European Synchrotron Radiation Facility (Beamline ID19, ESRF, Grenoble, France).

*Taxonomic identification.* The embryonic remains detected inside the eggs bear an autapomorphic trait of *Mussaurus patagonicus* that allows identifying them taxonomically. The anterodorsal end of the symphyseal end of the dentaries in *Mussaurus* bear a noticeable process that has been described as an autapomorphy of this taxon by Pol and Powell (2007) based on its presence in the type material as well as the juveniles MPM-PV 1813. The embryo of the egg specimen MPM-PV 1879 has preserved this feature (Suppl. Fig. 5). The anterior end of the dentary is dorsoventrally expanded with the ventral margin describing a rather strong downturned profile, resembling the condition of the dentary of the neonates and the juveniles of *Mussaurus patagonicus*.

**7. Synchrotron X-ray micro-Computed Tomography**

The egg MPM-PV 1879, the neonate MACN-PV 4111 and two skulls of juvenile, MPM-PV 1813/2 and MPM-PV 1813/4, were scanned at the ID19 beamline of the ESRF using propagation phase contrast synchrotron X-ray micro-Computed Tomography (PPC-SRµCT).

**Supplementary Table 3:** List of parameters used to scan the 4 specimens presented in this study. The two juvenile skulls were scanned together to compensate for their high aspect ratio (small thickness vs. large width). The energy indicated is the detected total integrated energy. Scint., scintillator (material and thickness); voxel size based on pixel size measured considering the magnification from the propagation distance; COR shift, shift of the centre of rotation in pixels; ODD, Object-Detector distance; proj. number of projections per complete acquisition; exp (ms), total exposure per projection in milli-seconds; acc., number of images accumulated per projection; nb of scans, number of scan necessary to cover the specimens on the vertical axis.

| Specimen | Wiggler gap (mm) | Filter (mm) | Energy (keV) | Scint. | voxel size | COR shift | ODD | proj. | exp.  (ms) | acc. | nb of scans |
| --- | --- | --- | --- | --- | --- | --- | --- | --- | --- | --- | --- |
| MPM-PV 1813-4 and 1813-2 | 26.5 | Cu: 12 | 167 | LuAG 2 mm | 24.6 µm | 1000 | 12 m | 6000 | 60 | 3 | 37 |
| MACN-PV 4111 skeleton | 26.5 | Cu: 12 | 167 | LuAG 2 mm | 24.6 µm | 800 | 12 m | 4000 | 60 | 3 | 32 |
| MACN-PV 4111 skull | 58 | Al: 2.8 Cu: 6 | 107 | GGG 1 mm | 8.94 µm | 1200 | 3 m | 6000 | 75 | 5 | 7 |
| MPM-PV 1879 | 56 | Al: 2.8 Cu: 6 | 109 | GGG 1 mm | 8.94 µm | 1100 | 3 m | 6000 | 120 | 10 | 13 |

**8. Body mass estimates of *Mussaurus* *patagonicus* specimens**

Body mass estimates of the different specimens of *Mussaurus patagonicus* were calculated for the specimens that were used for the histological analysis (see below). Results of the scaling equation developed by Campione et al. (2014, 2017) for bipedal non-avian dinosaurs are given in the following section (Suppl. Table 4).

The body mass estimates produced by the scaling equation differ in some cases with those inferred using a volumetric approach (Otero et al., 2019) for *Mussaurus patagonicus*. The estimated body mass of the neonates following the volumetric approach was 0.0679 kg (average spline-based reconstruction; see Otero et al., 2019) whereas using the scaling equation we obtained the almost identical value of 0.0685 kg. For the one-year-old juveniles the estimated mass using the volumetric approach was 8.3 kg whereas the scaling estimates yielded 10.89 kg, representing a larger relative difference between the two methods for estimating body mass. Finally, the adult specimen MLP 68-II-27-1 was used to obtain a volumetric-based body mass estimate of 1418 kg whereas its femoral circumference (28.6 cm) yields an estimate of 1096.81 kg.

The volumetric approach yields mass estimate values that are between 77% to 131% of the body mass values derived from the scaling equation cQE developed for bipedal non-avian dinosaurs by Campione et al. (2014). A systematic disparity in body mass estimates has been noted for other dinosaurs by different authors (e.g., Bates et al., 2015). Here, the variability in body mass estimates in *Mussaurus* also indicates there likely is a strong influence of the ontogenetic stage in the differences of these two approaches, likely due to the existence of ontogenetic allometry in the development of this taxon. This was in fact expected, as the ontogenetic allometry of *Mussaurus* (both in volumetric reconstructions (see Otero et al., 2019) and in humerus/femur proportions (see Chapelle et al., 2019) led to the proposition of a postural change throughout the ontogeny of this taxon.


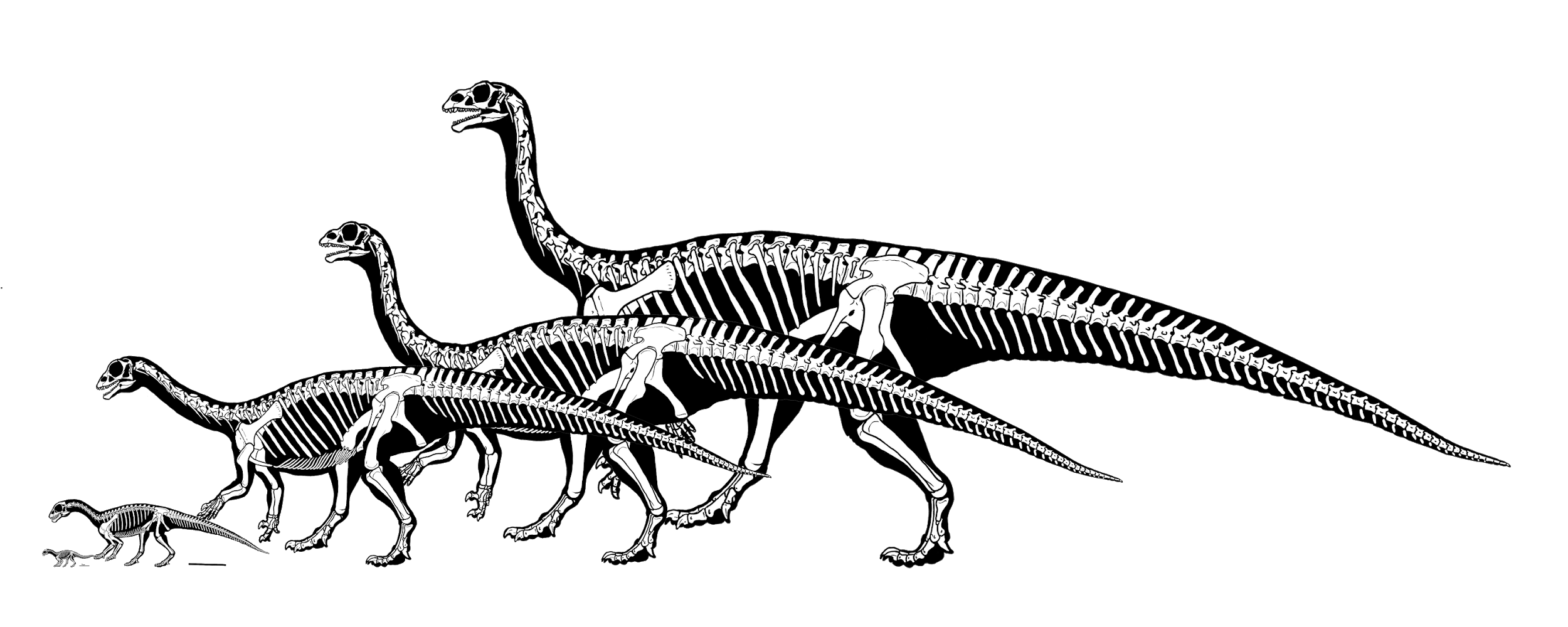


**Supplementary Figure 13.** Growth series of *Mussaurus patagonicus* from neonates to adult specimens. Drawing by J. Gonález.

**9- Histological analysis of *Mussaurus patagonicus***

Histological samples of the femora were obtained to assess the ontogenetic stage of four individuals (MPM-PV 1813/10, 1836, 1838, and MLP 61-III-20-22). In those cases in which a complete element was sampled, a complete section of around 1 cm was obtained from the bone and, to avoid loss of information about the anatomy and size, a cast of the extracted sample was generated and used to reconstruct the sampled bone. The preparation of the histological sections was carried out in the Museo Paleontológico Egidio Feruglio (Trelew, Argentina). Nomenclature and definitions of structures used in this study are derived from Francillon-Vieillot et al. (1990) and Chinsamy-Turan (2005). Regarding growth marks, we follow the terminology of Francillon-Vieillot et al. (1990), in which lines of arrested growth (LAGs) are a type of cementing line that represents temporary but complete cessation of appositional growth. The annuli are narrow layers of parallel fibered bone or lamellar bone that reflect periods of relatively slow growth rates. The results presented here expand the preliminary histological description of specimens MPM-PV 1813/10 and MLP 61-III-20-22 that appeared in previous papers (Cerda et al. 2014a, 2014b, 2017).

The smaller specimen sampled (MPM-PV 1813/10) possess wide vascular spaces arranged in a laminar to plexiform pattern, with predominance of radial anastomoses in some areas (Suppl. Fig. 14a). The cortex is entirely formed by fibro-lamellar bone tissue consisting of a woven-fibered matrix with large open channels or only weakly developed primary osteons (Suppl. Fig. 14b). Several laminae of vascular spaces are partially resorbed at the perimedullary margin. Secondary osteons and lines of arrested growth are entirely absent in the compacta.

In specimen MPM-PV 1836, the cortical bone is entirely composed of well vascularized fibro-lamellar bone tissue (Suppl. Fig. 14c). Intrinsic fibers become slightly ordered in the inner area of the posterior region of the cortex. Vascularization is important in the whole compacta and primary osteons are mainly organized in a laminar to plexiform pattern, with predominance of circumferential canals. Abundant Sharpey’s fibers are imbedded in the bone matrix at the anterior region. A single LAG is clearly observed in the perimedullary cortex (Suppl. Fig. 14d). Large spaces of resorption are located at the anterior region of the perimedullary cavity. Few secondary osteons, often forming circumferential laminae, are scattered in the inner cortex.

The cortical bone in the specimen MPM-PV 1838 is mostly primary in origin. Parallel-fibred bone predominates in the whole cortex, with its birefringence grading from moderate (in the inner to mid cortex) to strong (in the outer cortex) (Suppl. Fig. 14e). Woven-fibered bone is observed in some areas (Suppl. Fig. 14f). Except in the outermost cortex, where the vascularization strongly decreases, vascular spaces are extensively developed throughout the compacta. The arrangement of the vascular canals is mostly plexiform with a predominance of circumferential canals (Fig. 8g). LAGs are developed in the outer third of the cortex. A minimum of twelve of these growth marks (including double LAGs) were counted. LAGs are more closely spaced at the outer cortex, where they are accompanied by some annuli consisting of avascular parallel-fibred bone (Suppl. Fig. 14h). Secondary osteons are abundant at the inner cortex. They are especially numerous in the antero-lateral, antero-medial and postero-medial areas.

The compact bone of the largest specimen (MLP 61-III-20-22) consists predominantly of highly vascularized fibro-lamellar bone (Fig. 8i). The fibrous matrix of the fibro-lamellar tissue is not regularly woven and, under polarized light, several regions of the bone appear to be well-organized (showing general anisotropy under crossed nicols) (Suppl. Fig. 14j). Vascular channels tend to mostly have a plexiform arrangement (Suppl. Fig. 14k), but variation on this regard occurs in different portions of the compacta. At least eight lines of arrested growth are observed in the compacta, but these are restricted to the outer third of the cortex. These growth marks tend to be more closely spaced toward the external part of the cortex (Suppl. Fig. 14l). Closely spaced growth lines indicative of substantial truncation in growth appears to be preserved at the lateral part of the cross section, however, given that the most external part of the cortex has been eroded it is not possible to confirm that this reflects a cessation of growth. Secondary osteons are commonly developed in the mid- to perimedullary region of the cortex. In the anteromedial and anterolateral regions of the cross section, secondary osteons form two wide “columns” of dense Haversian bone. Large resorption cavities are present in the perimedullary region.


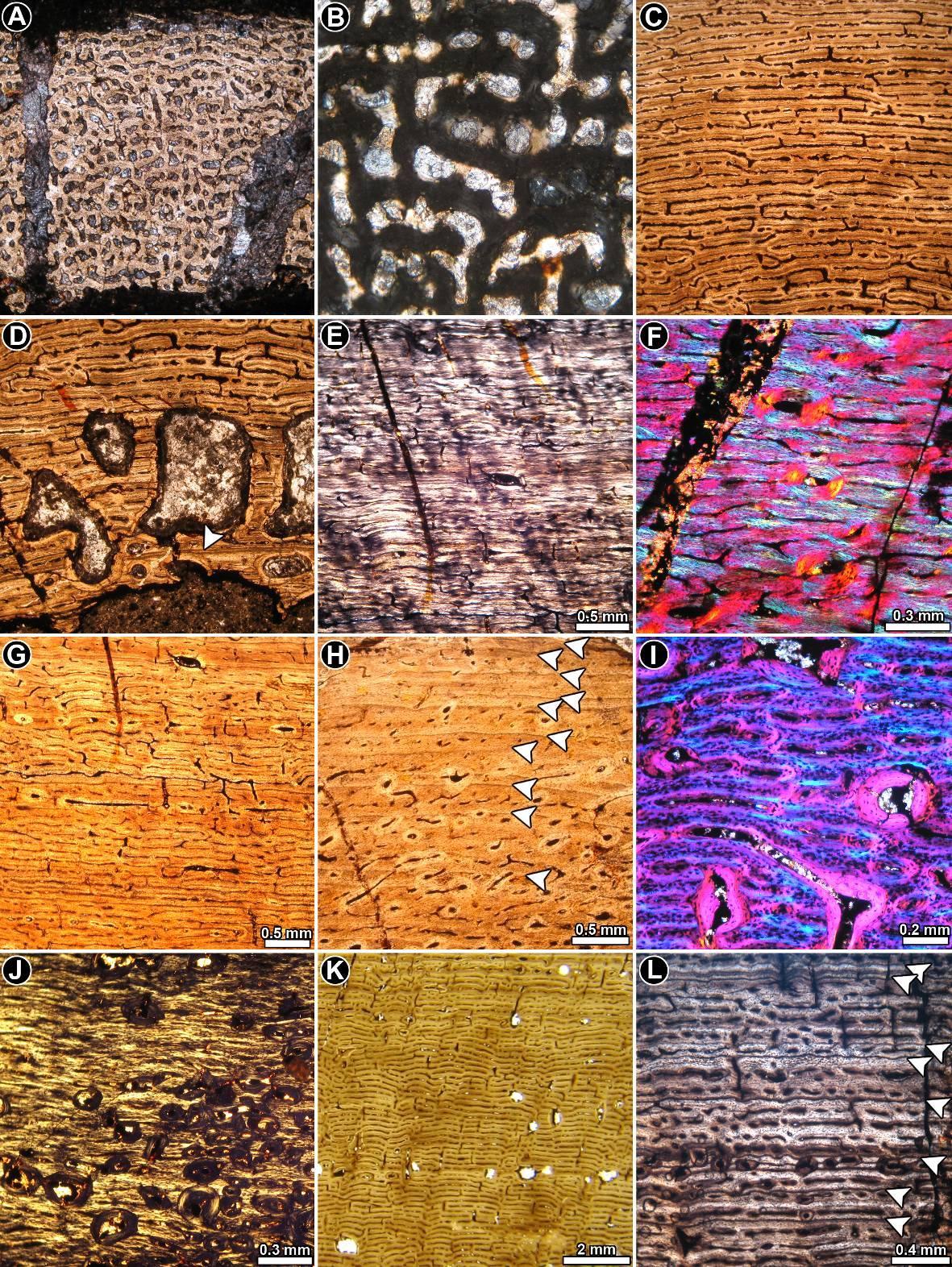


**Supplementary Figure 14**: Femoral bone histology of different ontogenetic stages of *Mussaurus patagonicus* from Laguna Colorada Locality. **A, B**: MPM-PV 1813/10, **A**- Cortical bone in cross section. The cortex is formed by a well-vascularized woven fibred bone matrix. **B**- Detail of the same section viewed under polarized light. **C, D**: MPM-PV 1836, **C**- General view of the mid cortex showing highly vascularized primary bone tissue. **D**- Line of arrested growth (arrowhead) in the perimedullary region of the cortex. Note the presence of large resorption cavities. **E**-**H**: MPM-PV 1838, **E**- Detailed view of the outer cortex showing predominance of parallel-fibred bone tissue. **F**- Detail of the fibrolamellar bone tissue in the mid cortex. **G**- Primary bone tissue showing a plexiform pattern of vascularisation, with a predominance of circumferential canals. **H**- Closely spaced lines of arrested growth (arrowheads) in the outer cortex. **I-L**: MLP 61-III-20-22, **I**- Detailed view of the fibrolamellar bone tissue. Primary bone is interrupted by some secondary osteons. **J**- Detail of the cortical bone showing predominance of parallel-fibred bone tissue. **K**- Detail of the highly vascularised primary bone. Vascular canals exhibit a plexiform pattern. **L**- Detailed view of the outer cortex showing several lines of arrested growth (arrowheads). A, C, D, G, H, K. L: Normal transmitted light. B, E: Polarized light. F, I: Polarized light with lambda compensator.

**Supplementary Table 4. Histological features of selected specimens of *M. patagonicus*. PFB: parallel fibered bone; WFB: woven fibered bone**

| Stage | Specimen number | Body Mass  (kg) | Circumference at midshaft (mm) | Matrix | Growth marks | Secondary osteons |
| --- | --- | --- | --- | --- | --- | --- |
| 1 | PVL 4068 | 0.07 | 8.4 | N/A | N/A | N/A |
| 2 | MPM-PV 1813/10 | 10.89 | 52 | WFB | Absent | Absent |
| 3 | MPM-PV 1836 | 106.23 | 120* | WFB | One | Scarce |
| 4 | MPM-PV 1838 | 556.98 | 222 | PFB>WFB | 12 | Abundant at the perimedullary region |
| 5 | MLP 61-III-20-22 | 1504.76 | 322 | WFB>>PFB | 8 | Abundant at the perimedullary region |

*incomplete

**10. Phylogenetic analysis of *Mussaurus patagonicus***

***10.a. Analytical procedures***

The phylogenetic analysis was based on a data matrix of 76 taxa scored for a total of 419 characters. The complete character list with sources for each of the characters is detailed below.

The analysis in TNT (Goloboff et al., 2008) recovered 37386 MPTs of 1672 steps (CI=0.295, RI=0.743). The strict consensus (see below 10.b) has large polytomies in basal nodes of Sauropodomorpha. Applying the IterPCR (Pol and Escapa, 2009) implemented in TNT (Goloboff and Szumik, 2015) identified 7 unstable taxa that cause these polytomies: *Nambalia*, *Jaklapalisaurus*, *Seitaad*, *Leonerasaurus*, *Sefapanosaurus*, *Blikanasaurus*, and *Guaibasaurus*. Ignoring their position in the MPTs results in a much more resolved reduced strict consensus (see below 10.c).

***10.b. Strict consensus***

**
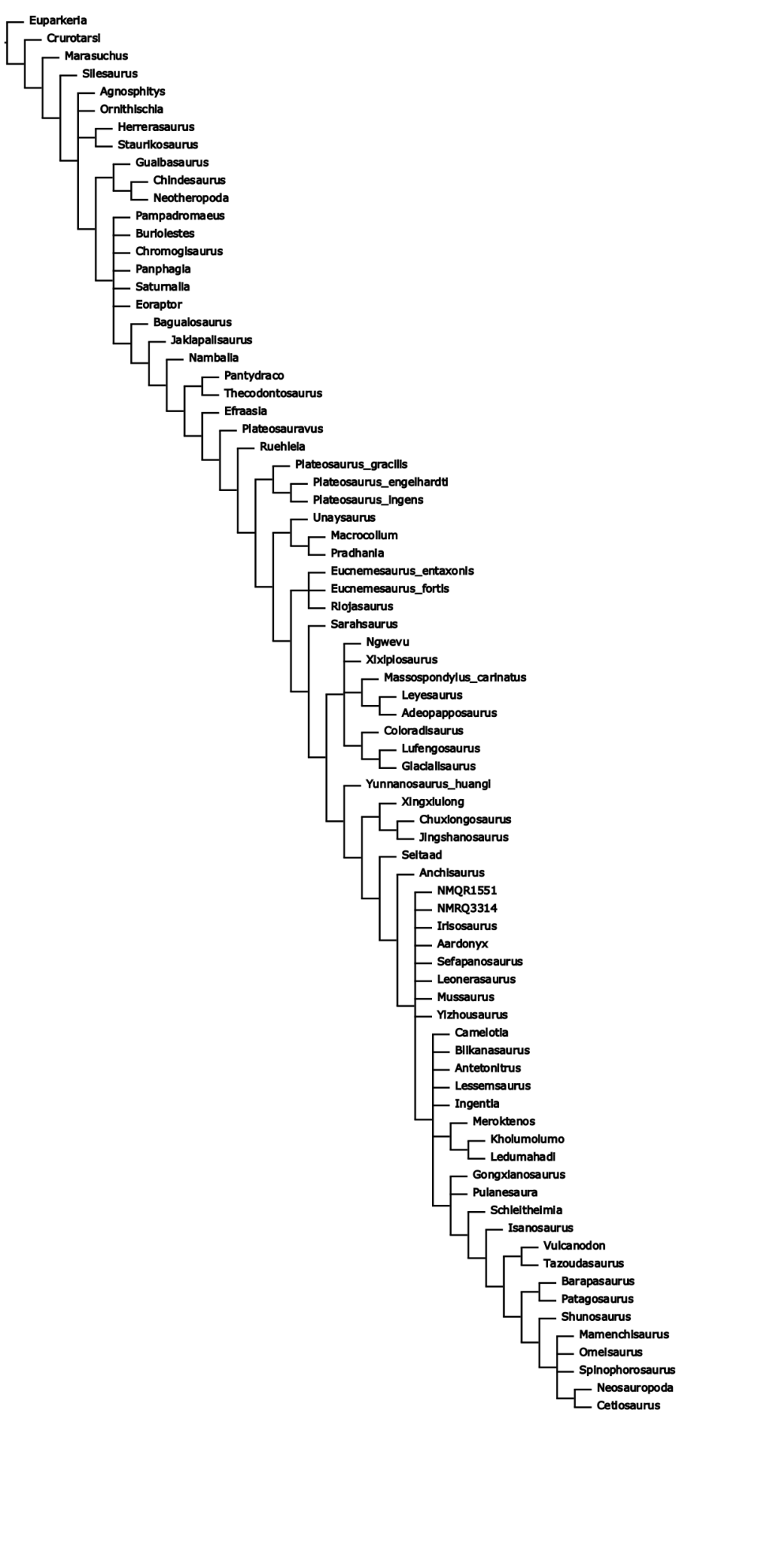
**

***10.c. Reduced consensus***


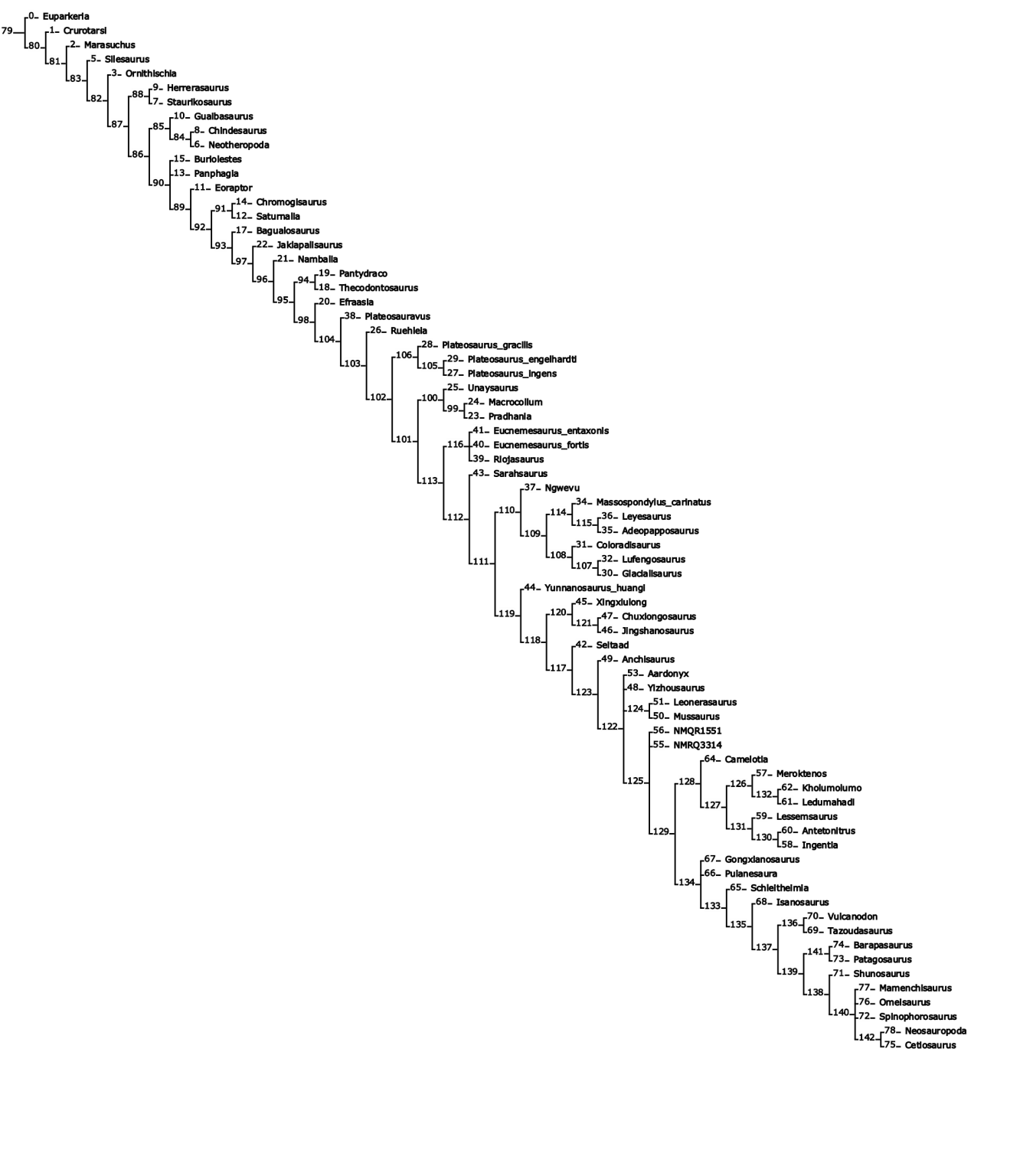


***10.d. Calibration against geological time***

The most parsimonious trees were calibrated against geological time using the R packages Paleotree (Bapst, 2012) and Strap (Bell and Lloyd, 2015). Zero length branches were treated with a minimum branch length of 0.3 million years. A calibrated reduced consensus was used for displaying the relationships of early sauropodomorphs in Figure 3. The following text was input for the calibration of trees in R, detailing the first appearance datum (FAD) and last appearance datum (LAD) for each of the terminal taxon.

FAD LAD

Euparkeria 247.2 242

Crurotarsi 247.2 145.5

Marasuchus 236.1 233.7

Ornithischia 231.4 145.5

Agnosphitys 208.5 201.3

Silesaurus 236 227

Neotheropoda 227 145.5

Staurikosaurus 235 221.5

Chindesaurus 227 208.5

Herrerasaurus 231.4 225.9

Eoraptor 231.4 225.9

Saturnalia 233.2 225.4

Panphagia 231.4 225.9

Chromogisaurus 231.4 225.9

Buriolestes 233.2 225.4

Guaibasaurus 225.4 220

Pampadromaeus 233.2 225.4

Bagualosaurus 233.2 225.4

Nambalia 227 208.5

Thecodontosaurus 208.5 201.3

Pantydraco 208.5 201.3

Efraasia 215.56 212

Ruehleia 227 208.5

Jaklapalisaurus 227 208.5

Macrocollum 225.4 220

Unaysaurus 225.4 220

Plateosaurus_ingens 212 205.6

Plateosaurus_gracilis 227 208.5

Plateosaurus_engelhardti 227 201.3

Pradhania 199.3 190.8

Glacialisaurus 199 182

Coloradisaurus 220 213

Yunnanosaurus_huangi 201.3 190.8

Lufengosaurus 201.3 190.8

Xixipiosaurus 201.3 190.8

Massospondylus_carinatus 202.3 187.5

Adeopapposaurus 201.3 190.8

Leyesaurus 201.3 190.8

Plateosauravus 219.6 202.3

Riojasaurus 220 213

Eucnemesaurus_fortis 219.6 202.3

Eucnemesaurus_entaxonis 219.6 202.3

Seitaad 190.8 182.7

Anchisaurus 201.3 190.8

Chuxiongosaurus 201.3 190.8

Jingshanosaurus 201.3 190.8

Xingxiulong 201.3 190.8

Sarahsaurus 199.3 183.7

Yizhousaurus 201.3 190.8

Kholumolumo 219.6 202.3

Mussaurus 192.7 192.6

Leonerasaurus 189 188.8

Sefapanosaurus 219.6 202.3

Aardonyx 202.3 187.5

Meroktenos 219.6 202.3

NMRQ3314 202.3 187.5

NMQR1551 219.6 202.3

Ingentia 213 201.3

Lessemsaurus 220 213

Antetonitrus 202.3 187.5

Ledumahadi 202.3 187.5

Blikanasaurus 219.6 202.3

Camelotia 208.5 201.3

Pulanesaura 202.3 187.5

Gongxianosaurus 182.7 174.1

Isanosaurus 208.5 174.1

Tazoudasaurus 190.8 174.1

Vulcanodon 199.3 182.7

Shunosaurus 161 157

Spinophorosaurus 174.1 163.5

Patagosaurus 178.7 178.1

Barapasaurus 190.8 170.3

Cetiosaurus 170.3 163.5

Omeisaurus 161 157

Mamenchisaurus 163.5 152.1

Neosauropoda 166.1 145.5

***10.e. List of synapomorphies***

The following list of unambiguous synapomorphies were obtained from the pool of 37,386 most parsimonious trees after pruning the unstable taxa listed above. Node numbers refer to those present in the Reduced consensus tree (see above 10.c).

Node 81 :

All trees:

Char. 120: 0 --> 1

Char. 203: 0 --> 1

Char. 249: 1 --> 0

Char. 278: 0 --> 1

Char. 286: 0 --> 1

Char. 287: 0 --> 1

Char. 340: 1 --> 0

Char. 351: 0 --> 1

Node 82 :

All trees:

Char. 133: 0 --> 1

Char. 144: 0 --> 1

Char. 177: 0 --> 1

Char. 206: 0 --> 1

Char. 250: 0 --> 2

Char. 318: 0 --> 1

Char. 381: 0 --> 1

Char. 382: 0 --> 1

Node 83 :

All trees:

Char. 47: 0 --> 1

Char. 83: 0 --> 1

Char. 199: 0 --> 1

Char. 202: 0 --> 1

Char. 204: 2 --> 1

Char. 258: 1 --> 0

Char. 270: 0 --> 1

Char. 283: 0 --> 1

Char. 293: 0 --> 1

Char. 303: 0 --> 1

Char. 324: 0 --> 1

Char. 371: 0 --> 1

Char. 379: 0 --> 1

Node 84 :

All trees:

Char. 259: 0 --> 1

Char. 293: 1 --> 0

Char. 294: 1 --> 0

Char. 325: 0 --> 1

Node 85 :

All trees:

Char. 159: 0 --> 1

Char. 257: 0 --> 1

Char. 261: 0 --> 1

Char. 305: 0 --> 1

Char. 307: 0 --> 1

Char. 317: 0 --> 1

Some trees:

Char. 155: 0 --> 1

Node 86 :

All trees:

Char. 7: 0 --> 12

Char. 14: 0 --> 1

Char. 17: 0 --> 1

Char. 18: 0 --> 1

Char. 23: 0 --> 1

Char. 37: 0 --> 1

Char. 101: 0 --> 1

Char. 216: 0 --> 1

Char. 241: 0 --> 1

Char. 254: 0 --> 2

Char. 276: 0 --> 1

Char. 339: 0 --> 1

Char. 359: 0 --> 1

Char. 372: 0 --> 1

Char. 376: 0 --> 1

Char. 383: 0 --> 1

Node 87 :

All trees:

Char. 12: 0 --> 1

Char. 61: 0 --> 1

Char. 67: 0 --> 1

Char. 76: 0 --> 1

Char. 126: 0 --> 1

Char. 127: 0 --> 1

Char. 156: 0 --> 1

Char. 220: 0 --> 1

Char. 221: 0 --> 2

Char. 227: 0 --> 1

Char. 228: 0 --> 1

Char. 243: 0 --> 1

Char. 320: 0 --> 1

Char. 328: 0 --> 1

Char. 337: 0 --> 1

Char. 377: 0 --> 1

Node 88 :

All trees:

Char. 102: 0 --> 1

Char. 150: 0 --> 1

Char. 166: 01 --> 2

Char. 173: 0 --> 1

Char. 179: 0 --> 1

Char. 182: 0 --> 1

Char. 188: 0 --> 1

Char. 192: 0 --> 1

Char. 198: 0 --> 1

Char. 200: 0 --> 1

Char. 264: 0 --> 1

Node 89 :

All trees:

Char. 273: 0 --> 1

Char. 385: 0 --> 1

Some trees:

Char. 26: 0 --> 1

Char. 32: 0 --> 1

Char. 246: 0 --> 1

Node 90 :

All trees:

Char. 3: 0 --> 1

Char. 22: 0 --> 1

Char. 60: 0 --> 1

Char. 73: 0 --> 1

Char. 74: 0 --> 1

Char. 99: 0 --> 1

Char. 107: 0 --> 1

Char. 141: 0 --> 1

Char. 142: 0 --> 1

Char. 250: 2 --> 1

Char. 303: 1 --> 0

Some trees:

Char. 301: 0 --> 1

Node 91 :

All trees:

Char. 370: 0 --> 1

Node 92 :

All trees:

Char. 0: 0 --> 1

Char. 61: 1 --> 0

Char. 183: 0 --> 1

Char. 210: 0 --> 1

Char. 298: 0 --> 1

Some trees:

Char. 251: 0 --> 1

Char. 301: 1 --> 0

Node 93 :

All trees:

Char. 332: 0 --> 1

Char. 352: 0 --> 1

Char. 371: 2 --> 1

Some trees:

Char. 113: 0 --> 1

Node 94 :

All trees:

Char. 93: 1 --> 0

Char. 135: 0 --> 1

Char. 255: 1 --> 0

Char. 305: 1 --> 0

Node 95 :

All trees:

Char. 249: 0 --> 1

Char. 333: 0 --> 1

Node 96 :

All trees:

Char. 383: 1 --> 0

Node 97 :

All trees:

Char. 305: 0 --> 1

Node 98 :

All trees:

Char. 20: 0 --> 1

Char. 22: 1 --> 2

Char. 84: 0 --> 1

Char. 103: 0 --> 1

Char. 104: 0 --> 1

Char. 176: 0 --> 1

Char. 190: 0 --> 1

Char. 211: 0 --> 1

Char. 226: 0 --> 1

Char. 228: 1 --> 0

Char. 243: 1 --> 0

Char. 250: 2 --> 3

Char. 342: 0 --> 1

Char. 352: 1 --> 2

Node 99 :

All trees:

Char. 226: 1 --> 0

Char. 233: 1 --> 2

Node 100 :

All trees:

Char. 210: 1 --> 0

Node 101 :

All trees:

Char. 7: 1 --> 2

Char. 278: 1 --> 0

Char. 283: 1 --> 0

Char. 352: 3 --> 2

Char. 405: 0 --> 1

Node 102 :

All trees:

Char. 172: 0 --> 1

Char. 189: 1 --> 0

Char. 219: 0 --> 1

Char. 277: 0 --> 1

Char. 387: 0 --> 1

Node 103 :

All trees:

Char. 204: 2 --> 1

Char. 257: 1 --> 2

Char. 391: 2 --> 1

Node 104 :

All trees:

Char. 148: 0 --> 12

Char. 252: 0 --> 1

Char. 260: 0 --> 1

Char. 352: 2 --> 3

Node 105 :

All trees:

Char. 352: 3 --> 4

Node 106 :

All trees:

Char. 54: 0 --> 1

Char. 80: 0 --> 1

Char. 106: 0 --> 1

Char. 176: 1 --> 0

Char. 177: 0 --> 1

Char. 268: 0 --> 1

Node 107 :

All trees:

Char. 330: 0 --> 1

Node 108 :

All trees:

Char. 80: 0 --> 1

Char. 124: 1 --> 0

Char. 266: 1 --> 2

Char. 353: 0 --> 1

Char. 355: 0 --> 1

Char. 356: 0 --> 1

Char. 357: 0 --> 1

Node 109 :

All trees:

Char. 98: 0 --> 1

Char. 148: 1 --> 2

Char. 317: 0 --> 1

Char. 413: 1 --> 0

Char. 414: 0 --> 1

Node 110 :

All trees:

Char. 72: 0 --> 1

Char. 130: 1 --> 2

Char. 415: 1 --> 0

Node 111 :

All trees:

Char. 13: 0 --> 1

Char. 30: 0 --> 1

Char. 31: 1 --> 0

Char. 39: 0 --> 1

Char. 54: 0 --> 1

Char. 119: 0 --> 1

Char. 233: 1 --> 2

Char. 267: 1 --> 0

Char. 283: 0 --> 1

Char. 285: 1 --> 0

Node 112 :

All trees:

Char. 51: 0 --> 1

Char. 134: 0 --> 1

Char. 199: 1 --> 0

Char. 213: 0 --> 1

Char. 224: 0 --> 1

Char. 226: 1 --> 2

Char. 263: 0 --> 1

Char. 325: 0 --> 1

Char. 339: 1 --> 0

Node 113 :

All trees:

Char. 22: 2 --> 1

Char. 28: 0 --> 1

Char. 56: 0 --> 1

Char. 182: 0 --> 1

Char. 207: 0 --> 1

Char. 230: 0 --> 1

Char. 241: 1 --> 2

Char. 281: 0 --> 1

Char. 295: 0 --> 1

Char. 343: 0 --> 1

Node 114 :

All trees:

Char. 25: 1 --> 0

Char. 104: 1 --> 0

Char. 207: 1 --> 0

Char. 234: 0 --> 1

Char. 295: 1 --> 0

Char. 394: 1 --> 0

Char. 396: 0 --> 1

Char. 405: 1 --> 0

Node 115 :

All trees:

Char. 52: 0 --> 1

Char. 93: 1 --> 0

Char. 109: 0 --> 1

Char. 136: 1 --> 0

Char. 327: 1 --> 0

Node 116 :

All trees:

Char. 189: 0 --> 1

Char. 282: 0 --> 1

Char. 288: 0 --> 1

Char. 294: 1 --> 0

Some trees:

Char. 277: 1 --> 0

Node 117 :

All trees:

Char. 206: 2 --> 1

Char. 224: 1 --> 0

Char. 263: 1 --> 0

Node 118 :

All trees:

Char. 95: 0 --> 1

Char. 116: 0 --> 1

Char. 277: 1 --> 0

Char. 324: 1 --> 0

Node 119 :

All trees:

Char. 207: 1 --> 0

Char. 221: 1 --> 0

Char. 252: 1 --> 0

Char. 405: 1 --> 0

Char. 416: 0 --> 1

Node 120 :

All trees:

Char. 266: 1 --> 2

Char. 281: 1 --> 0

Node 121 :

All trees:

Char. 38: 0 --> 1

Char. 45: 0 --> 1

Node 122 :

All trees:

Char. 189: 0 --> 1

Char. 232: 0 --> 1

Char. 279: 0 --> 1

Char. 352: 2 --> 3

Char. 366: 0 --> 1

Some trees:

Char. 96: 1 --> 0

Char. 355: 0 --> 1

Node 123 :

All trees:

Char. 310: 0 --> 1

Node 124 :

All trees:

Char. 153: 1 --> 0

Some trees:

Char. 152: 1 --> 0

Char. 177: 0 --> 1

Char. 178: 0 --> 1

Char. 248: 0 --> 1

Node 125 :

All trees:

Char. 172: 1 --> 0

Char. 289: 0 --> 1

Char. 290: 0 --> 1

Some trees:

Char. 5: 0 --> 1

Char. 7: 2 --> 3

Char. 25: 1 --> 0

Char. 41: 0 --> 1

Char. 67: 1 --> 2

Char. 89: 0 --> 1

Char. 93: 1 --> 0

Char. 156: 1 --> 2

Char. 166: 01 --> 1

Char. 177: 0 --> 1

Char. 178: 0 --> 1

Char. 204: 1 --> 2

Char. 211: 1 --> 0

Char. 280: 0 --> 1

Char. 343: 1 --> 2

Char. 392: 0 --> 1

Node 126 :

All trees:

Char. 282: 0 --> 1

Char. 286: 1 --> 0

Char. 289: 1 --> 0

Char. 290: 1 --> 0

Node 127 :

All trees:

Char. 163: 0 --> 1

Char. 165: 0 --> 1

Node 128 :

All trees:

Char. 131: 0 --> 1

Char. 292: 0 --> 1

Node 129 :

All trees:

Char. 116: 1 --> 2

Char. 132: 0 --> 1

Char. 309: 1 --> 0

Char. 353: 0 --> 1

Some trees:

Char. 1: 0 --> 1

Char. 319: 0 --> 1

Node 130 :

All trees:

Char. 144: 1 --> 0

Node 131 :

All trees:

Char. 199: 1 --> 0

Char. 226: 2 --> 3

Node 132 :

All trees:

Char. 280: 1 --> 0

Node 133 :

All trees:

Char. 146: 0 --> 1

Char. 186: 0 --> 1

Char. 253: 0 --> 1

Node 134 :

All trees:

Char. 157: 1 --> 0

Char. 159: 0 --> 1

Char. 180: 0 --> 1

Char. 389: 0 --> 1

Char. 403: 0 --> 1

Some trees:

Char. 185: 0 --> 1

Char. 200: 0 --> 1

Char. 214: 0 --> 1

Char. 282: 0 --> 1

Char. 284: 1 --> 2

Char. 340: 0 --> 1

Char. 348: 1 --> 2

Node 135 :

All trees:

Char. 143: 01 --> 2

Node 136 :

All trees:

Char. 282: 1 --> 0

Char. 284: 2 --> 1

Char. 292: 0 --> 1

Char. 314: 0 --> 1

Char. 347: 0 --> 1

Char. 361: 0 --> 1

Node 137 :

All trees:

Char. 168: 0 --> 1

Char. 169: 1 --> 2

Char. 291: 1 --> 0

Char. 352: 4 --> 5

Node 138 :

All trees:

Char. 293: 1 --> 0

Some trees:

Char. 129: 0 --> 1

Char. 154: 0 --> 1

Char. 196: 0 --> 1

Char. 295: 1 --> 0

Char. 331: 0 --> 1

Node 139 :

All trees:

Char. 94: 0 --> 1

Char. 100: 0 --> 1

Char. 158: 0 --> 1

Char. 265: 0 --> 1

Char. 299: 0 --> 1

Char. 345: 0 --> 1

Some trees:

Char. 203: 1 --> 0

Char. 297: 0 --> 1

Node 140 :

All trees:

Char. 58: 0 --> 1

Char. 71: 0 --> 1

Char. 204: 2 --> 3

Char. 292: 0 --> 1

Char. 403: 2 --> 0

Some trees:

Char. 11: 0 --> 1

Char. 48: 0 --> 1

Char. 93: 0 --> 1

Char. 125: 0 --> 1

Char. 138: 0 --> 1

Char. 226: 1 --> 0

Char. 236: 0 --> 1

Char. 304: 0 --> 1

Char. 336: 0 --> 1

Node 141 :

All trees:

Char. 161: 0 --> 1

Char. 162: 0 --> 1

Char. 165: 0 --> 1

Node 142 :

All trees:

Char. 128: 0 --> 1

Char. 160: 0 --> 1

Char. 253: 1 --> 2

Some trees:

Char. 129: 1 --> 0

Char. 131: 0 --> 1

Char. 138: 1 --> 0

Char. 146: 1 --> 2

Char. 196: 1 --> 0

Char. 269: 0 --> 1

Char. 295: 0 --> 1

***10.f. Character List***

The complete list of characters used in the phylogenetic analysis is provided here. The following multistate characters were treated as ordered: 8, 13, 19, 23, 40, 57, 69, 92, 102, 108, 117, 121, 134, 144, 147, 149, 150, 157, 167, 170, 171, 177, 183, 205, 207, 214, 222, 227, 242, 251, 254, 277, 299, 336, 342, 349, 353, 370, 393, 404, and 409.

This character list includes the characters listed by Yates (2007) and the characters added by Smith and Pol (2007), Ezcurra (2010), Novas et al. (2011), Apaldetti *el al.* (2012), Otero and Pol (2013), Otero et al. (2015), McPhee et al. (2015, 2017), McPhee and Choiniere (2017), Bronzati and Rauhut (2017), Bronzati et al. (2018), Cerda et al. (2017), Chapelle and Choiniere (2018), and Pol et al. (2021).

1. Skull to femur ratio: greater than (0), or less than (1), 0.6 (modified from Gauthier 1986).

2. Lateral plates appressed to the labial side of the premaxillary, maxillary and dentary teeth: absent (0) or present (1) (Upchurch, 1995).

3. Relative height of the rostrum at the posterior margin of the naris: more than (0), or less than (1), 0.6 of the height of the skull at the middle of the orbit (Langer, 2004).

4. Foramen on the lateral surface of the premaxillary body: absent (0) or present (1).

5. Distal end of the dorsal premaxillary process: tapered (0) or transversely expanded (1) (Sereno, 1999).

6. Profile of premaxilla: convex (0) or with an inflection at the base of the dorsal process (1) (Upchurch, 1995).

7. Size and position of the posterolateral process of premaxilla: large and lateral to the anterior process of the maxilla (0) or small and medial to the anterior process of the maxilla (1).

8. Relationship between posterolateral process of the premaxilla and the anteroventral process of the nasal: broad sutured contact (0), point contact (1), or briefly separated by maxilla (2), or well separated from each other by the entire posterior margin of the external nares (3) (modified from Gauthier, 1986). Ordered.

9. Posteromedial process of the premaxilla: absent (0) or present (1) (Rauhut, 2003).

10. Shape of the anteromedial process of the maxilla: narrow, elongated and projecting anterior to lateral premaxilla-maxilla suture (0) or short, broad and level with lateral premaxilla-maxilla suture (1).

11. Development of external narial fossa: absent to weak (0) or well developed with sharp posterior and anteroventral rims (1).

12. Development of narial fossa on the anterior ramus of the maxilla: weak and orientated laterally to dorsolaterally (0) or well developed and forming a horizontal shelf (1) (modified from Upchurch, 1995).

13. Size and position of subnarial foramen: absent (0), small (no larger than adjacent maxillary neurovascular foramina) and positioned outside of narial fossa (1), or large and on the rim of, or inside, the narial fossa (2) (modified from Sereno *el al.,* 1993). Ordered.

14. Shape of subnarial foramen: rounded (0) or slot-shaped (1).

15. Maxillary contribution to the margin of the narial fossa: absent (0) or present (1).

16. Diameter of external naris: less than (0), or greater than (1), 0.5 of the orbital diameter (Wilson & Sereno, 1998).

17. Shape of the external naris (in adults): rounded (0) or subtriangular with an acute posteroventral corner (1) (Galton & Upchurch, 2004).

18. Level of the anterior margin of the external naris: anterior to (0) or posterior to (1) the midlength of the premaxillary body (Rauhut, 2003).

19. Level of the posterior margin of external naris: anterior to, or level with the premaxilla-maxilla suture (0), posterior to the first maxillary alveolus (1), or posterior to the midlength of the maxillary tooth row and the anterior margin of the antorbital fenestra (2) (modified from Wilson & Sereno, 1998). Ordered.

20. Dorsal profile of the snout: straight to gently convex (0) or with a depression behind the naris (1).

21. Elongate median nasal depression: absent (0) or present (1) (Sereno, 1999).

22. Width of anteroventral process of nasal at its base: less than (0) or greater than (1) width of anterodorsal process at its base (modified from Sereno, 1999).

23. Nasal relationship with dorsal margin of antorbital fossa: not contributing to the margin of the antorbital fossa (0), lateral margin overhangs the antorbital fossa and forms its dorsal margin (1), overhang extensive, obscuring the dorsal lachrymal-maxilla contact in lateral view (2) (modified from Sereno, 1999).

24. Pointed caudolateral process of the nasal overlapping the lachrymal: absent (0) or present (1) (Sereno, 1999).

25. Anterior profile of the maxilla: slopes continuously towards the rostral tip (0) or with a strong inflection at the base of the ascending ramus, creating a rostral ramus with parallel dorsal and ventral margins (1) (Sereno *el al.,* 1996).

26. Length of rostral ramus of the maxilla: less than (0), or greater than (1), its dorsoventral depth (Sereno *el al*., 1996).

27. Shape of the main body of the maxilla: tapering posteriorly (0) or dorsal and ventral margins parallel for most of their length (1).

28. Shape of the ascending ramus of the maxilla in lateral view: tapering dorsally (0) or with an anteroposterior expansion at the dorsal end (1).

29. Rostrocaudal length of the antorbital fossa: greater (0), or less (1), than that of the orbit (Yates, 2003a).

30. Posteroventral extent of medial wall of antorbital fossa: reaching (0), or terminating anterior to (1), the anterior tip of the jugal (modified from Galton & Upchurch, 2004).

31. Development of the antorbital fossa on the ascending ramus of the maxilla: deeply impressed and delimited by a sharp, scarp-like rim (0) or weakly impressed and delimited by a rounded rim or a change in slope (1).

32. Shape of the antorbital fossa: crescentic with a strongly concave posterior margin that is roughly parallel to the rostral margin of the antorbital fossa (0), subtriangular with a straight to gently concave posterior margin (1), or antorbital fossa absent (2) (modified from Galton, 1985).

33. Size of the neurovascular foramen at the caudal end of the lateral maxillary row: not larger than the others (0) or distinctly larger than the others in the row (1) (Yates, 2003a).

34. Direction that the neurovascular foramen at the caudal end of the lateral maxillary row opens: caudally (0) or rostrally, ventrally or laterally (1) (modified from Sereno, 1999).

35. Arrangement of lateral maxillary neurovascular foramina: linear (0) or irregular (1) (modified from Sereno, 1999).

36. Longitudinal ridge on the posterior lateral surface of the maxilla: absent (0) or present (1) (Barrett , Upchurch & Wang, 2005).

37. Dorsal exposure of the lachrymal: present (0) or absent (1) (Gauthier, 1986).

38. Shape of the lachrymal: dorsoventrally short and blocks shaped (0) or dorsoventrally elongate and shaped like an inverted L (1) (Rauhut, 2003).

39. Orientation of the lachrymal orbital margin: strongly sloping anterodorsally (0) or erect and close to vertical (1).

40. Length of the anterior ramus of the lachrymal: greater than (0), or less than (1), half the length of the ventral ramus, or absent altogether (2) (modified from Galton, 1990). Ordered.

41. Web of bone spanning junction between anterior and ventral rami of lachrymal: absent and antorbital fossa laterally exposed (0) or present, obscuring posterodorsal corner of antorbital fossa (1).

42. Extension of the antorbital fossa onto the ventral end of the lachrymal: present (0) or absent (1) (modified from Wilson & Sereno, 1998).

43. Length of the caudal process of the prefrontal: short (0), or elongated (1), so that total prefrontal length is equal to the rostrocaudal diameter of the orbit (Galton, 1985).

44. Ventral process of prefrontal extending down the posteromedial side of the lachrymal: present (0) or absent (1) (Wilson & Sereno, 1998).

45. Maximum transverse width of the prefrontal: less than (0), or more than (1), 0.25 of the skull width at that level (modified from Galton, 1990).

46. Shape of the orbit: subcircular (0) or ventrally constricted making the orbit subtriangular (1) (Wilson & Sereno, 1998).

47. Slender anterior process of the frontal intruding between the prefrontal and the nasal: absent (0) or present (1) (modified from Sereno, 1999).

48. Jugal-lachrymal relationship: lachrymal overlapping lateral surface of jugal or abutting it dorsally (0), or jugal overlapping lachrymal laterally (1) (Sereno *el al.,* 1993).

49. Shape of the suborbital region of the jugal: an anteroposteriorly elongate bar (0) or an anteroposteriorly shortened plate (1).

50. Jugal contribution to the antorbital fenestra: present (0) or absent (1) (Holtz, 1994).

51. Dorsal process of the anterior jugal: present (0) or absent (1) (modified from Rauhut 2003a).

52. Ratio of the minimum depth of the jugal below the orbit to the distance between the rostral end of the jugal and the rostroventral corner of the infratemporal fenestra: less than (0), or greater than (1), 0.2 (modified from Galton, 1985).

53. Transverse width of the ventral ramus of the postorbital: less than (0), or greater than (1), its rostrocaudal width at midshaft (Wilson & Sereno, 1998).

54. Shape of the dorsal margin of postorbital in lateral view: straight to gently curved (0) or with a distinct embayment between the anterior and posterior dorsal processes (1).

55. Height of the postorbital rim of the orbit: flush with the posterior lateral process of the postorbital (0) or raised so that it projects laterally to the posterior dorsal process (1).

56. Postfrontal bone: present (0) or absent (1) (Sereno *el al.,* 1993).

57. Position of the rostral margin of the infratemporal fenestra: behind the orbit (0), extends under the rear half of the orbit (1), or extends as far forward as the midlength of the orbit (2) (modified from Upchurch, 1995). Ordered.

58. Frontal contribution to the supratemporal fenestra: present (0) or absent (1) (modified from Gauthier, 1986).

59. Orientation of the long axis of the supratemporal fenestra: longitudinal (0) or transverse (1) (Wilson & Sereno, 1998).

60. Medial margin of supratemporal fossa: simple smooth curve (0) or with a projection at the frontal ⁄ postorbital-parietal suture producing a scalloped margin (1) (Leal *el al.,* 2004).

61. Length of the quadratojugal ramus of the squamosal relative to the width at its base: less than (0), or greater than (1), four times its width (Sereno, 1999).

62. Proportion of infratemporal fenestra bordered by squamosal: more than (0), or less than (1), 0.5 of the depth of the infratemporal fenestra.

63. Squamosal-quadratojugal contact: present (0) or absent (1) (Gauthier, 1986).

64. Angle of divergence between jugal and squamosal rami of quadratojugal: close to 90° (0) or close to parallel (1).

65. Length of jugal ramus of quadratojugal: no longer than (0), or longer than (1), the squamosal ramus (Wilson & Sereno, 1998).

66. Shape of the rostral end of the jugal ramus of the quadratojugal: tapered (0) or dorsoventrally expanded (1) (Wilson & Sereno, 1998).

67. Relationship of quadratojugal to jugal: jugal overlaps the lateral surface of the quadratojugal (0), quadratojugal overlaps the lateral surface of the jugal (1), or quadratojugal sutures along the ventrolateral margin of the jugal (2). Unordered.

68. Position of the quadrate foramen: on the quadrate-quadratojugal suture (0), deeply incised into, and partly encircled by, the quadrate (1), or on the quadrate-squamosal suture, just below the quadrate head (2) (modified from Rauhut, 2003). Unordered.

69. Shape of posterolateral margin of quadrate: sloping anterolaterally from posteromedial ridge (0), everted posteriorly creating a posteriorly facing fossa (1), posterior fossa deeply excavated, invading quadrate body (2) (Wilson & Sereno, 1998). Ordered.

70. Exposure of the lateral surface of the quadrate head: absent, covered by lateral sheet of the squamosal (0) or present (1) (Sereno *el al.,* 1993).

71. Proportion of the length of the quadrate that is occupied by the pterygoid wing: at least 70% (0) or less than 70% (1) (Yates, 2003a).

72. Depth of the occipital wing of the parietal: less than (0), or more than (1), 1.5 times the depth of the foramen magnum (Wilson & Sereno, 1998).

73. Position of foramina for mid-cerebral vein on occiput: between supraoccipital and parietal (0) or on the supraoccipital (1) (modified from Yates, 2003a).

74. Postparietal fenestra between supraoccipital and parietals: absent (0) or present (1).

75. Shape of the supraoccipital: diamond-shaped, at least as high as wide (0), or semilunate and wider than high (1) (Yates, 2003b).

76. Orientation of the supraoccipital plate: erect to gently sloping (0) or strongly sloping forward so that the dorsal tip lies level with the basipterygoid processes (1) (Galton & Upchurch, 2004).

77. Orientation of the paroccipital processes in occipital view: slightly dorsolaterally directed to horizontal (0) or ventrolaterally directed (1) (Rauhut, 2003).

78. Orientation of the paroccipital processes in dorsal view: posterolateral forming a V-shaped occiput (0) or lateral forming a flat occiput (1) (Wilson, 2002).

79. Size of the post-temporal fenestra: large fenestra (0) or a small hole that is much less than half the depth of the paroccipital process (1).

80. Exit of the mid-cerebral vein: through trigeminal foramen (0) or through a separate foramen (1) (Rauhut, 2003).

81. Shape of the floor of the braincase in lateral view: relatively straight with the basal tuberae, basipterygoid processes and parasphenoid rostrum roughly aligned (0), bent with the basipterygoid processes and the parasphenoid rostrum below the level of the basioccipital condyle and the basal tuberae (1), or bent with the basal tuberae lowered below the level of the basioccipital and the parasphenoid rostrum raised above it (2) (modified from Galton 1990). Unordered.

82. Basioccipital component of basal tubera, medial component in relation to the parabasisphenoidal components: present (0), or absent (1) (Bronzati and Rauhut, 2017).

83. Length of the basipterygoid processes (from the top of the parasphenoid to the tip of the process): less than (0), or greater than (1), the height of the braincase (from the top of the parasphenoid to the top of the supraoccipital) (Benton *el al.,* 2000).

84. Basioccipital-parabasisphenoid junction on the ventral surface of the bones: straight line (0), or U/V-shaped (1) (Bronzati and Rauhut, 2017).

85. Subsellar recess: maximum width equal or greater than the dorsoventral height (0) or maximum width smaller than the dorsoventral height (1) (Bronzati and Rauhut, 2017).

86. Dorsoventral depth of the parasphenoid rostrum: much less than (0), or about equal to (1), the transverse width (Yates, 2003a).

87. Shape of jugal process of ectopterygoid: gently curved (0) or strongly recurved and hook-like (1) (Yates, 2003).

88. Pneumatic fossa on the ventral surface of the ectopterygoid: absent (0) or present (1) (Sereno *el al.,* 1996).

89. Relationship of the ectopterygoid to the pterygoid: ectopterygoid overlapping the ventral (0), or dorsal (1), surface of the pterygoid (Sereno *el al.,* 1993).

90. Position of the maxillary articular surface of the palatine: along the lateral margin of the bone (0) or at the end of a narrow anterolateral process due to the absence of the posterolateral process (1) (Wilson & Sereno, 1998).

91. Centrally located tubercle on the ventral surface of palatine: absent (0) or present (1).

92. Medial process of the pterygoid forming a hook around the basipterygoid process: absent (0), flat and blunt-ended (1), or bent upward and pointed (2) (modified from Wilson & Sereno, 1998). Ordered.

93. Length of the vomers: less than (0), or more than (1), 0.25 of the total skull length.

94. Position of jaw joint: no lower than the level of the dorsal margin of the dentary (0) or depressed well below this level (1) (Sereno, 1999).

95. Shape of upper jaws in ventral view: narrow with an acute rostral apex (0) or broad and U-shaped (1) (Wilson & Sereno, 1998).

96. Length of the external mandibular fenestra: more than (0), or less than (1), 0.1 of the length of the mandible (modified from Upchurch, 1995).

97. Caudal end of dentary tooth row medially inset with a thick lateral ridge on the dentary forming a buccal emargination: absent (0) or present (1) (Gauthier, 1986).

98. Height: length ratio of the dentary: less than (0), or greater than (1), 0.2 (modified from Benton *el al.,* 2000).

99. Orientation of the symphyseal end of the dentary: in line with the long axis of the dentary (0) or strongly curved ventrally (1) (Sereno, 1999).

100. Position of first dentary tooth: adjacent to symphysis (0) or inset one tooth’s width from the symphysis (1) (Sereno, 1999).

101. Dorsoventral expansion at the symphyseal end of the dentary: absent (0) or present (1) (Wilson & Sereno, 1998).

102. Splenial foramen: absent (0), present and enclosed (1), or present and open anteriorly (2) (Rauhut, 2003). Ordered.

103. Splenial-angular joint: flattened sutured contact (0), synovial joint surface between tongue-like process of angular fitting in groove of the splenial (1) (Sereno *el al.,* 1993).

104. A stout, triangular, medial process of the articular, behind the glenoid: present (0) or absent (1) (Yates, 2003a).

105. Length of the retroarticular process: less than (0), or greater than (1), than the depth of the mandible below the glenoid (Yates, 2003).

106. Strong medial embayment behind glenoid of the articular in dorsal view: absent (0), or present (1) (Yates & Kitching, 2003).

107. Number of premaxillary teeth: four (0) or more than four (1) (Galton, 1990).

108. Number of dentary teeth (in adults): less than 18 (0) or 18 or more (1) (modified from Wilson & Sereno, 1998).

109. Arrangement of teeth within the jaws: linearly placed, crowns not overlapping (0) or imbricated with distal side of tooth overlapping mesial side of the succeeding tooth (1).

110. Orientation of the maxillary tooth crowns: erect (0) or procumbent (1) (modified from Gauthier, 1986).

111. Orientation of the dentary tooth crowns: erect (0) or procumbent (1) (modified from Gauthier, 1986).

112. Teeth with basally constricted crowns: absent (0) or present (1) (Gauthier, 1986).

113. Tooth–tooth occlusal wear facets: absent (0) or present (1) (Wilson & Sereno, 1998).

114. Mesial and distal serrations of the mid-posterior teeth: fine and set at right angles to the margin of the tooth (0) or coarse and angled upwards at an angle of 45° to the margin of the tooth (1) (Benton *el al.,* 2000).

115. Distribution of serrations on the maxillary and mid-posterior dentary teeth: present on both the mesial and distal carinae (0), absent on the posterior carinae (1), or absent on both carinae (2) (Wilson, 2002). Unordered.

116. Long axis of the tooth crowns distally recurved: present (0) or absent (1) (Gauthier, 1986).

117. Texture of the enamel surface: entirely smooth (0), finely wrinkled in some patches (1), or extensively and coarsely wrinkled (2) (modified from Wilson & Sereno, 1998).

118. Lingual concavities of the teeth: absent (0) or present (1) (Upchurch, 1995).

119. Longitudinal labial grooves on the teeth: absent (0) or present (1) (Upchurch, 1998).

120. Distribution of the serrations along the mesial and distal carinae of the mid-posterior teeth: extend along most of the length of the crown (0) or are restricted to the upper half of the crown (1) (Yates, 2003a).

Vertebrae

121. Number of cervical vertebrae: eight or fewer (0), 9–10 (1), 12–13 (2), or more than 13 (3) (modified from Wilson & Sereno, 1998). Ordered.

122. Shallow, dorsally facing fossa on the atlantal neurapophysis bordered by a dorsally everted lateral margin: absent (0) or present (1) (Yates & Kitching, 2003).

123. Width of axial intercentrum: less than (0), or greater than (1), width of axial centrum (Sereno, 1999).

124. Position of axial prezygapophyses: on the anterolateral surface of the neural arch (0) or mounted on anteriorly projecting pedicels (1).

125. Posterior margin of the axial postzygapophyses: overhang the axial centrum (0) or are flush with the caudal face of the axial centrum (1) (Sereno, 1999).

126. Length of the axial centrum: less than (0), or at least (1), three times the height of the centrum.

127. Length of the anterior cervical centra (cervicals 3–5): no more than (0), or greater than (1), the length of the axial centrum.

128. Length of middle to posterior cervical centra (cervicals 6–8): no more than (0), or greater than (1), the length of the axial centrum.

129. Dorsal excavation of the cervical parapophyses: absent (0) or present (1) (Upchurch, 1998).

130. Lateral compression of the anterior cervical vertebrae: centra are no higher than they are wide (0) or are approximately 1.25 times higher than wide (1) (Upchurch, 1998).

131. Relative elongation of the anterior cervical centra (cervicals 3–5): lengths of the centra are less than 2.5 times the height of their anterior faces (0), lengths are 2.5–4 times the height of their anterior faces (1) or the length of at least cervical 4 or 5 exceeds 4 times the anterior centrum height (2) (modified from Sereno, 1999). Ordered.

132. Ventral keels on cranial cervical centra: present (0) or absent (1) (modified from Upchurch, 1998).

133. Height of the mid cervical neural arches: no more than (0), or greater than (1), height of the posterior centrum face.

134. Cervical epipophyses on the dorsal surface of the postzygapophyses: absent (0), or present on at least some cervical vertebrae (1).

135. Caudal ends of cranial, postaxial epipophyses: with a free pointed tip (0) or joined to the postzygapophysis along their entire length (1).

136. Shape of the epipophyses: tall ridges (0) or flattened, horizontal plates (1) (Yates, 2003a).

137. Epipophyses overhanging the rear margin of the postzygapophyses: absent (0), or present in at least some postaxial cervical vertebrae (1) (Sereno *el al.,* 1993).

138. Anterior spur-like projections on mid-cervical neural spines: absent (0) or present (1).

139. Shape of mid-cervical neural spines: less than (0), or at least (1), twice as long as high.

140. Shape of cervical rib shafts: short and posteroventrally directed (0) or longer than the length of their centra and extending parallel to cervical column (1) (Sereno, 1999).

141. Position of the base of the cervical rib shaft: level with, or higher than the ventral margin of the cervical centrum (0) or located below the ventral margin due to a ventrally extended parapophysis (1) (Wilson & Sereno, 1998).

142. Postzygodiapophyseal lamina in cervical neural arches 4–8: present (0) or absent (1) (Yates, 2003a).

143. Laminae of the cervical neural arches 4–8: well-developed tall laminae (0) or weakly developed low ridges (1) (Wilson & Sereno, 1998).

144. Shape of anterior centrum face in cervical centra: concave (0), flat (1), or convex (2) (modified from Gauthier, 1986). Ordered.

145. Ventral surface of the centra in the cervicodorsal transition: transversely rounded (0) or with longitudinal keels (1) (Rauhut, 2003).

146. Number of vertebrae between cervicodorsal transition and primordial sacral vertebrae: 15–16 (0) or no more than 14 (1) (modified from Wilson & Sereno, 1998).

147. Lateral surfaces of the dorsal centra: with at most vague, shallow depressions (0), with deep fossae that approach the midline (1), or with invasive, sharp-rimmed pleurocoels (2) (Gauthier, 1986). Ordered.

148. Oblique ridge dividing pleural fossa of cervical vertebrae: absent (0) or present (1) (Wilson & Sereno, 1998).

149. Laterally expanded tables at the midlength of the dorsal surface of the neural spines: absent in all vertebrae (0), present on the pectoral vertebrae (1) or present on the pectoral and cervical vertebrae (2) (Yates & Kitching, 2003). Ordered.

150. Dorsal centra: entirely amphicoelous to amphiplatyan (0), first two dorsals are opisthocoelous (1), or cranial half of dorsal column is opisthocoelous (2) (Wilson & Sereno, 1998). Ordered.

151. Shape of the posterior dorsal centra: relatively elongated for their size (0) strongly axially compressed for their size (1) (modified from Novas, 1993).

152. Laminae bounding triangular infradiapophyseal fossae (chonae) on dorsal neural arches: absent (0) or present (1) (Wilson, 1999).

153. Location of parapophysis in first two dorsals: at the anterior end of the centrum (0), or located at the mid-length of the centrum, within the middle chonos (1).

154. Parapophyses of the dorsal column completely shift from the centrum to the neural arch: anterior (0), or posterior (1), to the thirteenth presacral vertebra (Langer, 2004).

155. Orientation of the transverse processes of the dorsal vertebrae: most horizontally directed (0) or all upwardly directed (1) (Upchurch, 1998).

156. Contribution of the paradiapophyseal lamina to the margin of the anterior chonos in mid-dorsal vertebrae: present (0) or prevented by high placement of parapophysis (1).

157. Hyposphenes in the dorsal vertebrae: absent (0), present but less than the height of the neural canal (1), or present and equal to the height of the neural canal (2) (modified from Gauthier, 1986). Ordered.

158. Prezygodiapophyseal lamina and associated anterior triangular fossa (chonos): present on all dorsals (0) or absent in mid-dorsals (1) (Yates, 2003a).

159. Anterior centroparapophyseal lamina in dorsal vertebrae: absent (0) or present (1) (Wilson 2002).

160. Prezygoparapophyseal lamina in dorsal vertebrae: absent (0) or present (1).

161. Accessory lamina dividing posterior chonos from postzygapophysis: absent (0) or present (1).

162. Lateral pneumatic fenestra in middle chonos of middle and posterior dorsal vertebrae opening into neural cavity: absent (0) or present (1) (Wilson & Sereno, 1998).

163. Separation of lateral surfaces of anterior dorsal neural arches under transverse processes: widely spaced (0) or only separated by a thin midline septum (1) (Upchurch *el al.,* 2004).

164. Height of dorsal neural arches, from neurocentral suture to level of zygapophyseal facets: much less than (0), or subequal to or greater than (1), height of centrum.

165. Form of anterior surface of neural arch: simple centroprezygopophyseal ridge (0) or broad anteriorly facing surface bounded laterally by centroprezygopophyseal lamina (1) (Bonaparte, 1999).

166. Shape of posterior dorsal neural canal: subcircular (0) or slit-shaped (1) (Wilson & Sereno, 1998).

167. Height of middle dorsal neural spines: less than the length of the base (0), higher than the length of the base but less than 1.5 times the length of the base (1) or greater than 1.5 times the length of the base (2) (modified from Bonaparte, 1986). Ordered.

168. Shape of anterior dorsal neural spines: lateral margins parallel in anterior view (0) or transversely expanding towards dorsal end (1).

169. Cross-sectional shape of dorsal neural spines: transversely compressed (0), broad and triangular (1), or square-shaped in posterior vertebrae (2) (modified from Bonaparte, 1986).

170. Spinodiapophyseal lamina on dorsal vertebrae: absent (0), present and separated from spinopostzygapophyseal lamina (1) or present and joining spinopostzygapophyseal lamina to create a composite posterolateral spinal lamina (Wilson & Sereno, 1998).

171. Well-developed, sheet-like suprapostzygapophyseal laminae: absent (0), present on at least the caudal dorsal vertebrae (1) (Bonaparte, 1986).

172. Shape of the spinopostzygapophyseal lamina in middle and posterior dorsal vertebrae: singular (0) or bifurcated at its distal end (1) (Wilson, 2002).

173. Shape of posterior margin of middle dorsal neural spines in lateral view: approximately straight (0) or concave with a projecting posterodorsal corner (1) (Yates, 2003b).

174. Transversely expanded plate-like summits of posterior dorsal neural spines: absent (0) or present (1) (Novas, 1993).

175. Last presacral rib: free (0) or fused to vertebra (1).

176. Sacral rib much narrower than the transverse process of the first primordial sacral vertebra (and dorsosacral if present) in dorsal view: absent (0) or present (1) (Yates & Kitching 2003).

177. Number of dorsosacral vertebrae: none (0), one (1), or two (2) (modified from Gauthier, 1986). Ordered.

178. Caudosacral vertebra: absent (0) or present (1) (Galton & Upchurch, 2004).

179. Shape of the iliac articular facets of the first primordial sacral rib: singular (0) or divided into dorsal and ventral facets separated by a non-articulating gap (1).

180. Depth of the iliac articular surface of the primordial sacrals: less than (0), or greater than (1), 0.75 of the depth of the ilium (modified from Novas, 1992 by Yates, 2007).

181. Sacral ribs contributing to the rim of the acetabulum: absent (0) or present (1) (Wilson, 2002).

182. Posterior and anterior expansion of the transverse processes of the first and second primordial sacral vertebrae, respectively, partly roofing the intercostal space: absent (0) or present (1) (Langer, 2004).

183. Length of first caudal centrum: greater than its height (0), or less than its height (1), or highly compressed (dorsoventral height at least twice anteroposterior length) (2) (modified from Yates, 2003a by McPhee & Choiniere, 2017). Ordered.

184. Length of base of the proximal caudal neural spines: less than (0), or greater than (1), half the length of the neural arch (Gauthier, 1986).

185. Position of postzygapophyses in proximal caudal vertebrae: protruding with an interpostzygapophyseal notch visible in dorsal view (0) or placed on either side of the caudal end of the base of the neural spine without any interpostzygapophyseal notch (1) (Yates, 2003a).

186. A hyposphenal ridge on caudal vertebrae: absent (0) or present (1) (Upchurch, 1995).

187. Depth of the bases of the proximal caudal transverse processes: shallow, restricted to the neural arches (0), deep, extending from the centrum to the neural arch (1) (Upchurch, 1998).

188. Position of last caudal vertebra with a protruding transverse process: distal (0), or proximal (1), to caudal 16 (Wilson, 2002).

189. Orientation of posterior margin of proximal caudal neural spines: sloping posterodorsally (0) or vertical (1) (Novas, 1992).

190. Longitudinal ventral sulcus on proximal and middle caudal vertebrae: present (0) or absent (1) (modified from Upchurch , 1995).

191. Length of midcaudal centra: greater than (0), or less than (1), twice the height of their anterior faces (Yates, 2003a).

192. Cross-sectional shape of the distal caudal centra: oval with rounded lateral and ventral sides (0) or square-shaped with flattened lateral and ventral sides (1).

193. Length of distal caudal prezygapophyses: short, not overlapping the preceding centrum by more than a quarter (0) or long and overlapping the preceding the centrum by more than a quarter (Gauthier, 1986).

194. Shape of the terminal caudal vertebrae: unfused, size decreasing toward tip (0) or expanded and fused to form a club-shaped tail (1) (Upchurch, 1995).

195. Length of the longest chevron: is less than (0), or greater than (1), twice the length of the preceding centrum (modified from Yates, 2003a).

196. Anteroventral process on distal chevrons: absent (0) or present (1) (Upchurch, 1995).

197. Mid-caudal chevrons with a ventral slit: absent (0) or present (1) (Upchurch, 1995).

Appendicular

198. Longitudinal ridge on the dorsal surface of the sternal plate: absent (0) or present (1) (Upchurch, 1998).

199. Craniocaudal length of the acromion process of the scapula: less than (0), or greater than (1), 1.5 times the minimum width of the scapula blade (Wilson & Sereno, 1998).

200. Minimum width of the scapula: less than (0), or greater than (1), 20% of its length (Gauthier, 1986).

201. Caudal margin of the acromion process of the scapula: rises from the blade at angle that is less than (0), or greater than (1), 65° from the long axis of the scapula, at its steepest point (modified from Novas, 1992).

202. Width of dorsal expansion of the scapula: less than (0), or equal to (1), the width of the ventral end of the scapula (Pol and Powell, 2007).

203. Flat caudoventrally facing surface on the coracoid between glenoid and coracoid tubercle: absent (0) or present (1) (Yates & Kitching, 2003).

204. Coracoid tubercle: present (0) or absent (1) (modified from Pérez-Moreno *el al.,* 1994).

205. Length of the humerus: less than 55% (0), 55–65% (1), 65–70% (2), or more than 70% (3), of the length of the femur (modified from Gauthier, 1986). Ordered.

206. Shape of the deltopectoral crest: subtriangular (0) or subrectangular (1) (Gauthier, 1986).

207. Length of the deltopectoral crest of the humerus: less than 30% (0), 30–50% (1), or greater than 50% (2), of the length of the humerus (modified from Sereno *el al.,* 1993). Ordered.

208. Shape of the anterolateral margin of the deltopectoral crest of the humerus: straight (0) or strongly sinuous (1) (Yates, 2003a).

209. Rugose pit centrally located on the lateral surface of the deltopectoral crest: absent (0) or present (1).

210. Well-defined fossa on the distal flexor surface of the humerus: present (0) or absent (1) (Yates & Kitching, 2003).

211. Transverse width of the distal humerus: is less than (0), or greater than (1), 33% of the length of the humerus (Langer, 2004).

212. Shape of the entepicondyle of the distal humerus: rounded process (0) or with a flat distomedially facing surface bounded by a sharp proximal margin (1).

213. Length of the radius: greater than (0), or less than (1), 80% of the humerus (Langer, 2004).

214. Deep radial fossa, bounded by an anterolateral process, on proximal ulna: absent (0), or present but poorly defined (1), or a well-defined recess, deeper than the transverse width of the anterior end of the anterior process (2) (modified from Wilson & Sereno, 1998 by McPhee & Choiniere, 2017). Ordered.

215. Olecranon process on proximal ulna: present (0) or absent (1) (Wilson & Sereno, 1998).

216. Maximum linear dimensions of the ulnare and radiale: exceed that of at least one of the first three distal carpals (0) or are less than any of the distal carpals (1) (Yates, 2003a).

217. Transverse width of the first distal carpal: less than (0), or greater than (1), 120% of the transverse width of the second distal carpal (Sereno, 1999).

218. Sulcus across the medial end of the first distal carpal: absent (0) or present (1).

219. Lateral end of first distal carpal: abuts (0), or overlaps (1), second distal carpal (Yates, 2003a).

220. Second distal carpal: does (0), or does not (1), completely cover the proximal end of the second metacarpal (Yates & Kitching, 2003).

221. Ossification of the fifth distal carpal: present (0) or absent (1).

222. Length of the manus: less than 38% (0), 38–45% (1), or greater than 45% (2), of the humerus + radius (modified from Sereno *el al.,* 1993). Ordered.

223. Shape of metacarpus: flattened to gently curved and spreading (0) or a colonnade of subparallel metacarpals tightly curved into a U-shape (1) (Wilson & Sereno, 1998).

224. Proximal width of first metacarpal: less than (0), or greater than (1), the proximal width of the second metacarpal (modified from Gauthier, 1986).

225. Minimum transverse shaft width of first metacarpal: less than (0), or greater than (1), twice the minimum transverse shaft width of second metacarpal.

226. Proximal end of first metacarpal: flush with other metacarpals (0) or inset into the carpus (1) (Sereno, 1999).

227. Shape of the first metacarpal: proximal width less than 65% (0), 65–80% (1), 80–100% (2), or greater than 100% (3), of its length (modified from Sereno, 1999). Ordered.

228. Strong asymmetry in the lateral and medial distal condyles of the first metacarpal: absent (0) or present (1) (Gauthier, 1986).

229. Deep distal extensor pits on the second and third metacarpals: absent (0) or present (1) (Novas, 1993).

230. Shape of the distal ends of second and third metacarpals: subrectangular in distal view (0) or trapezoidal with flexor rims of distal collateral ligament pits flaring beyond extensor rims (1).

231. Shape of the fifth metacarpal: longer than wide at the proximal end with a flat proximal surface (0) or close to as wide as it is long with a strongly convex proximal articulation surface (1) (Yates, 2003a).

232. Length of the fifth metacarpal: less than (0), or greater than (1), 75% of the length of the third metacarpal (Upchurch, 1998).

233. Length of manual digit one: less than (0), or greater than (1), the length of manual digit two (Yates, 2003a).

234. Ventrolateral twisting of the transverse axis of the distal end of the first phalanx of manual digit one relative to its proximal end: absent (0), present but much less than 60° (1), or 60° (2) (Sereno, 1999). Ordered.

235. Length of the first phalanx of manual digit one: less than (0), or greater than (1), the length of the first metacarpal (Gauthier, 1986).

236. Shape of the proximal articular surface of the first phalanx of manual digit one: rounded (0) or with an embayment on the medial side (1) (modified from Sereno, 1999).

237. Shape of the first phalanx of manual digit one: elongate and subcylindrical (0) or strongly proximodistally compressed and wedge-shaped (1) (Wilson, 2002).

238. Length of the penultimate phalanx of manual digit two: less than (0), or greater than (1), the length of the second metacarpal (Rauhut, 2003).

239. Length of the penultimate phalanx of manual digit three: less than (0), or greater than (1), the length of the third metacarpal (Rauhut, 2003).

240. Shape of non-terminal phalanges of manual digits two and three: longer than wide (0) or as long as wide (1) (Yates, 2003a).

241. Shape of the unguals of manual digits two and three: straight (0), or strongly curved with tips projecting well below flexor margin of proximal articular surface (1) (Sereno *el al.,* 1993).

242. Length of the ungual of manual digit two: greater than the length of the ungual of manual digit one (0), 75–100% of the ungual of manual digit one (1), less than 75% of the ungual of manual digit one (2), or the ungual of manual digit two is absent (3) (modified from Gauthier, 1986). Ordered.

243. Phalangeal formula of manual digits two and three: three and four, respectively (0), or with at least one phalanx missing from each digit (1) (modified from Wilson & Sereno 1998).

244. Phalangeal formula of manual digits four and five: greater than (0), or less than (1), 2–0, respectively (Gauthier ,1986).

245. Strongly convex dorsal margin of the ilium: absent (0) or present (1) (Gauthier, 1986).

246. Cranial extent of preacetabular process of ilium: does not (0), or does (1), project further forward than cranial end of the pubic peduncle (Yates, 2003a).

247. Shape of the preacetabular process: blunt and rectangular (0) or with a pointed, projecting cranioventral corner and a rounded dorsum (1) (modified from Sereno, 1999).

248. Depth of the preacetabular process of the ilium: much less than (0), or subequal to (1), the depth of the ilium above the acetabulum (modified from Gauthier, 1986).

249. Length of preacetabular process of the ilium: less than (0), or greater than (1), twice its depth.

250. Buttress between preacetabular process and the supraacetabular crest of the ilium: present (0) or absent (1) (Gauthier, 1986).

251. Medial wall of acetabulum: fully closing acetabulum with a triangular ventral process between the pubic and ischial peduncles (0), partially open acetabulum with a straight ventral margin between the peduncles (1), partially open acetabulum with a concave ventral margin between the peduncles (2), or fully open acetabulum with medial ventral margin closely approximating lateral rim of acetabulum (3) (modified from Gauthier 1986). Ordered.

252. Length of the pubic peduncle of the ilium: less than (0), or greater than (1), twice the craniocaudal width of its distal end (Sereno, 1999).

253. Caudally projecting ‘heel’ at the distal end of the ischial peduncle: absent (0) or present (1) (Yates, 2003b).

254. Length of the ischial peduncle of the ilium: similar to pubic peduncle (0), much shorter than pubic peduncle (1), or virtually absent so that the chord connecting the distal end of the pubic peduncle with the ischial articular surface contacts the postacetabular process (2) (Upchurch *el al.,* 2004). Ordered.

255. Length of the postacetabular process of the ilium: between 40% and 100% of the distance between the pubic and ischial peduncles (0), less than 40% of this distance (1), or more than 100% of this distance (2). Unordered.

256. Well developed brevis fossa with sharp margins on the ventral surface of the postacetabular process of the ilium: absent (0) or present (1) (Gauthier, 1986).

257. Anterior end of ventrolateral ridge bounding brevis fossa: not connected to (0), or joining (1) supracetabular crest (1).

258. Shape of the caudal margin of the postacetabular process of the ilium: rounded to bluntly pointed (0), square ended (1), or with a pointed ventral corner and a rounded caudodorsal margin (2) (Yates, 2003b). Unordered.

259. Width of the conjoined pubes: less than (0), or greater than (1), 75% of their length (Cooper, 1984).

260. Pubic tubercle on the lateral surface of the proximal pubis: present (0) or absent (1) (Yates, 2003a).

261. Proximal anterior profile of pubis: anterior margin of pubic apron smoothly confluent with anterior margin of iliac pedicel (0) or iliac pedicel set anterior to the pubic apron creating a prominent inflection in the proximal anterior profile of the pubis (1).

262. Minimum transverse width of the pubic apron: much more than (0), or less than (1), 40% of the width across the iliac peduncles of the ilium.

263. Position of the obturator foramen of the pubis: at least partially occluded by the iliac pedicel (0), or completely visible (1), in anterior view (Galton & Upchurch, 2004).

264. Lateral margins of the pubic apron in anterior view: straight (0) or concave (1) (Yates & Kitching, 2003).

265. Orientation of distal third of the blades of the pubic apron: confluent with the proximal part of the pubic apron (0) or twisted posterolaterally relative to proximal section so that the anterior surface turns to face laterally (1) (Langer, 2004).

266. Orientation of the entire blades of the pubic apron: transverse (0) or twisted posteromedially (1) (Wilson & Sereno, 1998).

267. Craniocaudal expansion of the distal pubis: absent (0), less than 15% (1), or greater than 15% (2), of the length of the pubis (modified from Gauthier, 1986). Ordered.

268. Notch separating posteroventral end of the ischial obturator plate from the ischial shaft: present (0) or absent (1) (Rauhut, 2003).

269. Elongate interischial fenestra: absent (0) or present (1) (Yates, 2003b).

270. Longitudinal dorsolateral sulcus on proximal ischium: absent (0) or present (1) (Yates, 2003a).

271. Shape of distal ischium: broad and plate-like, not distinct from obturator region (0) or with a discrete rod-like distal shaft (1).

272. Length of ischium: less than (0) or greater than (1) that of the pubis (Salgado, Coria & Calvo, 1997).

273. Ischial component of acetabular rim: larger than (0), or equal to (1), the pubic component (Galton & Upchurch, 2004).

274. Shape of the transverse section of the ischial shaft: ovoid to subrectangular (0) or triangular (1) (Sereno, 1999).

275. Orientation of the long axes of the transverse section of the distal ischia: meet at an angle (0) or are coplanar (1) (Wilson & Sereno, 1998).

276. Depth of the transverse section of the ischial shaft: much less than (0) at least as great as (1), the transverse width of the section (Wilson & Sereno, 1998).

277. Distal ischial expansion: absent (0) or present (1) (Holtz, 1994).

278. Transverse width of the conjoined distal ischial expansions: greater than (0), or less than (1), their sagittal depth (Yates, 2003a).

279. Length of the hindlimb: greater than (0), or less than (1), the length of the trunk (Gauthier, 1986).

280. Longitudinal axis of the femur in lateral view: strongly bent with an offset between the proximal and distal axes greater than 15° (0), weakly bent with an offset of less than 10° (1), or straight (2) (Cooper, 1984). Ordered.

281. Shape of the cross-section of the mid-shaft of the femur: subcircular (0) or strongly elliptical (eccentricity > 1.5) with the long axis orientated mediolaterally (1) (Wilson & Sereno, 1998).

282. Angle between the long axis of the femoral head and the transverse axis of the distal femur: about 30° (0) or close to 0° (1) (Carrano, 2000).

283. Shape of femoral head: roughly rectangular in profile with a sharp medial distal corner (0) or roughly hemispherical with no sharp medial distal corner (1). This character only applies to taxa with a medially, or anteromedially protruding femoral head. It does not apply to outgroup taxa (*Euparkeria* or Crurotarsi) with proximally directed femoral heads and is coded as unknown in these taxa.

284. Posterior proximal tubercle on femur: well developed (0) or indistinct to absent (1) (Novas, 1996).

285. Shape of the lesser trochanter: small rounded tubercle (0), proximodistally orientated, elongate ridge (1), or absent (2) (modified from Gauthier, 1986). Unordered.

286. Position of proximal tip of lesser trochanter: level with (0), or distal to (1), the femoral head (Galton & Upchurch, 2004).

287. Projection of the lesser trochanter: just a scar upon the femoral surface (0) or a raised process (1).

288. Transverse ridge extending laterally from the lesser trochanter: absent (0) or present (1) (Rowe, 1989).

289. Height of the lesser trochanter in cross section: less than (0), or at least as high as (1), basal width (modified from Galton, 1990).

290. Position of the lesser trochanter: near the centre of the anterior face (0), or close to the lateral margin (1), of the femoral shaft in anterior view.

291. Visibility of the lesser trochanter in posterior view: not visible (0) or visible (1) (Galton & Upchurch, 2004).

292. Height of the fourth trochanter: low rugose ridge (0) or a tall crest (1) (Gauthier, 1986).

293. Position of the fourth trochanter along the length of the femur: in the proximal half (0) or straddling the midpoint (1) (Galton, 1990).

294. Symmetry of the profile of the fourth trochanter of the femur: subsymmetrical without a sharp distal corner (0) or asymmetrical with a steeper distal slope than the proximal slope and a distinct distal corner (1) or symmetrical, almost rectangular in lateral view with proximal and distal corners approaching an angle of 90 degrees (2) (modified from Langer, 2004).

295. Shape of the profile of the fourth trochanter of the femur: rounded (0) or subrectangular (1).

296. Position of fourth trochanter along the mediolateral axis of the femur: centrally located (0) on the medial margin (1) (Galton, 1990).

297. Extensor depression on anterior surface of the distal end of the femur: absent (0) or present (1) (Molnar, Kurzanov & Dong Zhiming, 1990).

298. Size of the medial condyle of the distal femur: subequal to (0), or larger than (1), the fibular + lateral condyles (modified from Wilson, 2002).

299. Tibia : femur length ratio: greater than 1.0 (0), between 0.6 and 1.0 (1) or less than 0.6 (2) (modified from Gauthier, 1986). Ordered.

300. Orientation of cnemial crest: projects anteriorly to anterolaterally (0) or projecting laterally (1) (Wilson & Sereno, 1998).

301. Paramarginal ridge on lateral surface of cnemial crest: absent (0) or present (1).

302. Position of the tallest point of the cnemial crest: close to the proximal end of the crest (0) or about half-way along the length of the crest, creating an anterodorsally sloping proximal margin of the crest (1).

303. Proximal end of tibia with a flange of bone that contacts the fibula: absent (0) or present (1) (Gauthier, 1986).

304. Position of the posterior end of the fibular condyle on the proximal articular surface tibia: anterior to (0) or level with (1), the posterior margin of proximal articular surface.

305. Shape of the proximal articular surface of the tibia: ovoid, anteroposteriorly longer than transversely wide (0) or subcircular and as wide transversely as anteroposteriorly long (1) (Wilson & Sereno, 1998).

306. Transverse width of the distal tibia: subequal to (0), or greater than (1), its craniocaudal length (Gauthier, 1986).

307. Anteroposterior width of the lateral side of the distal articular surface of the tibia: as wide (0), or narrower than (1), the anteroposterior width of the medial side.

308. Relationship of the posterolateral process of the distal end of the tibia with the fibula: not flaring laterally and not making significant contact with the fibula (0) or flaring laterally and backing the fibula (1).

309. Shape of the distal articular end of the tibia in distal view: ovoid (0) or subrectangular (1).

310. Shape of the anteromedial corner of the distal articular surface of the tibia: forming a right angle (0) or forming an acute angle (1) (Langer, 2004).

311. Position of the lateral margin of descending caudoventral process of the distal end of the tibia: protrudes laterally at least as far as (0), or set well back from (1), the craniolateral corner of the distal tibia (Wilson & Sereno, 1998).

312. A triangular rugose area on the medial side of the fibula: absent (0) or present (1) (Wilson & Sereno, 1998).

313. Transverse width of the midshaft of the fibula: greater than 0.75 (0), between 0.5 and 0.75 (1), or less than 0.5 (2), of the transverse width of the midshaft of the tibia (Langer, 2004). Ordered.

314. Position of fibula trochanter: on anterior surface of fibula (0), laterally facing (1), or anteriorly facing but with strong lateral bulge (2) (modified from Wilson & Sereno, 1998).

315. Depth of the medial end of the astragalar body in cranial view: roughly equal to the lateral end (0) or much shallower creating a wedge-shaped astragalar body (1) (Wilson & Sereno, 1998).

316. Shape of the posteromedial margin of the astragalus in dorsal view: forming a moderately sharp corner of a subrectangular astragalus (0) or evenly rounded without formation of a caudomedial corner (1) (Wilson & Sereno, 1998).

317. Dorsally facing horizontal shelf forming part of the fibular facet of the astragalus: present (0) or absent with a largely vertical fibular facet (1) (Sereno, 1999).

318. Pyramidal dorsal process on the posteromedial corner of the astragalus: absent (0) or present (1).

319. Shape of the ascending process of the astragalus: anteroposteriorly deeper than transversely wide (0) or transversely wider than anteroposteriorly deep (1).

320. Posterior extent of ascending process of the astragalus: well anterior to (0), or close to the posterior margin of (1), the astragalus (Wilson and Sereno, 1998).

321. Sharp medial margin around the depression posterior to the ascending process of the astragalus: absent (0) or present (1) (Novas 1996).

322. Buttress dividing posterior fossa of astragalus and supporting ascending process: absent (0) or present (1) (Wilson & Sereno, 1998).

323. Vascular foramina set in a fossa at the base of the ascending process of the astragalus: present (0) or absent (1) (Wilson & Sereno, 1998).

324. Transverse width of the calcaneum: greater than (0), or less than (1), 30% of the transverse width of the astragalus (Yates & Kitching, 2003).

325. Lateral surface of calcaneum: simple (0) or with a fossa (1).

326. Medial peg of calcaneum fitting into astragalus: present, even if rudimentary (0) or absent (1) (Sereno *el al.,* 1993).

327. Calcaneal tuber: large and well developed (0) or highly reduced to absent (1).

328. Shape of posteromedial heel of distal tarsal four (lateral distal tarsal): proximodistally deepest part of the bone (0) or no deeper than the rest of the bone (1) (Sereno *el al.,* 1993).

329. Shape of posteromedial process of distal tarsal four in proximal view: rounded (0) or pointed (1) (Langer, 2004).

330. Ossified distal tarsals: present (0) or absent (1) (Gauthier, 1986).

331. Proximal width of the first metatarsal: is less than (0), or at least as great as (1), the proximal width of the second metatarsal (modified from Wilson & Sereno, 1998).

332. Orientation of proximal articular surface of metatarsal one: horizontal (0) or sloping proximolaterally relative to the long axis of the bone (1) (Wilson, 2002).

333. Orientation of the transverse axis of the distal end of metatarsal one: horizontal (0) or angled proximomedially (1) (Wilson, 2002).

334. Shape of the medial margin of the proximal surface of the second metatarsal: straight (0) or concave (1) (modified from Sereno, 1999).

335. Shape of the lateral margin of the proximal surface of the second metatarsal: straight (0) or concave (1) (modified from Sereno, 1999).

336. Length of the third metatarsal: greater than (0), or less than (1), 40% of the length of the tibia (Gauthier, 1986).

337. Minimum transverse shaft diameters of third and fourth metatarsals: greater than (0), or less than (1), 60% of the minimum tansverse shaft diameter of the second metatarsal (Wilson & Sereno, 1998).

338. Transverse width of the proximal end of the fourth metatarsal: less than (0), or at least (1), twice the anteroposterior depth of the proximal end (modified from Sereno, 1999).

339. Transverse width of the proximal end of the fifth metatarsal: less than 25% (0), between 30% and 49% (1), or greater than 50% (2), of the length of the fifth metatarsal (modified from Sereno, 1999). Ordered.

340. Transverse width of distal articular surface of metatarsal four in distal view: greater (0), or less than (1), anteroposterior depth (Sereno, 1999).

341. Pedal digit five: reduced, non-weight bearing (0) or large (fifth metatarsal at least 70% of fourth metatarsal), robust and weight bearing (1) (Wilson & Sereno, 1998).

342. Length of non-terminal pedal phalanges: all longer than wide (0), proximalmost phalanges longer than wide while more distal phalanges are as wide as long (1), or all non-terminal phalanges are as wide, if not wider, than long (2) modified from Wilson & Sereno, 1998). Ordered.

343. Length of the first phalanx of pedal digit one: greater than (0), or less than (1), the length of the ungual of pedal digit one (Yates & Kitching, 2003).

344. Length of the ungual of pedal digit one: less than at least some non-terminal phalanges (0) or longer than all non-terminal phalanges (1) or longer than first metatarsal (2). Ordered. (modified from Yates, 2007).

345. Shape of the ungual of pedal digit one: shallow, pointed, with convex sides and a broad ventral surface (0) or deep, abruptly tapering, with flattened sides and a narrow ventral surface (1) (Wilson & Sereno, 1998).

346. Shape of proximal articular surface of pedal unguals: proximally facing, visible on medial and lateral sides (0) or proximomedially facing and visible only in medial view, causing medial deflection of pedal unguals in articulation (1) (Wilson & Sereno, 1998).

347. Penultimate phalanges of pedal digits two and three: well-developed (0) or reduced disc-shaped elements if they are ossified at all (1) (Wilson & Sereno, 1998).

348. Shape of the unguals of pedal digits two and three: dorsoventrally deep with a proximal articulating surface that is at least as deep as it is wide (0) or dorsoventrally flattened with a proximal articulating surface that is wider than deep (1) (Wilson & Sereno, 1998).

349. Length of the ungual of pedal digit two: greater than (0), between 90% and 100% of (1), or less than 90% of (2), the length of the ungual of pedal digit one (modified from Gauthier, 1986). Ordered.

350. Size of the ungual of pedal digit three: greater than (0), or less than (1), 85% of the ungual of pedal digit two in all linear dimensions (Yates, 2003a).

351. Number of phalanges in pedal digit four: four (0) or fewer than four (1) (Gauthier 1986).

352. Phalanges of pedal digit five: present (0) or absent (1) (Gauthier, 1986).

353. Femoral length: less than 200 mm (0), between 200 and 399 mm (1), between 400 and 599 mm (2), between 600 and 799 mm (3), between 800 and 1000 mm (4), or greater than 1000 mm (modified from Yates, 2004). Ordered.

354. Lateral extent of ventrolateral flange on plantar surface of MT II in proximal aspect: similar in development to ventromedial flange (0), well−developed, extending further laterally than ventromedial flange extends medially (1) (Smith and Pol, 2007).

355. Distal articular surface of astragalus: relatively flat or weakly convex (0), extremely convex and “roller−shaped” (1) (Smith and Pol, 2007).

356. Distal surface of tibiofibular crest: as deep anteroposteriorly as wide mediolaterally or deeper (0), wider mediolaterally than deep anteroposteriorly (1) (Smith and Pol, 2007).

357. Well−developed facet on proximolateral corner of plantar ventrolateral flange of

MT II for articulation with medial distal tarsal: absent (0), present (1) (Smith and Pol, 2007).

358. Proximal outline of metatarsal III: subtriangular with acute or rounded posterior border (0), subtrapezoidal, with posterior border broadly exposed in plantar view (1).

359. Angle formed by the anterior and anteromedial borders of metatarsal IV: obtuse (0), right angle, or acute (1) (Smith and Pol, 2007).

360. Well−developed tibiofibular crest on distal femur: absent (0), present (1) (Smith and Pol, 2007).

361. Shaft of metatarsal I: closely appressed to metatarsal II throughout its length (0), or only closely appressed proximally, with a space between metatarsals I and II distally (1) (Smith and Pol, 2007).

362. Posterior margin of astragalus: straight (0), convex (1) (Otero & Pol, 2013).

363. Ventromedial ridge of scapula: absent (0), present (1) (Otero & Pol, 2013).

364. Mediolateral surface of distal astragalus straight (0), concave (1), or convex (2). (Otero & Pol, 2013). Unordered.

365. Anterior fossa on the proximal region of the pubic apron: absent (0), present (1). (Apaldetti *el al.,* 2012).

366. Proximal end of the tibia with a transverse/anteroposterior length ratio: narrow

(ratio less than 0.7) (0), or broad (more than 0.7) (1). (Apaldetti *el al.,* 2012).

367. Caudodistal tubercle of the radius: absent (0), present (1). (Otero et al., 2015).

368. Biceps tubercle of the radius: absent (0), present (1). (Otero et al., 2015).

369. Ventromedial margin of first metacarpal: poorly concave (0), deeply concave (1). (Otero et al., 2015).

370. Length of first phalanx of manual digit 1: much greater than (0), subequal or equal to (1), or much less than (2) its mediolateral width at proximal end. (Otero et al., 2015).

371. Muscle origin areas (*Mm. flexor tibialis* and *iliotibialis*) on the posterior portion of the postacetabular process of the ilium: smooth or as a rectangular rugosity (0), strong trapezoidal rugosity extended along the whole height of the posterior third of the process (1). (Ezcurra, 2010).

372. Supraacetabular crest of ilium: not extended along the pubic peduncle or only at the base of the peduncle (0), extended along the pubic peduncle as a faint ridge (1), extended along the entire pubic peduncle and contacts the distal end as a well developed crest (2). (Ezcurra, 2010).

373. Subnarial gap (i.e. posterior part of premaxillary alveolar margin edentelous, resulting in an interruption of the upper tooth row): absent (0), present (1). (Gauthier 1986).

374. Alveolar margin of anterior-most maxilla: relatively straight or slightly convex (0), strongly but gradually upturned from an extension of more than three teeth along the alveolar margin and orienting the first maxillary alveolus anteroventrally (1), sharply mediodorsally upturned in the anterior-most tip of the maxilla and orienting the first maxillary alveolus anteroventrally (2). (Rowe 1989, Tykoski 1998, Ezcurra & Novas 2007b).

375. Anterior margin of maxillary antorbital fossa: rounded or pointed (0), squared (1). (Rauhut 2003).

376. Dorsoventrally compressed ridge on lateral surface of maxilla, forming the ventral border of the antorbital fossa (alveolar ridge): absent (0), present (1). (Rowe & Gauthier 1990).

377. Exposition of the lacrimal antorbital fossa in lateral view: lateral lamina of bone covering most of the bone, with antorbital fossa exposed only at the distal end of the vertical process (0), lateral lamina of bone only interrupting the fossa near the proximal end of the ventral ramus and ventrally restricted to posterior margin of the ventral ramus, with antorbital fossa laterally exposed along most of the lacrimal (1). (Modified from Ezcurra & Novas 2007b).

378. Medial distal condyle of metacarpal I: of the same size (0) or dorsoventrally smaller (1) than the lateral distal condyle. (Ezcurra, 2010).

379. Metacarpals IV and V ventral to metacarpals I-III: absent (0), present (1). (Sereno 1993)

380. Supraacetabular crest of ilium: present as a weakly developed ridge (0), present as a well developed raised shelf (1), flares lateroventrally to form a hood-like overhang that hides anterodorsal half of acetabulum in lateral view (2). (Langer 2004, Tykoski 2005).

381. Iliac blade in dorsal view: straight or slightly laterally curved along the whole of its anteroposterior extension (0), strongly laterally curved, with a deeply concave lateral border (1). (Ezcurra, 2010).

382. Pubic shaft: posteriorly bowed (0), nearly straight (1). (Sereno 1999, Ezcurra & Novas 2007b).

383. Femoral head: weakly developed and slightly inturned, oriented at more than 120◦ from the main axis of the femoral shaft (0), strongly inturned, oriented at less than 120◦ from the main axis of the femoral head, and distinctively separated from the shaft by a well developed femoral neck (1). (Ezcurra 2006).

384. Posterolateral corner of the distal end of the tibia: convex (0), concave (1). (Ezcurra 2006)

385. Ungual of pedal digit II: shorter or equal in length to (0), or longer than (1), pedal phalanx II-2. (Ezcurra, 2010).

386. Distal outline of ischium: roughly semicircular (0), sub-triangular (1). (Sereno 1999).

387. Orientation of the pubic shaft: anteroventral (0), ventral, almost perpendicular to the longitudinal axis of the ilium, or slightly posteroventral (1), strongly posteroventral, with the pubic shaft parallel to the ischial shaft (2). (Modified from Sereno 1986 and Novas 1992).

388. Femoral distal transverse width: equal or lesser (0), greater (1) than 1·4 times its largest anteroposterior depth across the fibular condyle. (Novas et al. 2011).

389. Astragalus with medial condyle anteropos- terior depth: less (0), equal or more (1) than 1.6 times the depth of the lateral condyle. (Novas et al. 2011)

390. Prezygadiapophyseal laminae on anterior caudal vertebrae: absent (0), present (1). (McPhee et al. 2015).

391. ‘Weaponized’ dermal spikes on tail: absent (0), present (1). (McPhee et al. 2015).

392. Shape of the humeral head: weakly developed, rounded in anterior-posterior view but minimally expanded perpendicular to the latter axis (0), flat in anterior-posterior view with only a slightly expanded lateral component (1), domed, being convex/hemispherical in anterior-posterior view with a strong lateral incursion onto the humeral shaft (2). (McPhee et al., 2015).

393. Size of first metatarsal: maximum proximal breadth less than 0.4 times its proximodistal length (0), maximum proximal breadth between 0.4 and 0.7 times its proximodistal length (1), maximum proximal breadth greater than 0.7times its proximodistal length (2). (McPhee et al., 2015). Ordered.

394. Laminae/ridges extending from the basipterygoid process onto the parasphenoid rostrum: extend parallel until they fade into the ventral margin of the cultriform process (0), or converge anteromedially on the ventral surface of the cultriform process (1) (Bronzati and Rauhut, 2017).

395. Angle between basipterygoid process and cultriform process of the parabasisphenoid: < 90° (0), 90° (1), or > 90° (2) (Bronzati and Rauhut, 2017). Unordered.

396. Length of the basisphenoid (from the basipterygoid process to the basisphenoidal component of the basal tubera) in relation to the length of the basioccipital (from the basioccipital component of the basal tubera to posterior limit of the condyle): longer or equal (0), or shorter (1). (Bronzati and Rauhut, 2017).

397. Notch in the posterodorsal margin of the lateral portion of the parabasisphenoid: absent (0), or present (1). (Bronzati and Rauhut, 2017).

398. Number of foramina in the otoccipital between the exoccipital pillar (excluding the foramina for the hypoglossal nerve) posteriorly and fenestra ovalis anteriorly: one (0), or two (1). (Bronzati and Rauhut, 2017).

399. Unossified gap between the basioccipital and basisphenoidal component of the basal tubera and ventral ramus of the opistothic: absent (0), or present (1). (Bronzati and Rauhut, 2017).

400. Otosphenoidal crest: low and not projecting posterolaterally (i.e. does not cover the fenestra ovalis with the braincase in lateral view) (0), or developed as a lamina projecting posterolaterally (i.e. cover the fenestra ovalis with the braincase in lateral view) (1). (Bronzati et al., 2018).

401. Frontal, anteroposterior length: approximately twice (0), or less than minimum transverse breadth (1). (Wilson, 2002).

402. Parietal, distance separating supratemporal fenestrae: less than (0), or twice the long axis of supratemporal fenestra (1). (Wilson, 2002).

403. Supratemporal region, anteroposterior length: temporal bar longer (0), or shorter anteroposteriorly than transversely (1). (Wilson, 2002).

404. Orientation of the anterior- to-middle cervical postzygapophyses: planar (minimally offset) with respect to the prezygapophyses (0), or dorsally raised roughly 20° relative to the coronal plane (1), or dorsally raised at least 30° or more relative to the coronal plane (2). (McPhee and Choiniere, 2017). Ordered.

405. Dorsoventral height of the lacrimal ramus (ventral process) of the prefrontal: more than 0.5 times that of the jugal ramus (ventral ramus) of lacrimal (0), or less than 0.5 times that of the jugal ramus (ventral ramus) of the lacrimal (1) (Chapelle and Choiniere, 2018).

406. Distal end of frontal ramus of postorbital: single rounded process (0), or forked into parietal and frontal processes with a distinct concave notch between them (1) (Chapelle and Choiniere, 2018).

407. Anterior portion of supratemporal fossa on posterior end of dorsal surface of frontal: weakly excavated (0), or deeply excavated forming a scarp-like margin (1) (Chapelle and Choiniere, 2018).

408. Squamosal-quadratojugal contact: point contact or a dorsoventrally oriented short contact (0), or broad contact anteroposteriorly or obliquely oriented (1) (Chapelle and Choiniere, 2018).

409. Angle between quadratojugal and pterygoid rami of quadrate: acute angle, between 0 and 30 degrees (0), or between 30 degrees and 90 degrees (1), or greater tan 90 degrees (2) (Chapelle and Choiniere, 2018). Ordered.

410. Ventral extent of quadrate condyles: both condyles extend to the same ventral level (0), or medial condyle extends farther ventrally (1), or lateral condyle extends farther ventrally (2) (Chapelle and Choiniere, 2018).

411. Shape of anteroventral portion of prootic: rectangular, anteroposteriorly longer than dorsoventrally high (0), or bulbous, almost as anteroposteriorly long as dorsoventrally high (1) (Chapelle and Choiniere, 2018).

412. Angle separating the long axes of the basiperygoid processes in anterior view: 60 degrees or less (0), or more than 60 degrees (1) (Chapelle and Choiniere, 2018).

413. Length of postorbital ramus of laterosphenoid: short, subequal to supraoccipital ramus (0), or  more than 10% longer than supraoccipital ramus (1) (Chapelle and Choiniere, 2018).

414. Orientation of postorbital ramus of laterosphenoid: extends laterally (0), or extends anterodorsally (1) (Chapelle and Choiniere, 2018).

415. Orientation of frontal ramus of laterosphenoid: extends medially (0), or extends anteromedially (1) (Chapelle and Choiniere, 2018).

416. Distal end of jugal ramus of ectopterygoid: tapering (0), or broad subrectangular distal end (1), or expanded anteroposteriorly, forming a T-shaped dorsal and ventral profile (2) (Chapelle and Choiniere, 2018).

417. Serrations on premaxillary teeth: present (0), absent (1).

418. Growth marks in long bones: present in the whole cortex (0), or growth marks absent or only formed in the outer cortex (1). (Cerda et al., 2017)

419. Relative abundance of woven fibered WFB versus parallel fibered bone PFB in the primary compact bone: PFB > WFB (0), WFB > PFB (1). (Cerda et al., 2017).

***10.g. Data Matrix***

xread

419 76

Euparkeria

00000000?0000?0000000?000?100100000000000000000?00000000010010000000000000000?000000??00?000?000000000?1000000000000000000?00000000000???0000??000000000?00?0?00??00000000000000000000000?0?0?????????000?0020000000000??????00000000001?0?0?0000???00?0010?0000?0110000000000010000000000?02?000??11000001?000?100000?0000000??0?00010??0000??0001?100000000?000?????????0?00000?0000000000000?1?000??20?00?0100000000??00????2?[01]0

Crurotarsi

0000000000000?0[01]000000000?0000000000[01]0000000000000000000010000000000000000000?00000??0000000000000000000000000000000000000000000000000???0000??000002000?00?0?00??00002000000000000000000000000000[01]00?0[01]0?002000000000000000000000000000000000000[01]000000010?0000?0[01]00000000000000000000000?00?0000010000001?0000000000?0000000??0?000000000000000000100000000000[01]00?0??00???0?0?0100000000000[01]00?0000?????[01]?01[01]00??0????????????0??

Marasuchus

00???????0?0????????????0?00???????0???????????0???????????????????????????00???0000????????????????????????00?00000000?1?100000000010???00?0??00?000000?00???00??000?00000000?000000000??01010000000?000?0121000?0000??????????????????????????????000000000000?0100010000000000000001000000011000100000000000000001000100100000?00000000000000000?00?0??00???10????????0?0000?0?000000???00000?0000?020??????????0???????????????

Ornithischia

0010000000100?0000000?000?1011000100100?1000000100100001000000[01]000100100000000100001000010010[01]01[01]000000100101001110100001000000000010100[01]0000??010000000?00?0?00?000000000000000[12]10000000000010000100001001111100000100000000000000000000000000000000110112100010001??000?000110000000100001?010111102000000000[01]011110002?01?[01]100000111100000000000[01]0000000000010?000???????000?0001[01][012]000001011010200?????[01]?01?00000?????2??????1[01][01]

Agnosphitys

??????????????????????????????????????????????????????????????????????????????????????????????????????????????????????????????????????????????????????????????????????????????????0?????????????????????????????????????????????????????????????????00000020000100????????????????????????????????????????????????????????????????????????????????????????????????????????????????02???????1???????????????????????????????????????

Silesaurus

00?0??00?0?00?00100000??0?00?0?01010?????????0010??0????00?????????00?0?0?0000??000110?????1?00?0?010???0?0000010001000010?00000000000???0010000000000010100001100000000000000??00000100000?0?0???????111?1110000100000?????????????????????????????000000000000000000000000?010100?0?10000110110001011000100011000000000?0000000??11?1??00??000000?00???000?001100000?0?1?00000??010000???11000?0000????11000000??0?????101?1???0[01]

Neotheropoda

00[01]0[01]002[01]000201001100001[01]000000000000110000000010[01]100[01]01000001[01]0001[012]0100000010110000101110001000000001?00000000000000000101100110000010000[01]1000010200001010110010000001000000000[12]10001100000000010100001000101100000100010001200000010?0001001101[12]010101013000211101010000111110000110100001?011000100000000[01]011011111002001011000011111000?0?000001000000000001200000?1?00?0001000111111[01]120111[01]0000?????[01]?01000000????????????0[01][01]

Staurikosaurus

00???????????????????????????????????????????????????????????????????????????????????????????0000000001000?00?0000000000????????00010????00?01001?000011?000?00000000020000001??010101100000110010????1?1???????????????????????????????????????????000000200110000110?010?00010?00000100001??000?0101?000000001000000001????01?????????????????????????????????1???????????01????01???????1?110?010?00????????????????????????????

Chindesaurus

???????????????????????????????????????????????????????????????????????????????????????????????????????????????????????????????100?????????????01?0??0?1??0?1??????000??00???????????1??0????1?00??????????????????????????????????????????????????????????0???101?1????0??????????????00?0101110001000100??00?10111100???011110100??1??????????????????????????1???????????00?????1???????10?10???00??????????????????????????????

Herrerasaurus

00000000?0001000000000000?00010000000000100000010001010100000100001101000000101?0001000?1000?00000000?1000000000000000001010001100000100000?000010000011??0010[01]10000002000000100000101100000110010100?1110??01100000000000001200000110000011011010010000002000000000000010???01?010100100001011100010110001000010000100010010010100000011000100001000000000000?02000000000?00001000100000111011[01]011000000??0??0?0000101?20?1???1011

Guaibasaurus

??????????????????????????????????????????????????????????????????????????????????????????????????????????????????????????????????????????????????0???01??011001000000??00000??000000?00000?01?0??[01]00??0?????110????1?0???????0????110??0?1?00??0?0?000?00[12]100210100010000000010?1011000000100100001011000100?0?01111100100?01101?001011100000000101000000000001100?00??0?0?0?010102???????111?1111??00?0??????????????????????????

Eoraptor

0001?001?0001000011000111010000010000110100000?101100?0100?01100001101???11?1????????????????10000010???0?0100?10[01]0000001?????110000000000?1011??000??00?00???00?000??00000011??10???100??001?00??000?0000??1?10??00101??????20?00011?00010?00000101101000[12]0002101??0??00?0???10?1011?10??0?0111000101100000?1?00[01]1010??1?0??0?????01????000???00?0000000000??010????????????00100021011110111100110???10?0?????0?00?0?0?1?1???1011

Saturnalia

10????????????????????????1???????00?100?0??????????????????10?????????0???0?0??0101?0??????????0?0?0??????0000100000000????????0010010010??01101?00000100001100000000000000000001000101??0??1000??0??10101111100010100?????????????????????????????00?0001100210000000000100110010010000001011100010110001000000010100010010010100000001000000001010000?000?0010000000000000000??12??000??11111?1100??101?00?0000?0?????????????01

Panphagia

?0???????????????????0?1???????????????????0??1????????1?1?0???????10?0?111????0?????????????1?0?0?1010000?10?0101000000????????00000100011?01101?000?01???01?10000?0?0000000??0???00??????????00??0??000???????????????????????????????????????????0??0??11002100?00??000????1??0??10?????????????????????001000[01]10100???010?1010?????????????0??????????????????????????????????02???????111?1?0?????????????00??0??1?211????????

Chromogisaurus

????????????????????????????????????????????????????????????????????????????????????????????????????????????????????????????????????????????????????????????????????????????????01000??1100??1???????????????????????0??????????????????????????????0????0[12]1002100??????????????????????0??????1???10110??1000000010100010????????????????????????0??0???000???10?????????????????12???????11?11?????0?????????????????????????????

Buriolestes

0?01000210101010011000111000000000000100100000?10110010100001?00001?01100110000???0?0??1??00100000010100000100010000000010100010010001001011?1101000000100011?00000000000000000?0100010?0?000?000??0???1????111010011?0???????????01????????????????00000?10002100?0??0?001?011?0001101000000111000101101000?1?000?01?0?1??1?0?????0100???000??00?0100000000??0100?10??10???????????1????????????????0010?[01]????????0?01?[12]??1???????

Pampadromaeus

0001000210101?10?11000?11000??00100001100000????0?10010??0??1?????110?1??????????????????000???000000??00001100101000000??10001????????????1??????0??001??0?1?10??0000000000[01]0?0??0001?10?0??1000??0??1110??1?101010000?????????????????????????????0000001110?10??????????0?1?????????00001011100010110?0?00?00???????01?????????????????000???0??1?0???0???????0??????0??????????10?100??????????????00???????0??????????????????

Bagualosaurus

10?1?00[12]??0?[12]0???1??????001?000?1000?10??0???0?10010?????????????0??????????????????????????010000010?????01?00101000?00??????????????????????????0??001??0?1???10?0?0000000?0??01?001???????01???0??????????????????????????????????????????????????0?00?1100?10[02]?00?00?0?????????????000??0111000101110?10000000?01??010????????????????0?1??00?0100?????????11??0??110??????????10??0????1???????????0???????????????????????0??

Thecodontosaurus

?0????????????????????????1??1??1000??????0?0?0?0???000?????????????0???0?1010?10011?0???????00?10010??????1100101010000?1??????000101010?1?01101?000001??00110000000000000000?1000?0101100?01000??00?010011211000101001100012010001100001010000010100?00121000001???0?000??011??01010?000011110000101100010000000101100??011010100????110001110?1?000??00?0????1????????????1??00010000??01?110???000?20?000?10???0???????????????

Pantydraco

?0???0????????????[01]00?0???1??1????000100100000010?100001?00????????00?000?1?10??00110011?000?00?00010??0000?1001010100001001??1100?10101001101101?00???1????1???????????????????????0??11?00010000100?????11?1?00???????????????????????????????????00100121100001?????????0011??10110?00??????????1?1?000?0000000?01?001?????????????????001110011?000000001001?0??00000????0????0100??1??1????0??0??0??1100??00??0?0??2101?1?00??

Efraasia

100?1001?000??1?111?112?1110?100???00100100000??0?10??01?0??1??????10?000?1????10011100??????10010010??11?011001010100001???1?11001101001011011010000001??001?00000000000000000110000101000?0110000?0?010011212000111001101012010?11000001110100010000100131000101000010000001100101101000011110000101100010000001101?001?011010?0?01?1110001??0011?0010000010002??0???000?10001000100001??1111??1000002011001?00??0????2?11?0?????

Nambalia

??????????????????????????????????????????????????????????????????????????????????????????????????????????????????????????????????????????????????????????????????????????????????????????????000????????????????????????????????????????0?10??0110??????02?0??10??0??????????1????111?0100110101001011010???????1101?0???011010??00101???00101?011100??00????00100000010????????????????11?10?0?00????????????????????????????????

Jaklapalisaurus

??????????????????????????????????????????????????????????????????????????????????????????????????????????????????????????????????????????????????0???0??????????????????????????????????????1??????????????????????????????????????????????????????????????????????????????????????????????????????????00?000010110110???011010??0???????00??1?0??1?????????????00??101???????????????????????1???????????????????????????????????

Pradhania

????????????????????????0?1???????00??????????????????????????????????????00??????????????????0???1?1??????????1?1010?????????????20??????????10??????????????????????????????????????????????????????????????????????????????????01?????20?0??????????????????????????????????????????????????????????????????????????????????????????????????????????????????????????????????????????????????????????????????????????????????????

Macrocollum

1001100210002111111111211110010010000100100000110010010100001000001101??????101???????0?100??1001011010?1?0110010101000011?0111100200100111101101000000111011101000000000000100110?0010?0000001000100?01001111200001100110111201010101000211000001000010012100010200?0?0001?01100101110100000110000101100010000[01]111011001001101010001011100010100111000000001000100000011001?00000??????????1??????110010???????0?00?1?????????????

Unaysaurus

?001100?1010??1?111??1??1010??011000????????????0??00?????01???000??0?0?????????????10????????0?10110?????01100101010000???0?1?????0?1????????????0??0011100110000000000000???????????????????1000?00?010001?1101001100??????10?0?110????1110??0?????????????????????????????????????????????????????????????????110110???01?010?00?????????11???????0???0???????0????????????????????????????????????01?110??????????1????1???????

Ruehleia

???????????????????????????????????????????????????????????????????????????????????????????????????????????????????????????????10???0????01???101?002001??00110010000011000000?1100001?00?0?0110??????01001111200011100?0100??01011101000???0??00???001001311000020??11000110110?10110?101011010000102100010?00?011011001?000010100?????????????????????????????3?????????0?00010??????????????????0???1???????????0???????????????

Plateosaurus_ingens

????????????????????????????????????????????????????????????????????????????????????????????????????????????????????????????????????????????????????????????????????????????????01??01????????10???????????????????????????????????????????????????????????????????????????????????????????????????????????00?010????????????????????????????????????[01]???0?0????4????????00??00??????????????110???????????????????????????????????

Plateosaurus_gracilis

?00??001?0002?110111?1??1110?101??00?10?1010?0??0?10001100??10??????????????????1?????????????0??0110?????11100101010?001?????1100??01???01101101?00?001?100110000000000000010?1010001010000001000?00????????1200?11100?1011?10101010000???10?????0?001001311001020010100011111001011??00001111000010110?010000?0??0110????????????????????0?????????0?0????????[23]????????001000?000?00001101111?1?01?00????????????0????????????0??

Plateosaurus_engelhardti

1001100110002011[01]11111211110010110000100101010110011001100011001001101100101101110011000101111001011010111111001010100001100111100110100[01]011011010002001110011000000000000001011010001[01]000000010000000010011112000111[01]0110111101011100100201000001000010013110010200101000111110010111100001111000010110001000010111110010011010100010111000110001110010000010004000000111010000000[12]000001011110?1010001002000100000001?01111102001

Glacialisaurus

????????????????????????????????????????????????????????????????????????????????????????????????????????????????????????????????????????????????????????????????????????????????????????????????????????????????????????????????????????????????????????????????????????????????????????0????????????????0????????????????0???1010[01]????11010111?01???????????????101110111?0????????????????????????0???0??????????????????????????

Coloradisaurus

?00?1002??002?111111?1??1110??111000?10??01?10?101110111?000100100?1011011011010100111?????1?1001011010011??000101010001?11?001100200100101?01100?00?001?1001100000000000000101????00?10100?001???????00001011210011?1???????????????1???????????????0?0?131100???011?11002001100101110000011010000102111010000001101100??011110?00??????0001110012000110000210021111101100111????0?0000??????????0?00?101100?100000?1002100001??00

Lufengosaurus

100???02???0211?1?11?1111?111110100101011010000101110101100010?1????0110??01[01]0??1001?1001001010010????0111?11001010100011?00011100100110111?0110100020011100110000000000000010011010011000000[01]10000000001011112[01]1011110110111101113100101201000102000010013110000201101100210110010110000101101000010211101010000110110010011110?0001?1110101110012000110000[12][01]00410111011???00?0000100000??1????1????0010??0????????111??1?????0???

Xixipiosaurus

100?10?????0??111?11?1111?1111?0??000101?11000?10?11?1111?0?1001????01?0??01?01??????????????1?01?010???1??11001???1?????????????????????????????????????????????????????????????????????????????????????????????????????????????????????????????????????????????????????????????????????????????????????????????????????????????????????????????????????????????????????????????????????????????????????????????????00????????????

Massospondylus_carinatus

1001100210002111111111211011111010000101101000110[01]11011110001000102?01101100[01]0110001[01]1?01000010010110101010110[01]1010100011100?111002001001111011010002001110011000000000000001011101001[01]00000000000000?01[01]011112010111[01]01101111011121001012110000020000100131000100011011001001100101110001011010000102101010000001101100100111101000111110001110012000100000210020?00?011001000001?100000????????1???0010100111000000000[01]1000010000

Adeopapposaurus

1001100210002111011111011011111010000101101000110111111111001000102101[01]01100101100011100101000001011[01]10101011111110[01]00011100111100200100011101101100200111001100000000000000100110100100000000010000000000101110001110011011110111210110011100000200001001311001000110110010?11001011000000110100001021010100000011011001001111010001110100011100120001100001100100000011010010000??00000??1?11?11???00101001?100000?010?11100?0000

Leyesaurus

?001?00?1000211??11?????1010?11010000101101?00?1011111111?00100010[02]1010?????????00?11???0??1?00?10110??1010?111101000001?1??1111002001000111011????0???????????????????????????????????0000?001???000??????????????????????????????????????????????????????????????????100?????????????????????????????????????????????????????????????010???????12000????0???0??????00?????????????000??????????????0??0?00??1?0?00?0??11?1?????00

Ngwevu

1?01010??10021111111????111?10101000?10110?000?10?110111111?0001100101001111001??00??111?001011?11010?????011011010100011????111?020?11001110?1??1?00?0???0??????0???0000?0010000???????????????????????0???1121?101??0???????????11?????2010?????????????????????0?1?1100?????????????1010?101000010??1??1???????????0??????0?0?????????0001??0??2?00??000???0???0?????1????????0??????????????????????????????????011011010100???

Plateosauravus

???????????????????????????????????????????????????????????????????????????????????????????????????????????????????????????????10???0100101?01101?00[12]0011?00110000000001000000??10??011???0??110????????0???[23]1110011110????????????1????????????????00100131100101?01????????11??10110?00001111000010110001000000110110??????0???????????????11?0????0??????????4????????????000???????????????????0???2???????????0???????????????

Riojasaurus

1001?00??0002011?110??00111011011000?100001000?10010?10110001000001101?0?100101?0001???????1?001000?1??101?100010101000[01]10??11110011010010110110100010011100110000000000000010?1101001[01]00000011000??0?01001121210111100111111?010111011001010001020?0010013110010201101000110110010110010110111010110201101000000110110010011010100??011?00011100121011[01]00001??13????????0?10100010100000?011110?00110020??0?????000?????????????00

Eucnemesaurus_fortis

??????????????????????????????????????????????????????????????????????????????????????????????????????????????????????????????????????????????????0???01??001100[01]10000??00?0??????????10000?01100?????????11???????????????????????????????????????????????????????110???0??????????????0110111010?1010110?000001110110?????????????????????????????????????????[23]??????????????????????????????????1?0?????????????????????????????

Eucnemesaurus_entaxonis

??????????????????????????????????????????????????????????????????????????????????????????????????????????????????????????????????????????????????0???01??0????0???000??0000?00?1011?0100?0?01??????????????????????????????????????????????????????0????13??[01]0100??1??0?0?0?11?010110?1011011101001010110?0?????110111?1?0??0?????0??????00111?01[12]?0?11000?????20???0?????????????????????????????1???????????????????????????????

Seitaad

??????????????????????????????????????????????????????????????????????????????????????????????????????????????????????????????????????????????????00?[01]?1??00??00?001????0000?????????????????????????0000011?11000111[01]011?11100101310?00?2110??0???0??????????????0?1000001????????????????????????????????0?00?01101?0???01101010?0??11?00?1??001???11[01]0000010???0???0?10??0??001??????????????????????0??????????????????????????

Sarahsaurus

10?1000?10?11010?11?1?1?0110110100000100001010010?110001100010?0???101000111001???01?1001??1?10?10010?????011001010100001?1011110110011101110111110010010100101110000010000010111000011000010010000001000010102100111111101111011021111?011100001200100001311101021001110011111011011102?1001100010101110010000010[01]01000100011101001?1111011?110011000?1000111001??0???11010??000????????????????????0010??????????0010?0??1?1010??

Yunnanosaurus_huangi

100?1002??00??10?110?1??111?111????00101111?00??0?1101111?001000?01?01?0?00010???????????????10010010???1???00010?21000?????111100110110100?01101000?0011?00110000000000000010?0101101[01]0000?00?0??0??000?0??11200?11110??01??0011?2101100201000[01]020?0010013100000001101[01]00[12]0?110010111?0010110100001011010101000011011001?01101010001111?010111001????1?0000110?2?0???11??0??1??010100000?01?1???00??0?10??????????0?000????????1??

Xingxiulong

??????????????????????1???1?111????0?10?00???0110?11001100001000101?01?00100101?2??1?1???????0?????????0[01]1???00?????????1100111100100110001?011010002001110011000000000100001100110??11?1000001????00?0000???120?011110????1??????1101??????????????001001311000020?1011002??11??0001000000111100001011010?00000[01]101110010011010?0?10110001011000121011100002100200000?11????????????????????????????0??0??????????0?00????????????

Jingshanosaurus

1001?002??00211111100???11101[01]101100?11?001??1?10?11011110001000????01100000101?2?1??10?0??1?10110010??11101100101011001????111??010??????1???10100??0011100?1???00000000000?0??10??01100000001000000?0010??11201011110??00??0011?3100100?010001020000100131000002011001002??110010110?00001101000010111101010000110110?12011010?00??????01011110120011[12]0000010?4????????????????1???????????????????0010??????????????????1????1??

Chuxiongosaurus

?00??002??00??111111?1[01]?11101[01]10??00?11100???1??0?1101111?0?1000????01???????????????????????101?00?0??111?1100?0101??????????????????????????????????????????????????????????????????????????????????????????????????????????????????????????????????????????????????????????????????????????????????????????????????????????????????????????????????????????????????????????????????????????????????????????????????0????????????

Yizhousaurus

110110021010211111101111111111011000011100100011001111111100??00???10?100?00101?2?????000001010110010??0000110[01]1010110011?10111100100110101?011010[01]000011100[12]10000000001000010?01000011?000?01???????001001111100011110??????0010?21011011010001020000100?31100000010011002?0110010110?1110111100001011110??????????????????????????????????????????????????????[34]??1???1?????????????????????????????0?2???????????0???????????????

Anchisaurus

10???00??0002?1??11?????111011?010?001010?100011001111011?0?10??????01?001101?1?2001110??????10?10?00??10000000101?1[12]?0111?0011???1001001111011?10000?010?001100?000??0000000??0100?0??0?01?00?10?????10[01]0??211001011?0??0??1101011100100201000002000[01]10113100000?0111100010011?001010000101101000010[01]11101000000110111?1?01?010???10111?0?01??0011?0010000010011??0????10??0?0?01020000??01?11?110100?20?10????000??01????1???????

Mussaurus

10011002?000??111111?11?11101110100001010[01]?000?10011?1011000[01]0?0?01?0?1??????????????????????10101111???1?0??0?1?1111001????????00100????0?10110??0000?10000100000000?00000010??111?011?1?0001110?????0010?011100111110?1?11?00101210110120100000200???0113100010?011??1001?011??10110?10101101000010111101000000110111011011010001??????01011100120011100001100300?00111111?011010[12]???????111??11?????10??????????0?01?????????1[01][01]

Leonerasaurus

?0????????????????????????????????????????????????????????????????????????????????????????????????0?0???????1?11010010011???001??010?1???????1100?00?0?10000110000000??00000100?111001????????????????0000???1100000?????????????????????????????????1101?3100?????????0?????11??1?1??????????????????????????????????????????????????????10111??????????0???????0??0???1?0????????1???????1???????????????????????[01]????????????100

Sefapanosaurus

????????????????????????????????????????????????????????????????????????????????????????????????????????????????????????????????00??01100???0?10??00?001????1?00000001??0000????1?????11?00??011????0?0?0010?1?00????10?1011??010131010??1010???????????????????????????????????????????0?111110000??11[01]???000000??????0?00110101010001??0???11?0?2?0????????????00?0????011?01111?????????????????????????????????????????????????

Aardonyx

1101?00210002?11?11?????1110?1011000??????1010??0?11010??10?1????????1?0???????????????????1??0?0?1?0?????11?00101011001?1??????0010?110100101101?0000011?00110000000020000010?1100?0110000?01110??00?????11?????????10??00??????13101???1?10??10?????????????????1110100010011??10110?101?111100001111000???????1101110?0???01??????????010111?01?0?2??00?0???0[34]???00011???0?111????????????????????0??0??????????0???????????????

Irisosaurus

?????????011??1???1?????10????1??????????????????????????????????????????????????????????????????????????????0?10?21100?????????00???????????110??00?001???????00?0???0?0?00???????????????????????????1?010??1??01?1?0??????1010121011012010000020???????????????????????????1??10110???????????????????????????????????????????????????????????????????0?0???????????????????????????????????????????0???????????????????????????

NMRQ3314

1001?1031000211111101?1110101111???1010101101011011101011?00000010220110110010????0??10011011001?00101?1000110?10021100110?01011001?01101001011[01]10000001??0??100??0?0?10000000??111001??010?0?1??0?????1????[23]1100?0?110??????0010?2100101[12]0?0001020000100?31000[01]0[12]????????????1??10110?????????????1???11010000011?011?01?0??0?0???0011100101111012?011[12]00001100301100?11????????????????????????????0002?[01]0???????000?????1???????

NMQR1551

?????????????????????????????????????????????????????????????????????????????????????????????????????????????????????????????????????????????11???00?001??00210000000010000000??2?10012?000?0[01]1100????0100??2????000110???????????21????????????????00100?3100000[01]011010001????????????11101111001110[01]111010000011101100100?10101000??????1011100????1120000????3001?0?11????????????????????????????00?1??????????????????????????

Meroktenos

????????????????????????????????????????????????????????????????????????????????????????????????????????????????????????????????????????????????????????????????????????????????????????????????????????????????????????????????????????????????????0??0?131?00001011?00001????????????111111100?001111110???????????????????????????????????01??????????????????1??0???????0??????????????????????????????????????????????????????

Ingentia

???????????????????????????????????????????????????????????????????????????????????????????????????????????????????????????????????111????01011?0??0?????????????????????????????????????????????????????????11[01]0010110?1?10?00111310110???????????0??????????????????????????????????????????????????????????????????????????????????????????????????????????????????????????111????????????????????????????????????????????????01

Lessemsaurus

????????????????????????????????????????????????????????????????????????????????????????????????????????????????????????????????0?011110100??1101?0000011100[12]10000010121001000????????????????????????0001???11[01]0010110???????0?1?310?????0?0??1????0010013100000?011110001???1??10110?111011?10???11111?0?000000100101???011011101??????????11??12101??00??0???311?????10?201??1001?????1?1111??1010????????????????????????????01

Antetonitrus

?1?????????????????????????????????????????????????????????????????????????????????????????????????????????????1?1012000????????0?[01]0???????????00?000001??00[12]10001010121001000???1????100?0?01100???0?0011??311000[01]0110??????0011?3101???1010???????00100?3???0002011010001????????????11101111001111111101000000100111010????????????????1111110????[12]1200?0????31?100??1?0?00111????????????110??0????2???????????????????????????

Ledumahadi

????????????????????????????????????????????????????????????????????????????????????????????????????????????????????????????????????????????????1?0??0011???[12]?0000010121001000????1???1??00?01???????????????????????10???????????[23]1????????????????????????????????????????????????????0???????????????????????????????????????????????????????????????0???????[45]??0???1?????????????????????????????0?????????????0???????????????

Kholumolumo

????????????????????????????????????????????????????011?????????????????????????????????????????????????????????????????????????0101?1?1????0??01?00?0011???2??0000101??????????????0110??0?111100????010????11000[01]??00???????????2101???1?11???????10000131110000010?10001????????????00011110000011110?1?0010010001000?1?????????????????0111??1200???????????31?000011?1?001010???????????????????????????????????????????????01

Blikanasaurus

???????????????????????????????????????????????????????????????????????????????????????????????????????????????????????????????????????????????????????????????????????????????????????????????????????????????????????????????????????????????????????????????????????????????????????????????????????????0000?0110111?1?0100101001011100101111112102120000100?211?0?1?11?0?0??????????????????????0???0??????????????????????????

Camelotia

???????????????????????????????????????????????????????????????????????????????????????????????????????????????????????????????????1???????????0??0??001??002100000000??00?0??????????1???0??011???????????????????????????????????????????????????????????????????1??????????1??00111?111011110111?1??110?000000????????????????????????????????????[12]???000????5??????????????????????????????????1???????????????????????????????

Schleitheimia

????????????????????????????????????????????????????????????????????????????????????????????????????????????????????????????????0??????????????01?10?0111?0???????000??1??????????????????1??110?????????????????0?0??????????????????????????????????????3111[02]?02???????????????????????????????????01110??????????????????????????????????????????????0???????4??1???????????????????????????????????????????????????????????????

Pulanesaura

???????????????????????????????????????????????????????????????????????????????????????????????????????????????10?012011????????001?11?0100??11?????0??1??00?0010?01?0001000????????112?11011???0??????????????10??1?????????????????????????????????????????????????????0?11??1?11???????????????????????00000100101?????????????????????????????????1????????????0?????????????????????????????????1?????????????1???????????????

Gongxianosaurus

1?????0??????????12???????????????????????????????????????????????????????????????????????????????????????00???1??2121????????????0????????????1??0??001??0??????0?0??00000????????????0??0?0?1?0?000?011???2110????1?1?????????????????????????????0?100????0?????????????????????????[12]111?2??????1???1101??????1?0??1???0?1?1????1?11??010???10?2?1112000020005??????????????????????????????????????22??????????????????????????

Isanosaurus

????????????????????????????????????????????????????????????????????????????????????????????????????????????????????????????????0??0???????????2??10??0???????????????2?011000???????????????????????101?0?????????????????????????????????????????????????????????????????????????????211112??0???101?1?0??????????????????????????????????????????????????????3?110??1??????????????????????0????1???????????????????????????????

Tazoudasaurus

11??????????????????????????????????????????????????010??1?????????[02]011???1???1??????????????00?00000???0??10?1111012101???000??001010??????0002??100001??00200100011020121000????????211?1?111????11?????01?1100100?2????????010?210000100?0001?310?1???03101??0?111001002??11??1??1??21100111001101011?0?0010000101010?1111011111?011??????????????1??10?1????51??0????1?0??1001?????????????????10??????????????2???????????????

Vulcanodon

????????????????????????????????????????????????????????????????????????????????????????????????????????????????????????????????????????????????????????????[12]????????????????????1??????0111?0?00?0?0??01???2110?10?021???????????????????????????????????310[12]?00?01111000100111110010?111011110010011?1??20?10001?0???11?111011?111011??11?01010120111110?121?05?1??00?11?00000?????????????1???11?01?21??????????????????????????

Shunosaurus

11000113?110201101200000001011?20100111201010101001101012000011011121010001011?1200?????110200100000120100011111112121112??01011?1001?1?10010012?1100001011021?00001?0201210000?11?00?100111011001?111?1?000211001100?11000?1011001?000110000??1?3101111013101100011111?01100?111100100211112??0???000?01121010?01?0????110?101???11011??1111101002?12111?1021105???????????0?00020?00000??1?10???0??1121???0??11?12?0???1?101??1??

Spinophorosaurus

1????1?????????????????????????????????2????????????0????01?????????0??101111111[12]?0???????????1?????????????100111012?112?????11?11011?0?01?0002?110??01????[12]?11???1?12?1010?0????????[12]1??11011?00?1??1110?03?[12]0?10??[12]???????????????????????????????????????[12]????1?1???01[12]??011??001??211111110?1101??0?020?100???0??11?10??0?0?01???????????????????????????????????????????????????????????????????1???21?0?1?010??1????1???????

Patagosaurus

11????????????????????????????????????????????????????????????????????????????????????????????1?00001??????0??0111012111??????1100101100000110020?2001010?00201?01111120121?00?0210011[12]00?1?0?100???0?111?00?1100100?[12]1?????????????????????????????1111013101000011111001100111110010?211112??0???0011111?101000??0????????????????????????????????????????????5???????????0?????02???????1?11???110??????????????2?????????????11

Barapasaurus

???????????????????????????????????????????????????????????????????????????????????????????????????????????????1?111211???????????1?1????00??002??100101??00201101111120121100?0[12]1001?10?11??1????????101?00?1???????11?????????????????????????????11110131011000111110011???11110010???????????????????1?1?1?????????1?1???????1?????????0???????????2?1??????5????????0000?000?01???????0?11?1?1?01?????????????????????????????

Cetiosaurus

1????????????????????????????????????????????????????????11?????????????????????????????????????????????????????????????2??0011110111110100100020?2001?10?1020??1011102010100???[12]?????2001??00100???011110103110010002????????1?????????????????????1111013?0210?0110?1?011??1101?00???211112??0???010?111210100010010111???????????????????????????????????????5?????????1?000??????????????????????1?2???????????0???????????????

Omeisaurus

110001131111201101200000101011?201001112010101011111010121100000112221110010111???0??0???1???11101001?0?00011101111121113??001110120011010111002?12101010110201100011020121100?021??1?20011100100?011111100031100100021??????0100001000110001001?31011110131011000111110011000111100100211012??0???010?01021010011?0??111?011010?11??1????1111011020121111102?105?????????0?0?0?0???00000??1?1????1??1?2???????????0?0???1?????????

Mamenchisaurus

11000113?11120110120000000101112??001112010101011111110121100000111221010000111?201??0?????2?11[01]0000100100011111111121113?0001110121010000110002?1110101010020110001102012100000110111211111011001011111100031100100120110011011000100011?0?10?1?31?11110131011?00111110011000110100100211112??0???010?01121010?1??0??????101011111??????1101??11?2?121111102??05?1?????????0?00?????????????????????11211?1?0??1010?0?1?1?0???????

Neosauropoda

1100011311112011012000001010111[02]11101112010101011[01]11110121100[01][01]011222111001011112[01][01]0[01]000010201110[01]001201000[01]111111[02]12[01]112[01]00001110011110100110020121010101[01]0201110011020121100002100112001111[01]10000100111000311001[01]00211100010100000000110001??1?30011110131021000111110011001110[01]10[01]00211012??0???010?11121010?11000011111010111111011??111100110201211111021105?11???110?20?00020200000?01?100??110????[01][012]1000[01]111????1[12]1?[01]???[12]?11

;

ccode + 7 12 18 22 39 56 68 91 101 107 116 120 133 143 146 148 149 156 166 169 170 176 182 204 206 213 221 226 241 250 253 276 298 335 341 348 352 369 392 403 408 *;

proc/;

**11. References**

Alonso-Zarza, A.M. 2003. Palaeoenvironmental significance of palustrine carbonates and calcretes in the geological record. Earth-Science Reviews 60, 261–298

Apaldetti, C.G, Pol, D., Yates, A.M., 2012. The postcranial anatomy of *Coloradisaurus brevis* (Dinosauria: Sauropodomorpha) from the Late Triassic of Argentina and its phylogenetic implications. Palaeontology 56, 277–301.

Bapst, D.W. 2012. paleotree: an R package for paleontological and phylogenetic analyses of evolution. Methods in Ecology and Evolution 3, 803–807.

Bates, K. T., Falkingham, P. L., Macaulay, S., Brassey, C., & Maidment, S. C. 2015. Downsizing a giant: re-evaluating Dreadnoughtus body mass. Biology letters 11, 20150215.

Bell, M.A., Lloyd, G.T. 2015. strap: an R package for plotting phylogenies against stratigraphy and assessing their stratigraphic congruence. Palaeontology 58, 379–389.

Behrensmeyer, A.K. 1978. Taphonomic and ecologic information from bone weathering. *Paleobiology* 4, l50–l62.

Bronzati, M., Rauhut, O.W. 2017. Braincase redescription of *Efraasia minor* Huene, 1908 (Dinosauria: Sauropodomorpha) from the Late Triassic of Germany, with comments on the evolution of the sauropodomorph braincase. Zoological Journal of the Linnean Society 182, 173–224.

Bronzati, M., Rauhut, O.W., Bittencourt, J.S., Langer, M.C., 2017. Endocast of the Late Triassic (Carnian) dinosaur *Saturnalia tupiniquim*: implications for the evolution of brain tissue in Sauropodomorpha. Scientific reports 7 (11931), 1–7. doi: 10.1038/s41598-017-11737-5.

Campione, N. E. 2017. Extrapolating body masses in large terrestrial vertebrates. Paleobiology, 43, 693–699.

Campione, N. E., Evans, D. C., Brown, C. M. and Carrano, M. T. 2014. Body mass estimation in non-avian bipeds using a theoretical conversion to quadruped stylopodial proportions. Methods in Ecology and Evolution, 5(9), 913-923.

Carpenter, K. 2005. Experimental investigation of the role of bacteria in bone fossilization. Neues Jahrbuch für Geologie und Paläontologie Abhandlungen 2, 83–94

Cerda I.A., Chinsamy A., Pol D., Apaldetti C., Otero A., Powell J.E. 2017. Novel insight into the origin of the growth dynamics of sauropod dinosaurs. PLoS ONE 12(6), e0179707. doi: 10.1371/journal.pone.0179707

Chapelle K.E.J., Choiniere, J.N. 2018. A revised cranial description of *Massospondylus carinatus* Owen (Dinosauria: Sauropodomorpha) based on computed tomographic scans and a review of cranial characters for basal Sauropodomorpha. PeerJ 6, e4224. Doi: 10.7717/peerj.4224

Chapelle, K. E., Barrett, P. M., Botha, J., & Choiniere, J. N. 2019. *Ngwevu intloko*: a new early sauropodomorph dinosaur from the Lower Jurassic Elliot Formation of South Africa and comments on cranial ontogeny in *Massospondylus carinatus*. PeerJ 7, e7240.

Cohen, K. M., Finney, S. C., Gibbard, P. L., & Fan, J. X. 2013. The ICS international chronostratigraphic chart. Episodes, 36 199–204.

Downing, K.F., & Park, L.E., 1998. Geochemistry and Early Diagenesis of Mammal-Bearing Concretions from the Sucker Creek Formation (Miocene) of Southeastern Oregon: Palaios, v. 13, p. 14–27.

Ezcurra, M.D., 2010. A new early dinosaur (Saurischia: Sauropodomorpha) from the Late Triassic of Argentina: a reassessment of dinosaur origin and phylogeny. J. Systemat. Paleontol. 8, 371–425.

Freytet, P. and Verrecchia E. P. 2002. Lacustrine and palustrine carbonate petrography: an overview Journal of Paleolimnology 27, 221–237.

Goloboff P.A., Farris J.S., Nixon K.C. 2008. TNT, a free pro- gram for phylogenetic analysis. Cladistics 24, 1–13.

Goloboff P.A., Szumik C.A. 2015. Identifying unstable taxa: efficient implementation of triplet-based measures of stability, and comparison with Phyutility and RogueNaRok. Molecular Phylogenetics and Evolution **88,** 93–104.

Hesselbo, S.P., Ogg, J.G., Ruhl, M., Hinnov, L.A. & Huang, C.J. 2020 Chapter 26 - The Jurassic Period. In *Geologic Time Scale 2020* (eds. F.M. Gradstein, J.G. Ogg, M.D. Schmitz & G.M. Ogg), pp. 955-1021, Elsevier.

Jaffey, A. H., K. F. Flynn, L. E. Glendenin, W. C. Bentley, A. M. Essling. 1971. Precision Measurement of Half-Lives and Specific Activities of 235U and 238U. *Physical Review C* **4**, 1889–1906.

Jalfin, G.A., Herbst, R., 1995. La Folra Triásica del Grupo El Tranquilo, Povincia de Santa Cruz (Patagonia). Estratigrafía. Ameghiniana 32, 211–229.

Li, Y., Zhang, W., Aydin, A., Deng, X., 2018. Formation of calcareous nodules in loess–paleosol sequences: Reviews of existing models with a proposed new “per evapotranspiration model”. Journal of Asian Earth Sciences 154, 8–16.

Lyman, R.L. 1994: *Vertebrate Taphonomy*. *Cambridge University Press, Cambridge*, 524 pp.

McPhee, B.W., Choiniere, J.N. 2017. The osteology of *Pulanesaura eocollum*: implications for the inclusivity of Sauropoda (Dinosauria). Zoological Journal of the Linnean Society 182, 830–861.

McPhee, B.W., Choiniere, J.N., Yates, A.M., Viglietti, P.A. 2015a. A second species of *Eucnemesaurus* Van Hoepen, 1920 (Dinosauria, Sauropodomorpha): new information on the diversity and evolution of the sauropodomorph fauna of South Africa's lower Elliot Formation (latest Triassic), Journal of Vertebrate Paleontology 35. DOI: 10.1080/02724634.2015.980504

McPhee B.W., Bordy, E.M., Sciscio, L., Choiniere, J.N., 2017. The sauropodomorph biostratigraphy of the Elliot Formation of southern Africa: Tracking the evolution of Sauropodomorpha across the Triassic–Jurassic boundary. Acta Palaeontologica Polonica 62, 441–465.

Novas, F.E., Ezcurra, M.D., Chatterjee, S., Kutty, T.S., 2011. New dinosaur species from the Upper Triassic Upper Maleri and Lower Dharmaram formations of central India. Earth and Environmental Science Transactions of the Royal Society of Edinburgh 101, 333–349.

Otero, A., Pol, D., 2013. Postcranial anatomy and phylogenetic relationships of *Mussaurus patagonicus* (Dinosauria, Sauropodomorpha). Journal of Vertebrate Paleontology 33, 1138–1168.

Otero, A., Krupandan, E., Pol, D., Chinsamy, A., Choiniere, J.N., 2015. A new basal sauropodiform from South Africa and the phylogenetic relationships of basal sauropodomorphs. Zoological Journal of the Linnean Society 174, 589–634.

Otero, A., Cuff, A. R., Allen, V., Sumner-Rooney, L., Pol, D., & Hutchinson, J. R. 2019. Ontogenetic changes in the body plan of the sauropodomorph dinosaur *Mussaurus patagonicus* reveal shifts of locomotor stance during growth. Scientific reports, 9, 1–10.

Pol, D., Escapa, I. E. 2009. Unstable taxa in cladistic analysis: Identification and the assessment of relevant characters. Cladistics 25, 515–527.

Pol, D., Otero, A., Apaldetti, C. & Martínez, R. N. 2021. Triassic sauropodomorph dinosaurs from South America: The origin and diversification of dinosaur dominated herbivorous faunas. J. South Am. Earth Sci. 107, 103145.

Smith, N.D., Pol, D., 2007. Anatomy of a basal sauropodomorph dinosaur from the Early Jurassic Hanson Formation of Antarctica. Acta Palaeontologica Polonica 52, 657–674.

Uliana, M. T., Biddle, K. T. 1987. Permian to Late Cenozoic evolution of northern Patagonia: main tectonic events, magmatic activity, and depositional trends. Gondwana six: structure, tectonics, and geophysics 40, 271–286.

Yates, A.M., 2007. The first complete skull of the Triassic dinosaur *Melanorosaurus* Haughton (Sauropodomorpha: Anchisauria). Special Papers in Palaeontology 77, 9–5.
